# Clinical and Economic Impact of a First Major Bleeding Event in Non-Anticoagulated Patients in Spain: A 3-Year Retrospective Observational Cohort Study

**DOI:** 10.3390/jcm14041377

**Published:** 2025-02-19

**Authors:** Carlos Escobar, Beatriz Palacios, Miriam Villarreal, Martín Gutiérrez, Margarita Capel, Ignacio Hernández, María García, Laura Lledó, Juan F. Arenillas

**Affiliations:** 1 Cardiology Department, University Hospital La Paz, 28046 Madrid, Spain; 2 AstraZeneca Farmacéutica, 28050 Madrid, Spain; beatriz.palacios@astrazeneca.com (B.P.); miriamvillarealdm@gmail.com (M.V.); martin.gutierrez@astrazeneca.com (M.G.); margarita.capel@astrazeneca.com (M.C.); 3 Atrys Health, 28002 Madrid, Spain; ihernandez@atryshealth.com (I.H.); mgmarquez@atryshealth.com (M.G.); llledo@atryshealth.com (L.L.); 4 Neurology Department, Comprehensive Stroke Center, Hospital Clínico Universitario, 47003 Valladolid, Spain; juanfarenillas@gmail.com; 5 Clinical Neurosciences Research Group, Department of Medicine, University of Valladolid, 47003 Valladolid, Spain

**Keywords:** cost, healthcare resource utilization, major bleeding

## Abstract

**Objective:** To analyze clinical characteristics of non-anticoagulated subjects with major bleeding, and to determine the incidence of adverse events, healthcare resource utilization (HCRU) and associated costs following a major bleeding event. **Methods**: Retrospective observational cohort study that analyzed secondary data from electronic health records in Spain. Non-anticoagulated patients with a first major bleeding during the study period (between January 2013 and December 2022) were analyzed for 3 years. **Results**: A total of 4089 patients (mean age 57.26 (12.87) years, 58.47% female) were included. A proportion of 27.63% presented with genitourinary bleeding, 22.43% with gastrointestinal bleeding, 5.16% with respiratory bleeding and 3.11% with intracranial hemorrhage. At the end of the first major bleeding event, 0.56% of patients died (5.51% after intracranial hemorrhage, 3.23% in case of trauma-related bleeding). The incidence rates of clinical outcomes per 100 person-years within the first 3 months of the major bleeding were death from any cause 7.51 (95% CI 6.70–8.32), cardiovascular death 1.80 (95% CI 1.39–2.21), acute myocardial infarction 4.53 (95% CI 3.89–5.17), and ischemic stroke 3.52 (95% CI 2.96–4.08), and decreased over time. At year 3, mean overall major bleeding cost per patient was EUR 13,310.00 (5153.05), of which EUR 7648.20 (2674.46) (57.46%) accounted for in-hospital costs to treat the major bleeding event. **Conclusions**: Among non-anticoagulated patients presenting with a first major bleeding, <1% of patients died during index hospitalization. However, these patients had a substantial risk of adverse clinical events during the follow-up, as well as high associated HCRU and costs.

## 1. Introduction

Major bleeding is associated with increased morbidity and mortality and also with higher healthcare resource utilization (HCRU) and associated costs [1,2]. In addition, the proper approach to patients with major bleeding is difficult and challenging [3,4]. However, the majority of the information on patients with major bleedings emerges from patients taking anticoagulants [5,6,7].

Remarkably, information about the clinical profile, risk of outcomes and costs of patients presenting with major bleeding but not receiving anticoagulants is very scarce and generally limited to patients with particular conditions (i.e., antiphospholipid syndrome, cardiovascular disease, low-risk atrial fibrillation, etc.), or with a specific type of major bleeding (gastrointestinal, genitourinary, trauma related, or intracranial, among others) [8,9,10,11,12,13,14,15,16,17,18,19,20,21,22]. As a result, contemporary data for this clinical setting are warranted.

We have presented the results of a cohort study, showing the incidence rates of major bleeding among patients taking direct Factor Xa inhibitors in Spain and, more recently, the risk of adverse clinical events, HCRU and costs of patients with major bleeding during direct Factor Xa inhibitor use [23,24]. The aim of the present study was to ascertain the clinical characteristics of non-anticoagulated individuals with a major bleeding, and to determine the incidence of adverse events (all-cause mortality, cardiovascular death, acute myocardial infarction, ischemic stroke, acute kidney and liver failures), as well as HCRU and related costs over a period of 3 years following a major bleeding.

## 2. Methods

This was a retrospective, observational cohort study, using secondary data from the electronic health records of the BIG-PAC^®^ database. This database includes data from electronic medical records (hospital and primary care centers) of seven Autonomous Communities in Spain since 2012 and has been validated in many studies [25,26,27].

This study included all adults with a first major bleeding event during the study period (between 1st January 2013 and 31st December 2022) and without any anticoagulant use in therapeutic or prophylactic doses prior to the first major bleeding event, as per the time intervals defined in the exclusion criteria. In addition, patients had to have ≥12 months of data availability in the database at their start of observation. By contrast, patients taking anticoagulants at therapeutic or prophylactic doses in the 60 days before the major bleeding event, with a history of a major bleeding event at any time before the start of the study period (i.e., 2013), with palliative care initiation at any time before the major bleeding event, being pregnant at the moment of major bleeding event, or with a history of significant bleeding diathesis (i.e., hemophilia, immune thrombocytopenia, Von Willebrand disease) were excluded from the study. The study was approved by the Research Ethics Committee of Consorci Sanitari de Terrassa, Barcelona, Spain, which waived the requirement for written informed consent, as this observational and non-interventional study used only secondary anonymized data.

Major bleeding was defined as all critical site bleeding or, for other locations, bleeding that was fatal or led to hospitalization. Major bleeding was categorized as gastrointestinal, intracranial, or other life-threatening bleeding (genitourinary, respiratory, and other bleeding not included elsewhere) according to the International Classification of Diseases (ICD) Tenth Revision (ICD-10). Trauma-related ICD-10 bleeding codes are a subset of the intracranial, or other, bleeding codes (Supplementary Table S1). In addition, Supplementary Table S1 includes all the ICD-10 codes for major bleeding classified as “other bleeding not included elsewhere”. In this category, 98.3% of major bleeding was of the Unspecified subtype. The remainder were hemopericardium (1.46%) or hemarthrosis (0.23%) [23,24]. The start of the observation period (index date) was considered as the day of the first major bleeding. The study period ended in case of any of the following: 3 years after the index major bleeding event, pregnancy, finalization of the study period, disenrollment from the database, conclusion of data collection, patient death, or initiation of palliative care. For HCRU and event rates, follow-up continued until the finalization of the study period.

Baseline data (on the day prior to index day) were collected: bio-demographics (age, sex, body mass index, alcohol use, Charlson comorbidity index) cardiovascular risk factors, vascular disease, and other comorbidities. Clinical conditions were defined via the medical codes entered by practice, using the ICD, Ninth Revision (ICD-9), and ICD-10 (all converted to ICD-10 in the database). Biochemical parameters nearest to the index date were extracted (hemoglobin, HbA1c, platelet count, renal function). In addition, concomitant treatments taken within 120 days prior to the index date were also recorded (non-steroidal anti-inflammatory drugs, antidiabetic drugs, gastroprotective agents, antiplatelet drugs, antihypertensive therapies, lipid lowering therapies). History of anticoagulant for 60 days prior to the index date was also evaluated. Treatments were derived from issued prescriptions, coded utilizing anatomical therapeutic chemical codes (ATC medication code: “A10”). Data were analyzed in the overall study population, and according to major bleeding type.

Cumulative clinical outcomes, including all-cause death, death from cardiovascular causes, acute myocardial infarction, ischemic stroke, acute kidney failure or acute liver failure were assessed in the overall study population and according to the first major bleeding type, estimating the incidence and event rates. Incidence rates were calculated as the total number of incident events divided by the total person time at risk. Event rates were defined as the total number of events, including recurrent events, divided by the total person time of follow-up, during the entire study period. Cumulative incidence curves for clinical events (all-cause death, death from cardiovascular causes, acute myocardial infarction, ischemic stroke) were determined in the overall study population and according to major bleeding type.

Cumulative HCRU and costs were calculated, and included outpatient visits (general practitioner (GP) and specialist), investigations (laboratory and radiology), hospitalizations, prescriptions and work absences. All-cause HCRU and major bleeding-related HCRU were estimated in the overall study population and according to major bleeding type at 6 months, 1 year, 2 years and 3 years after the index episode of major bleeding. Information on cost data was obtained from the eSalud database and costs of prescriptions were based on the full price of drugs [28,29]. Costs of work absence were calculated by multiplying the number of days of absence from work due to illness by the mean daily salary of a worker in Spain [30] (Supplementary Table S2). No other indirect costs were considered.

## 3. Statistical Analysis

According to the original design of the study, the results shown were descriptive [31]. Qualitative variables were defined by their absolute (n) and relative (%) frequencies. Quantitative variables were described with mean and standard deviation (SD). Incidence and event rates for clinical outcomes were described per 100 person-years with their 95% confidence intervals. Cumulative incidence curves for clinical events (acute myocardial infarction, stroke, death from cardiovascular causes and all-cause death) were also estimated. To determine HCRU after major bleeding, cumulative HCRU rates were calculated, as well as their respective costs by multiplying event numbers by costs per event. Statistical analyses were made using Stata MP Version 14.2 (StataCorp LLC., College Station, TX, USA).

## 4. Results

A total of 4089 patients with a first major bleeding were included and followed up during a 3-year period. The mean age (SD) was 57.26 (12.87) years old, 58.47% were women, 14.67% had hypercholesterolemia, 11.08% hypertension and 4.26% type 2 diabetes. Regarding vascular disease, 13.4% had heart failure, 2.71% prior myocardial infarction and 1.66%, prior stroke. Moreover, 21.4% had a history of anemia and 10.56% cancer. Regarding treatments, 5.48% were taking non-steroidal anti-inflammatory drugs, 2.98% antiplatelets and 4.79% had a history of anticoagulant use at any time prior to the 60-day period before major bleeding. Respecting the type of major bleeding, 27.63% presented with genitourinary bleeding, 22.43% with gastrointestinal bleeding (with upper bleeding being more common than lower bleeding), 5.16% with respiratory bleeding, 3.11% with intracranial hemorrhage (with similar presence of subarachnoid and intracerebral bleedings), and other bleedings accounted for 41.67% (of which 98% were unspecified). Comparing the baseline clinical profiles according to the major bleeding type, patients with respiratory bleeding were older. Women had more frequent genitourinary bleeding, whereas gastrointestinal and intracranial hemorrhages were more common in men. The Updated Charlson comorbidity index was higher in patients presenting with intracranial hemorrhage. History of prior stroke was more frequent among patients with intracranial hemorrhage and peptic ulcer disease in those with gastrointestinal bleeding (Table 1 and Table S3).

Cumulative clinical outcomes (incidence and event rates) during the follow-up were analyzed in the overall study population and according to the major bleeding type (Table 2 and Table S4). At the end of the first major bleeding event, only 0.56% of patients had died (5.51% after intracranial hemorrhage, 3.23% in the case of trauma-related bleeding, 1.90% after respiratory bleeding, 0.55% in the case of gastrointestinal bleeding, 0.18% after genitourinary bleeding, and 0.29% after other major bleeding events). The incidence rates per 100 person-years within the first 3 months of the major bleeding were death from any cause 7.51 (95% CI 6.70–8.32), cardiovascular death 1.80 (95% CI 1.39–2.21), acute myocardial infarction 4.53 (95% CI 3.89–5.17), and ischemic stroke 3.52 (95% CI 2.96–4.08). These numbers were 2.49 (95% CI 2.01–2.97), 0.70 (95% CI 0.44–0.96), 2.18 (95% CI 1.73–2.63) and 1.77 (95% CI 1.37–2.17) per 100 person-years at 3 years of follow-up, respectively. The incidence and event rates for clinical outcomes decreased over time and were extremely low for acute kidney or liver failures. Incidence rates for all-cause death, death from cardiovascular causes, acute myocardial infarction and ischemic stroke were higher in those patients presenting with intracranial hemorrhage, whereas incidence rates for clinical events were lower among those with genitourinary bleeding. No clinically relevant differences in clinical outcomes were observed across the subtypes of gastrointestinal or intracranial bleeding (Supplementary Table S4). Cumulative incidence curves for events (acute myocardial infarction, stroke, death from cardiovascular causes and all-cause death) were calculated for the overall study population and according to the type of major bleeding (Supplementary Figures S1–S7).

The cumulative HCRU for 3 years from the index date was determined overall and by major bleeding type, including outpatient (GP and specialist) visits, laboratory/radiology investigations, hospitalization, length of hospital stays, number of prescriptions and work absences (Table 3 and Table 4 and Supplementary Tables S5 and S6). After 3 years from the index event, overall all-cause HCRU rates for outpatient visits, laboratory/radiology investigations and hospitalization were 566.82 (95% CI 551.63–582.01), 108.42 (95% CI 98.89–117.95) and 50.35 (95% CI 48.82–51.88) per 100 person-years, respectively, with a decrease over time from the index date. For major bleeding related HCRU, these numbers were 230.26 (95% CI 217.36–243.16), 64.34 (95% CI 62.87–65.81) and 41.66 (95% CI 40.15–43.17) per 100 person-years, respectively, with a decrease over time from the index date. All-cause and major bleeding related HCRU were numerically higher among those patients with intracranial hemorrhage (e.g., major bleeding related hospitalization rates at 3 years were 50.66 vs. 41.75, 39.10, 42.20 or 42.66 for gastrointestinal, genitourinary, respiratory or other major bleedings, respectively; *p* = 0.298). Major bleeding-related length of hospital stay and mean number of days (SD) due to work absences per patient were 9.55 (3.64) and 17.95 (12.10) days, respectively, in the overall group. By major bleeding type, the intracranial hemorrhage is that with the numerically greatest duration of hospitalization (10.65 vs. 9.28, 9.66, 9.22 or 9.58 days for gastrointestinal, genitourinary, respiratory or other major bleedings, respectively; *p* = 0.685) and significantly more work absence (21.69 vs. 17.79, 19.99, 15.03 or 16.78 days for gastrointestinal, genitourinary, respiratory or other bleedings, respectively).

Cumulative costs for 3 years from the index date were calculated in the overall population and according to major bleeding type (Table 5 and Table S7). The total mean overall costs per patient at year 3 were EUR 17,010.6 (7616.47), and 13,646.4 (5699.97) within the first 6 months. The equivalent mean major bleeding-related costs per patient were EUR 13,310.0 (5153.05), and 12,231.1 (4304.38), respectively. Within the first 6 months after the index event, 58.85% of total costs accounted for hospitalizations, 37.77% for indirect costs (cost of absence from work), 5.83% for prescriptions and 3.37% for outpatient care. At study end, these numbers were 53.78%, 34.43%, 6.39% and 11.78%, respectively. With regard to major bleeding-related costs, within the first 6 months after the index event, 58.35% of total costs accounted for hospitalizations, 38.34% for indirect costs, 5.42% for prescriptions and 3.31% for outpatient care. At study end, these numbers were 57.46%, 36.97%, 5.31% and 5.57%, respectively. Mean major bleeding-related costs were higher among those patients with intracranial hemorrhage (at 3 years: EUR 15,220.04 vs. 13,191.39, 13,902.95, 12,202.84 or 12,975.28 for gastrointestinal, genitourinary, respiratory or other major bleedings, respectively.

## 5. Discussion

Our data showed, in a wide sample of non-anticoagulated patients presenting with major bleeding, that mortality was low during the index hospitalization, but the incidence rates of clinical events, HCRU and costs were substantial after 3 years of follow-up. In contrast to other studies performed with anticoagulated patients, this study provides a comprehensive, original and complete view of the clinical and economic impact of a first major bleeding event in non-anticoagulated patients [23,24].

In our study, mean age was 57.3 years, 58.5% were women and the proportion of patients with comorbidities was relatively low (i.e., 13.4% had heart failure, 11.1% hypertension, 10.6% history of cancer, 4.3% type 2 diabetes, and 2.7% prior myocardial infarction). When compared to other studies with patients with major bleeding but without a compelling indication for anticoagulation, the clinical profile of patients was quite similar to our cohort [10,12,13,14,15,16,17,21,22]. However, patients with an indication requiring anticoagulation, such as atrial fibrillation or venous thromboembolism, are markedly older and have more comorbidities. For example, in the case of patients with atrial fibrillation, in clinical trials comparing direct Factor Xa inhibitors vs. warfarin, the mean age ranged between 70 and 73 years, 35–40% of patients were women, 90–94% had arterial hypertension, 25–40% type 2 diabetes and 35–63% heart failure [32,33,34]. In a real-life population, when analyzing those patients, with a first major bleeding event while on direct Factor Xa inhibitors, from the BIG-PAC^®^ database in Spain, mean age was 78 years, 61% of patients were women, most had atrial fibrillation (78%) and patients presented with many comorbidities (79% had hypertension, one third type 2 diabetes, and 24% heart failure) [24]. Therefore, our study presents unique information, as it provides a comprehensive view of the clinical profile and risk of events of non-anticoagulated patients with major bleeding, not limited to specific populations, conditions or type of major bleeding.

Many studies have focused on patients with a specific major bleeding site, but not on patients having any type of major bleeding [10,12,13,14,15,16,17,21,22]. Thus, in our study, around 28% of patients presented with genitourinary bleeding, 22% with gastrointestinal bleeding, 5% with respiratory bleeding, 3% with intracranial hemorrhage and 42% with bleedings in other sites (of which 98.3% in the database were classed as unspecified bleeding). Respiratory bleeding was associated with older age and history of chronic pulmonary disease, genitourinary bleeding was more frequent in women, intracranial hemorrhage was linked with a more frequent history of prior stroke, hypertension or with a higher Charlson comorbidity index, and history of peptic ulcer disease and excessive alcohol use were more common in gastrointestinal bleeding. As a result, it is important to consider the clinical history of patients to predict the risk and site of major bleeding in order to take different measures to prevent its development, including a close follow-up of these patients, or better management of these conditions (i.e., blood pressure control) [35,36].

At the end of the first major bleeding event, only 0.56% of patients had died. This means that, except for patients presenting with intracranial bleeding (5.5%) or trauma-related bleeding (3.2%), mortality during the index event was low. These numbers are much lower compared to patients taking oral anticoagulants. For example, among patients with a first major bleeding event taking Factor Xa inhibitors from the BIG-PAC^®^ database, 4.26% of patients died at the end of the first major bleeding event, 70.0% in case of trauma-related bleeding, 28.13% after intracranial hemorrhage, and 2.15% after gastrointestinal bleeding [24]. In the clinical trials comparing direct Factor Xa inhibitors vs. warfarin, the proportion of fatal bleeding during the study was 0.4–0.46% with direct Factor Xa inhibitors and 0.8–0.85% with warfarin [32,33,34].

Despite the fact that, in our study, mortality rates at the end of the first major bleeding event were low, these patients had a high risk of adverse clinical events, although this risk decreased over time. Thus, the incidence rates of death from any cause decreased from 7.51 within the first 3 months of major bleeding to 2.49 per 100 person-years at 3 years of follow-up. For cardiovascular mortality these numbers decreased from 1.8 to 0.7 per 100 person-years, respectively, for acute myocardial infarction, from 4.53 to 2.18 per 100 person-years, respectively, and for ischemic stroke, from 3.52 to 1.77 per 100 person-years, respectively. As expected, these numbers were higher in those patients presenting with intracranial hemorrhage. In real-life patients taking factor Xa inhibitors from the same database and study period, these figures were 16.42, 9.3, 7.34 and 5.42 per 100 person-years at 3 years of follow-up, respectively [24]. Therefore, regardless of whether the patient is taking anticoagulants or not, physicians should not only be aware of the risk of a rebleed, but also of other clinical events, highlighting the fact that these patients require a comprehensive approach to all comorbidities to decrease the risk of developing complications during the follow-up [35,36]. Mortality was particularly high in those patients that presented an intracranial hemorrhage, mainly within the first 3 months of follow-up (41 vs. 6 per 100 person-years after 3 years since index date), indicating that, after discharge, these patients still have a high risk of adverse events and mortality and close follow-up should be performed through comprehensive management that ensures the control of those conditions that enhance the risk of complications, such as hypertension [37].

It is well established that HCRU is high after major bleeding in anticoagulated patients, greater with warfarin than with direct oral anticoagulants [6,24]. Our study shows that, although lower than those reported in anticoagulated patients, high HCRU is also associated with major bleeding even in non-anticoagulated patients. The same trend was observed with cumulative costs during the follow-up, with a total mean overall cost per patient at year 3 of EUR 17,010 and mean major bleeding-related costs per patient of EUR 13,310. Of note, 50–60% of total costs corresponded to hospitalizations and around 35–40% to costs related to absence from work. Interestingly, indirect costs were higher in non-anticoagulated patients compared to patients receiving anticoagulation, since anticoagulated patients are usually older, and consequently many of them were retired [24]. This means that, to reduce HCRU and costs, it is necessary to improve the management of major bleeding during the index event and to perform a comprehensive approach to control all comorbidities during follow-up.

In contrast to bleeding events related to oral anticoagulants, in which specific and non-specific reversal agents together with supportive measures are commonly used and interventional procedures can be made, in major bleeding not related to oral anticoagulants the use of reversal agents is not applicable. A previous study of the BIG-PAC database showed that the major bleeding related cost per patient was EUR 12,376 (79.7%1 for inpatient bleeding costs) among patients receiving direct factor Xa inhibitors [24]. Our study showed that, in non-anticoagulated patients, this cost was EUR 13,310.00, 57% accounted for by in-hospital costs to treat the major bleeding episode. Different studies have shown that the use of specific reversal agents is a cost-effective approach in anticoagulated patients [38,39]. In non-anticoagulated patients, supportive measures (i.e., fluid replacement, blood transfusion), as well as interventional procedures to treat bleeding cause if necessary (i.e., gastroscopy, surgery) are recommended by international guidelines. After major bleeding, it is mandatory to intensify efforts to modify bleeding risk factors [16,36].

This study has some limitations that deserve commentary. It had a retrospective design. Consequently, only those variables included in the electronic health records could be recorded and could be used for analysis. Additionally, no sensitivity analyses of missing data were conducted. However, the high sample size and the global view of the study, including all major bleeding, regardless of the bleeding site, could reduce this potential bias. On the other hand, patients with a history of significant bleeding diathesis (i.e., hemophilia, immune thrombocytopenia, Von Willebrand disease) were excluded from the study. Although some patients could have had other minor or rare coagulopathies and congenital ones, it is expected that either no patient or an extremely low number of patients would suffer from these rare coagulopathies, leading to practically the same results. Finally, our results can only be extended to patients with similar clinical profiles and to similar healthcare systems.

In conclusion, among patients presenting with a first major bleeding, but without anticoagulation, less than 1% of patients died during index hospitalization. However, these patients had a substantial risk of adverse clinical events during follow-up, as well as high associated HCRU and costs. These data suggest that better management of major bleeding during the index event and an improved comprehensive approach to all comorbidities during follow-up are warranted to reduce the burden of major bleeding.

## Figures and Tables

**Table 1:** Baseline clinical characteristics on the day prior to index day (day of the first major bleeding) in the overall study population and according to the type of major bleeding.

	Overall (N = 4089; 100%)	GIB (N = 917; 22.4%)	ICH (N = 127; 3.1%)	Genitourinary (N = 1130; 27.6%)	Respiratory (N = 211; 5.2%)	Other (N = 1704; 41.7%)
**Biodemographic data**						
Age, years (SD)	57.26 (12.87)	57.59 (11.33)	55.73 (11.7)	54.37 (10.97)	62.02 (12.67)	58.52 (14.43)
<45 years, n (%)	520 (12.72)	95 (10.36)	20 (15.75)	168 (14.87)	12 (5.69)	225 (13.2)
45–64 years, n (%)	2594 (63.44)	627 (68.38)	82 (64.57)	780 (69.03)	125 (59.24)	980 (57.51)
65–74 years, n (%)	511 (12.5)	114 (12.43)	16 (12.6)	122 (10.8)	43 (20.38)	216 (12.68)
75–84 years, n (%)	297 (7.26)	57 (6.22)	6 (4.72)	41 (3.63)	22 (10.43)	171 (10.04)
≥85 years, n (%)	167 (4.08)	24 (2.62)	3 (2.36)	19 (1.68)	9 (4.27)	112 (6.57)
Sex (female), n (%)	2391 (58.47)	331 (36.1)	42 (33.07)	761 (67.35)	95 (45.02)	1162 (68.19)
BMI, kg/m^2^ (SD)	28.8 (12.43)	28.89 (16.25)	28.35 (14.69)	28.99 (18.03)	27.43 (14.21)	28.82 (16.81)
Alcohol use, n (%)	71 (1.74)	25 (2.73)	4 (3.15)	13 (1.15)	4 (1.9)	25 (1.47)
Updated Charlson comorbidity index	0.35 (0.73)	0.5 (0.81)	1.16 (0.48)	0.19 (0.50)	0.27 (0.77)	0.33 (0.77)
**Cardiovascular risk factors**						
Hypertension, n (%)	453 (11.08)	82 (8.94)	24 (18.9)	108 (9.56)	34 (16.11)	205 (12.03)
Hypercholesterolemia, n (%)	600 (14.67)	127 (13.85)	9 (7.09)	197 (17.43)	28 (13.27)	239 (14.03)
Diabetes type 2, n (%)	174 (4.26)	28 (3.05)	4 (3.15)	39 (3.45)	6 (2.84)	97 (5.69)
Diabetes type 1, n (%)	18 (0.44)	2 (0.22)	1 (0.79)	6 (0.53)	0 (0)	9 (0.53)
Smoking, n (%)	117 (2.86)	37 (4.03)	4 (3.15)	39 (3.45)	6 (2.84)	31 (1.82)
**Vascular disease**						
Heart failure, n (%)	548 (13.4)	114 (12.43)	14 (11.02)	267 (23.63)	19 (9.00)	134 (7.86)
Chronic kidney disease, n (%)	19 (0.46)	2 (0.22)	1 (0.79)	2 (0.18)	1 (0.47)	13 (0.76)
Myocardial infarction, n (%)	111 (2.71)	32 (3.49)	6 (4.72)	22 (1.95)	4 (1.90)	47 (2.76)
Stroke, n (%)	68 (1.66)	1 (0.11)	52 (40.94)	4 (0.35)	1 (0.47)	10 (0.59)
Peripheral vascular disease, n (%)	29 (0.71)	3 (0.33)	1 (0.79)	4 (0.35)	1 (0.47)	20 (1.17)
Venous thromboembolism, n (%)	4 (0.1%)	1 (0.11%)	0 (0%)	1 (0.09%)	0 (0%)	2 (0.12%)
Atrial fibrillation, n (%)	50 (1.22%)	15 (1.64%)	3 (2.36%)	3 (0.27%)	0 (0%)	29 (1.70%)
Non-mechanical cardiac-valve replacement, n (%)	3 (0.07%)	1 (0.11%)	0 (0%)	0 (0%)	0 (0%)	2 (0.12%)
**Other comorbidities**						
Anemia, n (%)	875 (21.4)	43 (4.69)	3 (2.36)	102 (9.03)	8 (3.79)	719 (42.19)
Any malignancy, n % (%)	432 (10.56)	106 (11.56)	13 (10.24)	101 (8.94)	29 (13.74)	183 (10.74)
Chronic pulmonary disease, n (%)	343 (8.39)	69 (7.52)	6 (4.72)	98 (8.67)	25 (11.85)	145 (8.51)
Peptic ulcer disease, n (%)	268 (6.55)	262 (28.57)	0 (0)	1 (0.09)	1 (0.47)	4 (0.23)
Dementia, n (%)	18 (0.44)	2 (0.22)	1 (0.79)	2 (0.18)	0 (0)	13 (0.76)
Moderate/severe liver disease, n (%)	9 (0.22)	4 (0.44)	0 (0)	0 (0)	1 (0.47)	4 (0.23)
**Biochemical parameters**						
Hemoglobin, g/dL (SD)	13.62 (2.10)	14.65 (7.32)	14.72 (5.82)	14.14 (7.18)	14.58 (7.11)	12.75 (6.54)
HbA1c, % (SD)	5.86 (0.95)	5.75 (1.81)	6.75 (2.03)	5.79 (1.82)	5.75 (1.81)	5.9 (2.13)
Platelet count, ×10^3^/μL (SD)	395.96 (5870.3)	249.28 (129.55)	236.71 (97.2)	261.09 (139.77)	245.8 (126.26)	557.31 (6248.36)
eGFR, mL/min/1.73 m^2^ (SD)	112.26 (56.68)	122.85 (43.05)	128.44 (34.83)	109.88 (43.45)	130.09 (42.2)	107.52 (41.75)
Creatinine clearance, mL/min (SD)	91.14 (16.38)	90.98 (39)	90.08 (32.72)	92.15 (38.98)	90.35 (36.92)	90.77 (40.62)
**Concomitant treatments (within 120 days prior to index)**						
NSAIDs, n (%)	224 (5.48)	39 (4.25)	2 (1.57)	80 (7.08)	13 (6.16)	90 (5.28)
Prior AC (ended >60 days prior to index), n (%)	196 (4.79)	33 (3.6)	8 (6.3)	45 (3.98)	12 (5.69)	98 (5.75)
Antidiabetic drugs, n (%)	185 (4.52)	30 (3.27)	5 (3.94)	42 (3.72)	6 (2.84)	102 (5.99)
Gastroprotective agents, n (%)	171 (4.18)	34 (3.71)	6 (4.72)	33 (2.92)	11 (5.21)	87 (5.11)
Antiplatelet drugs, n (%)	122 (2.98)	26 (2.84)	4 (3.15)	41 (3.63)	4 (1.9)	47 (2.76)
Antihypertensive therapies, n (%)	111 (2.71)	23 (2.51)	6 (4.72)	21 (1.86)	3 (1.42)	58 (3.40)
Lipid lowering therapies, n (%)	93 (2.27)	24 (2.62)	2 (1.57)	24 (2.12)	4 (1.90)	39 (2.29)

Quantitative variables are presented as mean plus standard deviation; qualitative variables are presented as absolute and relative (%) frequencies. AC: anticoagulant; BMI: body mass index; eGFR: Estimated glomerular filtration rate; GIB: gastrointestinal bleeding; ICH: intracranial hemorrhage; MB: major bleeding; NSAIDs: non-steroidal anti-inflammatory drugs.

**Table 2:** Cumulative clinical outcomes in the overall study population and according to the first major bleeding type.

Time Window ^1^	Overall (N = 4089)			GIB (N = 917)			ICH (N = 127)			Genitourinary (N = 1130)			Respiratory (N = 211)			Other MB (N = 1704)		
	n	%	Rates (95% CI)	n	%	Rates (95% CI)	n	%	Rates (95% CI)	n	%	Rates (95% CI)	n	%	Rates (95% CI)	n	%	Rates (95% CI)
**Incidence Rates ^2^**																		
**3 months since index date**																		
Death from any cause	75	1.83%	7.51 (6.7–8.32)	17	1.85%	7.58 (5.87–9.29)	12	9.45%	41.06 (32.5–49.62)	13	1.15%	4.69 (3.46–5.92)	6	2.84%	11.78 (7.43–16.13)	27	1.58%	6.48 (5.31–7.65)
Cardiovascular death	18	0.44%	1.80 (1.39–2.21)	4	0.44%	1.78 (0.92–2.64)	2	1.57%	6.84 (2.45–11.23)	4	0.35%	1.44 (0.75–2.13)	1	0.47%	1.96 (0.09–3.83)	7	0.41%	1.68 (1.07–2.29)
AMI	45	1.10%	4.53 (3.89–5.17)	9	0.98%	4.03 (2.76–5.3)	9	7.09%	31.42 (23.35–39.49)	9	0.80%	3.26 (2.22–4.3)	1	0.47%	1.97 (0.09–3.85)	17	1.00%	4.1 (3.16–5.04)
Ischemic stroke	35	0.86%	3.52 (2.96–4.08)	10	1.09%	4.48 (3.14–5.82)	3	2.36%	10.27 (4.99–15.55)	6	0.53%	2.17 (1.32–3.02)	0	0.00%	0 (0–0)	16	0.94%	3.86 (2.95–4.77)
Acute kidney failure	1	0.02%	0.10 (0–0.2)	0	0.00%	0 (0–0)	0	0.00%	0 (0–0)	0	0.00%	0 (0–0)	0	0.00%	0 (0–0)	1	0.06%	0.24 (0.01–0.47)
Acute liver failure	5	0.12%	0.50 (0.28–0.72)	0	0.00%	0 (0–0)	0	0.00%	0 (0–0)	2	0.18%	0.72 (0.23–1.21)	0	0.00%	0 (0–0)	3	0.18%	0.72 (0.32–1.12)
**3 years since index date**																		
Death from any cause	291	7.12%	2.49 (2.01–2.97)	71	7.74%	2.72 (1.67–3.77)	20	15.75%	6.00 (1.87–10.13)	42	3.72%	1.27 (0.62–1.92)	16	7.58%	2.67 (0.49–4.85)	142	8.33%	2.94 (2.14–3.74)
Cardiovascular death	82	2.01%	0.70 (0.44–0.96)	20	2.18%	0.77 (0.2–1.34)	2	1.57%	0.60 (0–1.94)	12	1.06%	0.36 (0.01–0.71)	4	1.90%	0.67 (0–1.77)	44	2.58%	0.91 (0.46–1.36)
AMI	248	6.07%	2.18 (1.73–2.63)	59	6.43%	2.32 (1.35–3.29)	14	11.02%	4.36 (0.81–7.91)	59	5.22%	1.83 (1.05–2.61)	11	5.21%	1.87 (0.04–3.7)	105	6.16%	2.23 (1.53–2.93)
Ischemic stroke	202	4.94%	1.77 (1.37–2.17)	40	4.36%	1.56 (0.76–2.36)	8	6.30%	2.46 (0–5.15)	47	4.16%	1.45 (0.75–2.15)	15	7.11%	2.6 (0.45–4.75)	92	5.40%	1.95 (1.29–2.61)
Acute kidney failure	4	0.10%	0.03 (0–0.08)	1	0.11%	0.04 (0–0.17)	0	0.00%	0 (0–0)	0	0.00%	0 (0–0)	0	0.00%	0 (0–0)	3	0.18%	0.06 (0–0.18)
Acute liver failure	27	0.66%	0.23 (0.08–0.38)	0	0.00%	0 (0–0)	2	1.57%	0.60 (0–1.94)	11	0.97%	0.33 (0–0.66)	0	0.00%	0 (0–0)	14	0.82%	0.29 (0.03–0.55)

N: number of patients in the total group or subgroup or with the event; Incidence rates (95 confidence interval): per 100 person-years; qualitative variables are presented as absolute and relative (%) frequencies; ^1^ Time window: cumulative events from index date (day of the first major bleeding); ^2^ Rates: incidence rates defined as the total number of incident events of interest divided by the total person time at risk. AMI: acute myocardial infarction; GIB: gastrointestinal bleeding; ICH: intracranial hemorrhage; MB: major bleeding.

**Table 3:** Cumulative outpatient visits and hospitalization for 3 years from index date in the overall study population and according to the first major bleeding type.

Time Window	Overall Group (N = 4089)		GIB (N = 917; 22.4%)		ICH (N = 127; 3.1%)		Genitourinary (N = 1130; 27.6%)		Respiratory (N = 211; 5.2%)		Other (N = 1704; 41.7%)	
	Patients (n)	Rate, Number of Visits per 100 Patient-Years (95% CI)	Patients (n)	Rate, Number of Visits per 100 Patient-Years (95% CI)	Patients (n)	Rate, Number of Visits per 100 Patient-Years (95% CI)	Patients (n)	Rate, Number of Visits per 100 Patient-Years (95% CI)	Patients (n)	Rate, Number of Visits per 100 Patient-Years (95 CI)	Patients (n)	Rate, Number of Visits per 100 Patient-Years (95 CI)
Cumulative HCRU												
6 months since index date												
All-cause HCRU												
**Outpatient Visits ^1^**	4000	783.60 (770.98–796.22)	900	781.05 (754.28–807.82)	122	803.02 (733.85–872.19)	1098	707.12 (680.59–733.65)	211	924.55 (888.91–960.19)	1669	817.38 (799.04–835.72)
GPs visits	3857	595.44 (580.4–610.48)	873	591.77 (559.96–623.58)	116	616.79 (532.23–701.35)	1047	537.12 (508.05–566.19)	209	706.27 (644.81–767.73)	1612	621.25 (598.22–644.28)
Specialist visits	3190	188.16 (176.18–200.14)	724	189.28 (163.93–214.63)	95	186.23 (118.52–253.94)	842	170 (148.1–191.9)	174	218.28 (162.54–274.02)	1355	196.13 (177.28–214.98)
**Investigations ^2^**	4089	255.92 (242.54–269.3)	917	255.33 (227.11–283.55)	127	283.62 (205.22–362.02)	1130	254.2 (228.81–279.59)	211	268.73 (208.91–328.55)	1704	253.88 (233.21–274.55)
**Hospitalization**	4089	244.4 (231.23–257.57)	917	243.14 (215.37–270.91)	127	292.16 (213.07–371.25)	1130	238.14 (213.3–262.98)	211	247.39 (189.17–305.61)	1704	245.55 (225.11–265.99)
Major bleeding-related HCRU												
**Outpatient Visits ^1^**	4000	744.36 (730.99–757.73)	900	741.38 (713.04–769.72)	122	768.85 (695.53–842.17)	1098	673.23 (645.88–700.58)	211	869.26 (823.77–914.75)	1669	776.41 (756.63–796.19)
GPs visits	3857	556.20 (540.97–571.43)	873	552.10 (519.91–584.29)	116	582.62 (496.85–668.39)	1047	503.23 (474.08–532.38)	209	650.97 (586.65–715.29)	1612	580.29 (556.86–603.72)
Specialist visits	3190	188.16 (176.18–200.14)	724	189.28 (163.93–214.63)	95	186.23 (118.52–253.94)	842	170 (148.1–191.9)	174	218.28 (162.54–274.02)	1355	196.13 (177.28–214.98)
**Investigations ^2^**	4089	218.22 (205.56–230.88)	917	220.53 (193.69–247.37)	127	228.95 (155.88–302.02)	1130	214.95 (191–238.9)	211	224.1 (167.83–280.37)	1704	217.68 (198.09–237.27)
**Hospitalization**	4089	216.58 (203.95–229.21)	917	217.21 (190.52–243.9)	127	257.99 (181.89–334.09)	1130	209.6 (185.87–233.33)	211	217.31 (161.66–272.96)	1704	217.92 (198.32–237.52)
3 years since index date												
All-cause HCRU												
**Outpatient Visits ^1^**	4089	566.82 (551.63–582.01)	917	570.09 (538.05–602.13)	127	596.85 (511.54–682.16)	1130	516.21 (487.07–545.35)	211	636.33 (571.42–701.24)	1704	588.89 (565.53–612.25)
GPs visits	4088	426.14 (410.98–441.3)	916	428.41 (396.38–460.44)	127	453.86 (367.27–540.45)	1130	388.68 (360.26–417.1)	211	472.87 (405.5–540.24)	1704	442.76 (419.18–466.34)
Specialist visits	4044	140.68 (130.02–151.34)	909	141.68 (119.11–164.25)	122	142.99 (82.11–203.87)	1114	127.53 (108.08–146.98)	211	163.46 (113.56–213.36)	1688	146.14 (129.37–162.91)
**Investigations ^2^**	4089	108.42 (98.89–117.95)	917	109.1 (88.92–129.28)	127	122.01 (65.09–178.93)	1130	102.35 (84.68–120.02)	211	116.09 (72.87–159.31)	1704	110.31 (95.44–125.18)
**Hospitalization**	4089	50.35 (48.82–51.88)	917	50.31 (47.07–53.55)	127	57.26 (48.66–65.86)	1130	47.41 (44.5–50.32)	211	50.54 (43.79–57.29)	1704	51.88 (49.51–54.25)
Major bleeding-related HCRU												
**Outpatient Visits ^1^**	4089	230.26 (217.36–243.16)	917	232.93 (205.57–260.29)	127	245.52 (170.66–320.38)	1130	211.47 (187.66–235.28)	211	254.53 (195.75–313.31)	1704	237.57 (217.36–257.78)
GPs visits	4079	174.62 (162.98–186.26)	916	176.6 (151.92–201.28)	127	180.76 (113.83–247.69)	1126	158.87 (137.56–180.18)	211	194.15 (140.78–247.52)	1699	181.47 (163.17–199.77)
Specialist visits	4089	55.63 (54.11–57.15)	917	56.33 (53.12–59.54)	127	64.75 (56.44–73.06)	1130	52.6 (49.69–55.51)	211	60.38 (53.78–66.98)	1704	56.11 (53.75–58.47)
**Investigations ^2^**	4089	64.34 (62.87–65.81)	917	64.16 (61.06–67.26)	127	71.35 (63.49–79.21)	1130	61.76 (58.93–64.59)	211	69.39 (63.17–75.61)	1704	65.09 (62.83–67.35)
**Hospitalization**	4089	41.66 (40.15–43.17)	917	41.75 (38.56–44.94)	127	50.66 (41.96–59.36)	1130	39.1 (36.25–41.95)	211	42.2 (35.54–48.86)	1704	42.66 (40.31–45.01)

^1^ Outpatient visits: including GP visits and specialized visits; ^2^ Laboratory/radiology investigations. Qualitative variables are presented as absolute and relative (%) frequencies. Rate (95% CI): per 100 person-years. GIB: gastrointestinal bleeding; GP: general practitioners; HCRU: healthcare resource utilization; ICH: intracranial hemorrhage; MB: major bleeding.

**Table 4:** Cumulative length of hospital stays, number of prescriptions and work absences for 3 years from index date in the overall study population and according to the first major bleeding type.

	Overall Group (N= 4089)		GIB (N = 917; 22.4%)		ICH (N = 127; 3.1%)		Genitourinary (N = 1130; 27.6%)		Respiratory (N = 211; 5.2%)		Other (N = 1704; 41.7%)	
Time Window ^1^	Average	Standard Deviation	Average	Standard Deviation	Average	Standard Deviation	Average	Standard Deviation	Average	Standard Deviation	Average	Standard Deviation
**Cumulative HCRU per patient**												
**6 months since index date**												
**All-cause HCRU**												
Length of hospital stays (days)	10.02	3.80	9.96	3.74	11.61	6.30	10.20	3.69	9.51	3.44	9.87	3.67
Number of Prescriptions	5.33	1.99	5.32	1.98	5.73	2.23	5.36	1.97	5.44	1.98	5.28	2.00
Work absences (days)	18.81	13.09	19.17	12.70	23.61	18.31	20.87	12.17	15.38	13.16	17.31	13.11
**Major bleeding-related HCRU**												
Length of hospital stays (days)	8.91	2.55	8.90	2.49	10.25	5.27	9.00	2.23	8.45	2.23	8.82	2.48
Number of Prescriptions	1.64	0.98	1.61	0.96	1.78	1.10	1.67	0.97	1.74	0.94	1.62	0.98
Work absences (days)	17.11	10.81	17.43	10.33	21.31	16.21	18.75	9.49	14.19	11.39	15.90	11.05
**3 years since index date**												
**All-cause HCRU**												
Length of hospital stays (days)	11.33	5.28	10.74	4.75	12.13	6.48	11.53	5.11	10.54	4.70	11.54	5.59
Number of Prescriptions	21.27	4.02	20.50	4.42	21.83	3.72	21.38	3.80	21.73	3.75	21.51	3.95
Work absences (days)	21.37	16.92	20.35	14.61	24.46	18.84	23.97	16.29	16.87	15.19	20.52	18.25
**Major bleeding-related HCRU**												
Length of hospital stays (days)	9.55	3.64	9.28	3.16	10.65	5.57	9.66	3.47	9.22	3.67	9.58	3.80
Number of Prescriptions	1.75	0.92	1.73	0.91	1.94	1.00	1.75	0.92	1.83	0.86	1.74	0.92
Work absences (days)	17.95	12.10	17.79	10.89	21.69	16.54	19.99	11.44	15.03	12.64	16.78	12.43

^1^ Index date: day of the first major bleed. Quantitative variables are presented as mean plus standard deviation; qualitative variables are presented as absolute and relative (%) frequencies. GIB: gastrointestinal bleeding; GP: general practitioners; HCRU: healthcare resource utilization; ICH: intracranial hemorrhage; MB: major bleeding.

**Table 5:** Cumulative costs for 3 years from index date in the overall study population and according to the first major bleeding type.

Cumulative Costs	Overall Group		GIB		ICH		Genitourinary		Respiratory		Other	
	Mean Costs per Patient (Eur)	SD Costs per Patient (Eur)	Mean Costs per Patient (Eur)	SD Costs per Patient (Eur)	Mean Costs per Patient (Eur)	SD Costs per Patient (Eur)	Mean Costs per Patient (Eur)	SD Costs per Patient (Eur)	Mean Costs per Patient (Eur)	SD Costs per Patient (Eur)	Mean Costs per Patient (Eur)	SD Costs per Patient (Eur)
**6 months since index date**												
**All-cause costs**												
**Outpatient**	460.5	204.1	460.7	194.4	449.0	191.8	424.7	188.5	524.0	209.0	477.2	215.3
GPs visit	188.0	102.6	186.7	96.6	182.3	99.1	170.9	97.0	221.2	98.6	196.3	108.1
Specialist visit	219.2	142.5	220.3	140.0	203.1	138.9	199.5	135.7	252.3	154.1	228.7	145.4
Investigations ^1^ (outpatient)	11.1	23.8	10.5	23.1	13.4	26.2	11.1	24.2	14.3	25.9	10.9	23.4
Prescriptions (outpatient)	42.3	25.9	43.2	26.7	50.3	33.5	43.2	24.9	36.2	22.8	41.3	25.7
**Inpatient (hospital + all other costs in hospitalization)**	8031.1	2772.1	8098.6	2723.4	9291.0	4551.8	8154.6	2693.0	7507.4	2530.0	7883.7	2672.2
Hospitalizations (>24 h)	7189.4	2726.8	7145.5	2681.6	8329.6	4519.3	7319.7	2649.6	6826.5	2467.0	7086.7	2632.3
Investigations ^1^ (within hospital)	88.2	67.9	102.5	79.5	101.3	80.6	83.5	62.5	85.8	63.8	83.0	62.6
Prescriptions (within hospital)	753.4	508.3	850.6	536.8	860.1	576.5	751.4	510.5	595.1	458.9	714.1	480.9
**Pharmacy and Investigation (global)**	895.0	518.9	1006.8	545.8	1025.1	579.8	889.2	519.7	731.5	472.9	849.3	491.1
Prescriptions (global)	795.7	512.5	893.8	540.4	910.5	577.0	794.6	514.3	631.4	462.9	755.4	485.7
Investigations ^1^ (global)	99.3	71.7	113.0	82.2	114.7	84.7	94.6	66.7	100.1	67.9	93.9	67.1
**Indirect cost**												
Cost of absence from work	5154.8	3587.5	5253.6	3480.6	6471.4	5018.1	5720.6	3335.1	4215.9	3606.8	4744.6	3593.4
**Total Overall cost**	13,646.4	5700.0	13,812.9	5562.2	16,211.5	9159.5	14,299.9	5540.1	12,247.4	5381.2	13,105.5	5468.1
**Major bleeding-related costs**												
**Outpatient**	404.9	194.4	405.5	185.2	386.4	185.3	369.8	178.9	467.3	194.1	421.6	205.5
GPs visits	175.6	92.8	174.2	87.5	172.2	91.3	160.1	87.9	203.9	87.5	183.4	97.7
Specialist visits	219.2	142.5	220.3	140.0	203.1	138.9	199.5	135.7	252.3	154.1	228.7	145.4
Investigations ^1^ (outpatient)	3.9	14.4	4.0	14.6	3.1	13.0	3.6	13.8	5.4	16.7	4.0	14.6
Prescriptions (outpatient)	6.2	9.8	6.9	10.6	8.0	12.0	6.6	10.0	5.5	8.4	5.5	9.2
**Inpatient—Bleeding episode (hospital + all other costs in bleeding event hospitalization)**	7136.8	1908.1	7230.9	1851.5	8172.2	3813.2	7200.8	1693.6	6676.6	1689.0	7023.6	1854.8
Hospitalizations (>24 h)	6396.8	1832.4	6389.5	1785.0	7357.6	3785.7	6461.0	1603.4	6061.2	1598.8	6328.1	1782.1
Investigations ^1^ (within hospital)	83.5	60.7	97.1	71.1	93.6	69.7	78.8	55.4	81.4	57.5	78.9	56.5
Prescriptions (within hospital)	656.5	529.0	744.4	569.9	721.0	585.4	661.0	531.4	534.1	467.8	616.7	499.7
**Pharmacy and Investigation (global)**	750.2	536.3	852.4	576.2	825.8	589.7	750.0	538.9	626.5	475.5	705.0	505.6
Prescriptions	662.7	531.1	751.3	572.2	729.0	585.9	667.7	533.5	539.8	470.3	622.1	501.6
Investigations ^1^ (global)	87.5	62.4	101.1	72.8	96.8	72.0	82.3	56.6	86.7	58.8	82.9	58.5
**Indirect cost**												
Cost of absence from work	4689.3	2961.5	4777.8	2830.5	5841.3	4442.8	5137.8	2601.1	3889.9	3121.6	4357.4	3028.3
**Total Overall bleeding cost**	12,231.1	4304.4	12,414.2	4064.5	14,400.0	7943.0	12,708.4	3841.7	11,033.8	4367.5	11,802.6	4231.3
**3 years since index date**												
**All-cause costs**												
**Outpatient**	2004.6	689.4	2009.3	634.3	1976.4	658.2	1877.5	604.6	2231.3	687.6	2060.4	756.0
GPs visit	779.4	321.7	780.6	298.6	764.4	315.8	727.1	293.2	861.5	317.7	804.4	346.9
Specialist visit	949.4	424.1	952.6	396.6	888.6	425.4	880.2	384.7	1098.9	433.9	979.7	453.1
Investigations ^1^ (outpatient)	95.2	74.4	95.1	73.8	98.3	68.4	90.2	71.3	104.9	79.6	97.2	76.2
Prescriptions (outpatient)	180.5	121.8	180.9	146.7	225.1	165.7	180.0	103.5	166.1	119.8	179.1	113.8
**Inpatient**	9149.1	4108.6	8834.8	3722.8	9777.0	4737.7	9271.9	3903.4	8363.8	3617.4	9287.2	4416.2
Hospitalizations (>24 h)	8127.7	3790.9	7709.0	3410.5	8708.2	4647.9	8277.5	3670.7	7564.6	3375.8	8280.3	4013.4
Investigations ^1^ (within hospital)	115.4	102.2	132.3	115.8	136.7	123.5	108.4	92.7	114.1	97.4	109.6	97.9
Prescriptions (within hospital)	905.9	812.5	993.5	832.7	932.0	673.4	886.1	742.8	685.1	571.2	897.38	872.7
**Pharmacy and Investigation (global)**	1297.1	848.2	1401.9	881.8	1392.2	726.2	1264.7	765.3	1070.2	628.8	1283.23	904.8
Prescriptions (global)	1086.4	833.6	1174.4	863.4	1157.2	717.6	1066.1	756.5	851.1	607.6	1076.47	890.2
Investigations ^1^ (global)	210.7	125.5	227.5	136.7	235.0	141.4	198.6	116.6	219.0	124.5	206.5	122.7
**Indirect cost**												
Cost of absence from work	5857.0	4637.7	5578.1	4004.1	6704.5	5163.6	6570.1	4464.6	4623.8	4162.8	5623.6	5000.7
**Total Overall cost**	1010.6	7616.5	16,422.2	6547.9	18,457.9	9204.4	17,719.5	7573.3	15,218.9	6390.2	16,971.3	8111.1
**Major bleeding-related costs**												
**Outpatient**	741.4	265.1	745.5	256.0	760.1	304.1	705.0	245.9	814.8	268.6	753.0	275.5
GPs visits	319.4	128.7	321.8	118.2	304.4	119.3	297.2	119.6	353.7	127.5	329.7	138.2
Specialist visits	375.5	170.6	378.8	170.9	402.4	196.2	363.1	165.9	405.9	178.1	376.1	169.9
Investigations ^1^ (outpatient)	38.2	51.4	36.0	49.3	40.2	51.6	36.6	49.7	47.1	62.7	39.3	52.0
Prescriptions (outpatient)	8.4	16.2	9.0	15.2	13.0	23.5	8.11	15.8	8.1	18.6	7.9	16.0
**Inpatient—Bleeding episode (hospital + all other costs in bleeding event hospitalization)**	7648.2	2674.5	7570.4	2330.1	8517.2	4056.5	7719.2	2541.4	7269.5	2705.4	7625.1	2786.9
Hospitalizations (>24 h)	6852.8	2614.9	6658.7	2265.1	7640.2	3995.4	6933.5	2492.8	6619.0	2632.1	6874.0	2724.2
Investigations ^1^ (within hospital)	96.4	80.6	113.3	93.8	109.9	91.4	91.2	75.7	90.1	67.7	90.5	75.2
Prescriptions (within hospital)	699.0	568.0	798.5	613.7	767.2	634.6	694.5	569.5	560.4	474.4	660.6	538.4
**Pharmacy and Investigation (global)**	842.0	578.7	956.7	625.3	930.3	632.0	830.4	581.0	705.7	485.7	798.2	547.0
Prescriptions	707.4	569.2	807.5	615.9	780.2	631.1	702.6	570.8	568.6	475.7	668.5	539.0
Investigations ^1^ (global)	134.6	95.5	149.2	106.0	150.1	104.4	127.8	92.4	137.2	90.7	129.8	90.5
**Indirect cost**												
Cost of absence from work	4920.3	3315.6	4875.5	2985.5	5942.8	4533.3	5478.8	3135.8	4118.5	3463.7	4597.2	3406.8
**Total Overall bleeding cost**	13,310.0	5153.1	13,191.4	4425.8	15,220.0	8094.6	13,903.0	5145.0	12,202.8	5227.6	12,975.3	5163.6

^1^ Laboratory/radiology investigations. Quantitative variables are presented as mean plus standard deviation; qualitative variables are presented by their absolute frequencies GIB, ICH, Genitourinary, Respiratory and Other. GIB: gastrointestinal bleeding; GP: general practitioners; HCRU: healthcare resource utilization; ICH: intracranial hemorrhage; MB: major bleeding.

## Data Availability

This was a secondary data study using the BIG-PACR database, and the data can be obtained upon reasonable request.

## Supplementary Materials

The following supporting information can be downloaded at: https://www.mdpi.com/article/10.3390/jcm14041377/s1, Table S1: ICD-9 and ICD-10 bleeding codes. Table S2: Description of costs/units (2023). Table S3: Baseline clinical characteristics on the day prior to index day (day of the first major bleeding) according to the type of bleeding. Table S4: Cumulative clinical outcomes in the overall study population and according to the first major bleeding type. Table S5: Cumulative outpatient visits and hospitalization for 3 years from index date in the overall study population and according to the first major bleeding type. Table S6: Cumulative length of hospital stays, number of prescriptions and work absences for 3 years from index date in the overall study population and according to the first major bleeding type. Table S7: Cumulative costs for 3 years from index date in the overall study population and according to the first major bleeding type. Figure S1: Cumulative incidence (per 100 patient-years) curves for events for all bleedings; Figure S2: Cumulative incidence (per 100 patient-years) curves for events for gastrointestinal bleedings; Figure S3: Cumulative incidence (per 100 patient-years) curves for events for intracranial bleedings; Figure S4: Cumulative incidence (per 100 patient-years) curves for events for genitourinary bleedings; Figure S5: Cumulative incidence (per 100 patient-years) curves for events for respiratory bleedings; Figure S6: Cumulative incidence (per 100 patient-years) curves for events for other bleedings; Figure S7: Cumulative incidence (per 100 patient-years) curves for events for trauma bleedings.
