# Supplementary material for: Clinical and Economic Impact of a First Major Bleeding Event in Non-Anticoagulated Patients in Spain: A 3-Year Retrospective Observational Cohort Study
Source: J Clin Med. 2025 Feb 19;14(4):1377. doi: 10.3390/jcm14041377 (PMC11857060; doi:10.3390/jcm14041377)
Supplement: Supplementary file 1 [file jcm-14-01377-s001.zip › jcm-3475797-supplementary.pdf]

Supplementary Table S1. ICD-9 and ICD-10 bleeding codes.

ICD-9	Diagnosis	MB Classification	Subtype	Trauma
280	Anemia due to blood loss	Other life-threatening bleeding	Unspecified	
285.1	Acute posthemorrhagic anemia	Other life-threatening bleeding	Unspecified	
285.9	Anemia, unspecified	Other life-threatening bleeding	Unspecified	
430	Subarachnoid hemorrhage	ICH	SAH	
431	Intracerebral hemorrhage	ICH	ICeH	
432.1	Subdural hemorrhage	ICH	Other	
455	Hemorrhoids	GIB	Lower	
455.2	Internal hemorrhoids with other complication	GIB	Lower	
455.8	Unspecified hemorrhoids with other complication	GIB	Lower	
459	Hemorrhage, unspecified	Other life-threatening bleeding	Unspecified	
530.1	Esophagitis	GIB	Upper	
530.7	Mallory-Weiss tear	GIB	Upper	
531.0x	Acute gastric ulcer with hemorrhage	GIB	Upper	
531.4x	Chronic or unspecified gastric ulcer with hemorrhage	GIB	Upper	
532.0x	Acute duodenal ulcer with hemorrhage	GIB	Upper	
532.4x	Chronic or unspecified duodenal ulcer with hemorrhage	GIB	Upper	
533.0x	Acute peptic ulcer, site unspecified, hemorrhage	GIB	Upper	
535.01	Acute gastritis with hemorrhage	GIB	Upper	
535.11	Atrophic gastritis with hemorrhage	GIB	Upper	
535.41	Other specified gastritis with hemorrhage	GIB	Upper	
535.51	Unspecified gastritis and gastroduodenitis with hemorrhage	GIB	Upper	
535.61	Duodenitis with hemorrhage	GIB	Upper	
537.83	Angiodysplasia of stomach and duodenum with hemorrhage	GIB	Upper	
562.1	Diverticula of colon without mention of hemorrhage	GIB	Lower	
562.13	Diverticulosis of colon with hemorrhag	GIB	Lower	
562.13	Diverticulitis of colon with hemorrhage	GIB	Lower	
568.81	Hemoperitoneum	Other life-threatening bleeding	Unspecified	
569.3	Hemorrhage of rectum and anus	GIB	Lower	
569.85	Angiodysplasia of intestine with hemorrhage	GIB	Lower	
578	Hematemesis	GIB	Upper	
578.1	Blood in stool	GIB	Lower	
578.9	Hemorrhage of gastrointestinal tract, unspecified	GIB	Unspecified	
599.7	Hematuria	Other life-threatening bleeding	Genitourinary	
623.8	Other specified noninflammatory disorders of vagina	Other life-threatening bleeding	Genitourinary	
626.2	Excessive/frequent menstruation	Other life-threatening bleeding	Genitourinary	
719.1x	Hemarthrosis	Other life-threatening bleeding	Hemarthrosis	

784.7	Epistaxis	Other life-threatening bleeding	Respiratory	
796.3	Hemoptysis	Other life-threatening bleeding	Respiratory	
790.92	Abnormal coagulation profile	Other life-threatening bleeding	Other	

ICD-10	Diagnosis	MB Classification	Subtype	Trauma	Comments
D699	Hemorrhagic condition, unspecified	Other life-threatening bleeding	Unspecified		Only considered major if fatal bleeding or causing hospitalization
J942	Hemothorax	Other life-threatening bleeding	Respiratory		Only considered major if fatal bleeding or causing hospitalization
K226	Gastro-esophageal laceration-hemorrhage syndrome	GIB	Upper		Only considered major if fatal bleeding or causing hospitalization
K250	Gastric ulcer; Acute with hemorrhage	GIB	Upper		Only considered major if fatal bleeding or causing hospitalization
K252	Gastric ulcer; Acute with both hemorrhage and perforation	GIB	Upper		Only considered major if fatal bleeding or causing hospitalization
K254	Gastric ulcer; Chronic or unspecified with hemorrhage	GIB	Upper		Only considered major if fatal bleeding or causing hospitalization
K256	Gastric ulcer; Chronic or unspecified with both hemorrhage and perforation	GIB	Upper		Only considered major if fatal bleeding or causing hospitalization
K260	Duodenal ulcer; Acute with hemorrhage	GIB	Upper		Only considered major if fatal bleeding or causing hospitalization
K262	Duodenal ulcer; Acute with both hemorrhage and perforation	GIB	Upper		Only considered major if fatal bleeding or causing hospitalization
K264	Duodenal ulcer; Chronic or unspecified with hemorrhage	GIB	Upper		Only considered major if fatal bleeding or causing hospitalization
K266	Duodenal ulcer; Chronic or unspecified with both hemorrhage and perforation	GIB	Upper		Only considered major if fatal bleeding or causing hospitalization
K270	Peptic ulcer, site unspecified; Acute with hemorrhage	GIB	Upper		Only considered major if fatal bleeding or causing hospitalization
K272	Peptic ulcer, site unspecified; Acute with both hemorrhage and perforation	GIB	Upper		Only considered major if fatal bleeding or causing hospitalization
K274	Peptic ulcer, site unspecified; Chronic or unspecified with hemorrhage	GIB	Upper		Only considered major if fatal bleeding or causing hospitalization
K276	Peptic ulcer, site unspecified; Chronic or unspecified with both hemorrhage and perforation	GIB	Upper		Only considered major if fatal bleeding or causing hospitalization
K280	Gastrojejunal ulcer; Acute with hemorrhage	GIB	Upper		Only considered major if fatal bleeding or causing hospitalization
K282	Gastrojejunal ulcer; Acute with both hemorrhage and perforation	GIB	Upper		Only considered major if fatal bleeding or causing hospitalization
K284	Gastrojejunal ulcer; Chronic or unspecified with hemorrhage	GIB	Upper		Only considered major if fatal bleeding or causing hospitalization
K286	Gastrojejunal ulcer; Chronic or unspecified with both hemorrhage and perforation	GIB	Upper		Only considered major if fatal bleeding or causing hospitalization
K290	Acute hemorrhagic gastritis	GIB	Upper		Only considered major if fatal bleeding or causing hospitalization
K625	Hemorrhage of anus and rectum	GIB	Lower		Only considered major if fatal bleeding or causing hospitalization
K661	Hemoperitoneum	Other life-threatening bleeding	Unspecified		Only considered major if fatal bleeding or causing hospitalization
K920	Hematemesis	GIB	Lower		Only considered major if fatal bleeding or causing hospitalization
K921	Melena	GIB	Lower		Only considered major if fatal bleeding or causing hospitalization
K922	Gastrointestinal hemorrhage, unspecified	GIB	Unspecified		Only considered major if fatal bleeding or causing hospitalization
N020	Recurrent and persistent haematuria; Minor glomerular abnormality	Other life-threatening bleeding	Genitourinary		Only considered major if fatal bleeding or causing hospitalization
N021	Recurrent and persistent haematuria; Focal and segmental glomerular lesions	Other life-threatening bleeding	Genitourinary		Only considered major if fatal bleeding or causing hospitalization
N022	Recurrent and persistent haematuria; Diffuse membranous glomerulonephritis	Other life-threatening bleeding	Genitourinary		Only considered major if fatal bleeding or causing hospitalization

N023	Recurrent and persistent haematuria; Diffuse mesangial proliferative glomerulonephritis	Other life-threatening bleeding	Genitourinary		Only considered major if fatal bleeding or causing hospitalization
N024	Recurrent and persistent haematuria; Diffuse endocapillary proliferative glomerulonephritis	Other life-threatening bleeding	Genitourinary		Only considered major if fatal bleeding or causing hospitalization
N025	Recurrent and persistent haematuria; Diffuse mesangiocapillary glomerulonephritis	Other life-threatening bleeding	Genitourinary		Only considered major if fatal bleeding or causing hospitalization
N026	Recurrent and persistent hematuria; Dense deposit disease	Other life-threatening bleeding	Genitourinary		Only considered major if fatal bleeding or causing hospitalization
N027	Recurrent and persistent hematuria; Diffuse crescentic glomerulonephritis	Other life-threatening bleeding	Genitourinary		Only considered major if fatal bleeding or causing hospitalization
N028	Recurrent and persistent hematuria; Other	Other life-threatening bleeding	Genitourinary		Only considered major if fatal bleeding or causing hospitalization
N029	Recurrent and persistent haematuria; Unspecified	Other life-threatening bleeding	Genitourinary		Only considered major if fatal bleeding or causing hospitalization
N421	Congestion and hemorrhage of prostate	Other life-threatening bleeding	Genitourinary		Only considered major if fatal bleeding or causing hospitalization
N836	Hematosalpinx	Other life-threatening bleeding	Genitourinary		Only considered major if fatal bleeding or causing hospitalization
N837	Hematoma of broad ligament	Other life-threatening bleeding	Genitourinary		Only considered major if fatal bleeding or causing hospitalization
N857	Hematometra	Other life-threatening bleeding	Genitourinary		Only considered major if fatal bleeding or causing hospitalization
N897	Hematocolpos	Other life-threatening bleeding	Genitourinary		Only considered major if fatal bleeding or causing hospitalization
N920	Excessive and frequent menstruation with regular cycle	Other life-threatening bleeding	Genitourinary		Only considered major if fatal bleeding or causing hospitalization
N921	Excessive and frequent menstruation with irregular cycle	Other life-threatening bleeding	Genitourinary		Only considered major if fatal bleeding or causing hospitalization
N922	Excessive menstruation at puberty	Other life-threatening bleeding	Genitourinary		Only considered major if fatal bleeding or causing hospitalization
N923	Ovulation bleeding	Other life-threatening bleeding	Genitourinary		Only considered major if fatal bleeding or causing hospitalization
N924	Excessive bleeding in the premenopausal period	Other life-threatening bleeding	Genitourinary		Only considered major if fatal bleeding or causing hospitalization
N930	Postcoital and contact bleeding	Other life-threatening bleeding	Genitourinary		Only considered major if fatal bleeding or causing hospitalization
N938	Other specified abnormal uterine and vaginal bleeding	Other life-threatening bleeding	Genitourinary		Only considered major if fatal bleeding or causing hospitalization
N939	Abnormal uterine and vaginal bleeding, unspecified	Other life-threatening bleeding	Genitourinary		Only considered major if fatal bleeding or causing hospitalization
N950	Postmenopausal bleeding	Other life-threatening bleeding	Genitourinary		Only considered major if fatal bleeding or causing hospitalization
R040	Epistaxis	Other life-threatening bleeding	Respiratory		Only considered major if fatal bleeding or causing hospitalization
R041	Hemorrhage from throat	Other life-threatening bleeding	Respiratory		Only considered major if fatal bleeding or causing hospitalization
R042	Hemoptysis	Other life-threatening bleeding	Respiratory		Only considered major if fatal bleeding or causing hospitalization
R048	Hemorrhage from other sites in respiratory passages	Other life-threatening bleeding	Respiratory		Only considered major if fatal bleeding or causing hospitalization
R049	Hemorrhage from respiratory passages, unspecified	Other life-threatening bleeding	Respiratory		Only considered major if fatal bleeding or causing hospitalization
R31	Unspecified hematuria	Other life-threatening bleeding	Genitourinary		Only considered major if fatal bleeding or causing hospitalization
R58	Hemorrhage, not elsewhere classified	Other life-threatening bleeding	Unspecified		Only considered major if fatal bleeding or causing hospitalization
S271	Traumatic hemothorax	Other life-threatening bleeding	Respiratory	Trauma	Only considered major if fatal bleeding or causing hospitalization
T792	Traumatic secondary and recurrent hemorrhage	Other life-threatening bleeding	Unspecified	Trauma	Only considered major if fatal bleeding or causing hospitalization
T810	Hemorrhage and hematoma complicating a procedure, not elsewhere classified	Other life-threatening bleeding	Unspecified		Only considered major if fatal bleeding or causing hospitalization
	Critical site: always major: all lines below, including intraocular				
I230	Hemopericardium as current complication following acute myocardial infarction	Other life-threatening bleeding	Hemopericardium		Always considered major
I312	Hemopericardium, not elsewhere classified	Other life-threatening bleeding	Hemopericardium		Always considered major

I600	Subarachnoid hemorrhage from carotid siphon and bifurcation	ICH	SAH		Always considered major
I601	Subarachnoid hemorrhage from middle cerebral artery	ICH	SAH		Always considered major
I602	Subarachnoid hemorrhage from anterior communicating artery	ICH	SAH		Always considered major
I603	Subarachnoid hemorrhage from posterior communicating artery	ICH	SAH		Always considered major
I604	Subarachnoid hemorrhage from basilar artery	ICH	SAH		Always considered major
I605	Subarachnoid hemorrhage from vertebral artery	ICH	SAH		Always considered major
I606	Subarachnoid hemorrhage from other intracranial arteries	ICH	SAH		Always considered major
I607	Subarachnoid hemorrhage from intracranial artery, unspecified	ICH	SAH		Always considered major
I608	Other subarachnoid hemorrhage	ICH	SAH		Always considered major
I609	Subarachnoid hemorrhage, unspecified	ICH	SAH		Always considered major
I610	Intracerebral hemorrhage in hemisphere, subcortical	ICH	ICeH		ALWAYS CONSIDERED MAJOR
I611	Intracerebral hemorrhage in hemisphere, cortical	ICH	ICeH		Always considered major
I612	Intracerebral hemorrhage in hemisphere, unspecified	ICH	ICeH		Always considered major
I613	Intracerebral hemorrhage in brain stem	ICH	ICeH		Always considered major
I614	Intracerebral hemorrhage in cerebellum	ICH	ICeH		Always considered major
I615	Intracerebral hemorrhage, intraventricular	ICH	ICeH		Always considered major
I616	Intracerebral hemorrhage, multiple localized	ICH	ICeH		Always considered major
I618	Other intracerebral hemorrhage	ICH	ICeH		Always considered major
I619	Intracerebral hemorrhage, unspecified	ICH	ICeH		Always considered major
I620	Subdural hemorrhage (acute)(nontraumatic)	ICH	Other ICH		Always considered major
I621	Nontraumatic extradural hemorrhage	ICH	Other ICH		Always considered major
I629	Intracranial hemorrhage (nontraumatic), unspecified	ICH	Other ICH		Always considered major
I713	Abdominal aortic aneurysm, ruptured	Other life-threatening bleeding	Retroperitoneal		Always considered major
I715	Thoracoabdominal aortic aneurysm, ruptured	Other life-threatening bleeding	Unspecified		Always considered major
I718	Aortic aneurysm of unspecified site, ruptured	Other life-threatening bleeding	Unspecified		Always considered major
M250	Hemarthrosis	Other life-threatening bleeding	Hemarthrosis		Always considered major
P544	Neonatal adrenal hemorrhage	Other life-threatening bleeding	Retroperitoneal		Always considered major
S064	Epidural hemorrhage	ICH	Other ICH		Always considered major
S065	Traumatic subdural hemorrhage	ICH	Other ICH	Trauma	Always considered major
S066	Traumatic subarachnoid hemorrhage	ICH	SAH	Trauma	Always considered major
	Critical intra-ocular tab: 4 lines below	Other life-threatening bleeding	Unspecified		Always considered major
H313	Choroidal hemorrhage and rupture	Other life-threatening bleeding	Unspecified		Always considered major
H356	Retinal hemorrhage	Other life-threatening bleeding	Unspecified		Always considered major
H431	Vitreous hemorrhage	Other life-threatening bleeding	Unspecified		Always considered major
H450	Vitreous hemorrhage in diseases classified elsewhere	Other life-threatening bleeding	Unspecified		Always considered major
	Anemia: 2 lines below – If in temporal relationship with a MB location code, location code used				
D500	Iron deficiency anemia secondary to blood loss (chronic)	Other life-threatening bleeding	Unspecified		Always considered major
D62	Acute posthemorrhagic anemia	Other life-threatening bleeding	Unspecified		Always considered major

*Major bleeding: all critical site bleeding codes below and anemia codes below. Other bleeding codes: only major if they are fatal or cause hospitalization. GIB: gastrointestinal bleeding; ICeH: Intracerebral hemorrhage; ICH: intracranial bleeding; MB: major bleeding; SAH: subarachnoid hemorrhage.

Supplementary Table S2. Description of costs/units (2023).

**Outpatient visits:**	
GP visit	64.12
Specialist visit*	236.59
Hospital (stay / day)	717.68
**Investigations:**	
Laboratory tests	56.76
Conventional radiology	46.15
Computed tomography	212.29
Magnetic nuclear resonance	356.32
**Indirect costs:**	
Cost per day not worked	274.05
Pharmaceutical prescription	RP+VAT
*Cardiologists, Internal Medicine Physicians, Neurologists, Gastroenterologists, Intensivists, Oncologists, Orthopedic Surgeons	

Cost data was inferred from the eSalud Consulting database (Database of Spanish healthcare costs and cost-effectiveness ratios: eSalud [Internet]. Barcelona: Oblikue Consulting, S.L; 2007 [last update: 2018; consultation date: 15/02/2024]. Available at: http://esalud.oblikue.com/). Pharmaceutical prescription costs were based on the full price of product (BOTPLUS Database General Council of Pharmacist Colleges. Available at: https://botplusweb.farmaceuticos.com/). Costs of absence from work were calculated by multiplying the number of days of absence from work due to sickness by the mean daily salary of a working person in Spain (available at https://www.ine.es/dynt3/inebase/index.htm?padre=4563&capsel=4563; Accessed: 15/02/2024).

Supplementary Table S3. Baseline clinical characteristics on the day prior to index day (day of the first major bleeding) according to the type of bleeding.

	GIB (N=917)	Lower (N=205)	Upper (N=566)	Other GIB (N=146)	ICH (N=127)	SAH (N=64)	ICeH (N=52)	Other ICH (N=11)	Trauma (N=62)
**Biodemographic data**									
Age, years (SD)	57.59 (11.33)	55.4 (12.21)	58.34 (10.57)	57.74 (12.53)	55.73 (11.7)	54.24 (11.11)	57.01 (11.46)	58.34 (15.74)	57.88 (16.57)
<45 years, n (%)	95 (10.36)	35 (17.07)	35 (6.18)	25 (17.12)	20 (15.75)	13 (20.31)	6 (11.54)	1 (9.09)	11 (17.74)
45-64 years, n (%)	627 (68.38)	136 (66.34)	407 (71.91)	84 (57.53)	82 (64.57)	42 (65.63)	33 (63.46)	7 (63.64)	33 (53.23)
65-74 years, n (%)	114 (12.43)	15 (7.32)	74 (13.07)	25 (17.12)	16 (12.6)	5 (7.81)	11 (21.15)	0 (0)	2 (3.23)
75-84 years, n (%)	57 (6.22)	12 (5.85)	38 (6.71)	7 (4.79)	6 (4.72)	3 (4.69)	0 (0)	3 (27.27)	11 (17.74)
≥85 years, n (%)	24 (2.62)	7 (3.41)	12 (2.12)	5 (3.42)	3 (2.36)	1 (1.56)	2 (3.85)	0 (0)	5 (8.06)
Sex (female), n (%)	331 (36.1)	64 (31.22)	216 (38.16)	51 (34.93)	42 (33.07)	20 (31.25)	19 (36.54)	3 (27.27)	25 (40.32)
BMI, Kg/m2 (SD)	28.89 (16.25)	28.04 (13.92)	29.06 (17.01)	28.95 (14.95)	28.35 (14.69)	29.02 (14.81)	27.28 (14.58)	29.64 (15.93)	28.16 (14.76)
Alcohol use, n (%)	25 (2.73)	9 (4.39)	13 (2.3)	3 (2.05)	4 (3.15)	1 (1.56)	3 (5.77)	0 (0)	1 (1.61)
Updated Charlson comorbidity index	0.5 (0.81)	1.21 (0.76)	0.21 (0.63)	0.62 (0.84)	1.16 (0.48)	1.11 (0.36)	1.19 (0.56)	1.27 (0.65)	0.52 (0.7)
**Cardiovascular risk factors**									
Hypertension, n (%)	82 (8.94)	12 (5.85)	52 (9.19)	18 (12.33)	24 (18.9)	12 (18.75)	10 (19.23)	2 (18.18)	8 (12.9)
Hypercholesterolemia, n (%)	127 (13.85)	14 (6.83)	88 (15.55)	25 (17.12)	9 (7.09)	4 (6.25)	5 (9.62)	0 (0)	7 (11.29)
Diabetes type 2, n (%)	28 (3.05)	5 (2.44)	19 (3.36)	4 (2.74)	4 (3.15)	1 (1.56)	2 (3.85)	1 (9.09)	6 (9.68)
Diabetes type 1, n (%)	2 (0.22)	0 (0)	1 (0.18)	1 (0.68)	1 (0.79)	0 (0)	1 (1.92)	0 (0)	0 (0)
Smoking, n (%)	37 (4.03)	9 (4.39)	22 (3.89)	6 (4.11)	4 (3.15)	4 (6.25)	0 (0)	0 (0)	2 (3.23)
**Vascular disease**									
Heart failure, n (%)	114 (12.43)	16 (7.8)	81 (14.31)	17 (11.64)	14 (11.02)	6 (9.38)	7 (13.46)	1 (9.09)	6 (9.68)
Chronic kidney disease, n (%)	2 (0.22)	0 (0)	2 (0.35)	0 (0)	1 (0.79)	1 (1.56)	0 (0)	0 (0)	1 (1.61)
Myocardial infarction, n (%)	32 (3.49)	6 (2.93)	25 (4.42)	1 (0.68)	6 (4.72)	3 (4.69)	2 (3.85)	1 (9.09)	1 (1.61)
Stroke, n (%)	1 (0.11)	0 (0)	1 (0.18)	0 (0)	52 (40.94)	32 (50)	16 (30.77)	4 (36.36)	2 (3.23)
Peripheral vascular disease, n (%)	3 (0.33)	2 (0.98)	1 (0.18)	0 (0)	1 (0.79)	0 (0)	0 (0)	1 (9.09)	0 (0)
Venous thromboembolism, n (%)	1 (0.11%)	0 (0%)	1 (0.18%)	0 (0%)	0 (0%)	0 (0%)	0 (0%)	0 (0%)	0 (0%)
Atrial fibrillation, n (%)	15 (1.64%)	3 (1.46%)	11 (1.94%)	1 (0.68%)	3 (2.36%)	0 (0%)	3 (5.77%)	0 (0%)	1 (1.61%)
Non-mechanical cardiac-valve replacement, n (%)	1 (0.11%)	0 (0%)	1 (0.18%)	0 (0%)	0 (0%)	0 (0%)	0 (0%)	0 (0%)	0 (0%)
**Other comorbidities**									
Anemia, n (%)	43 (4.69)	10 (4.88)	26 (4.59)	7 (4.79)	3 (2.36)	1 (1.56)	2 (3.85)	0 (0)	31 (50)
Any malignancy, n % (%)	106 (11.56)	26 (12.68)	61 (10.78)	19 (13.01)	13 (10.24)	5 (7.81)	7 (13.46)	1 (9.09)	8 (12.9)
Chronic pulmonary disease, n (%)	69 (7.52)	12 (5.85)	38 (6.71)	19 (13.01)	6 (4.72)	4 (6.25)	1 (1.92)	1 (9.09)	4 (6.45)
Peptic ulcer disease, n (%)	262 (28.57)	204 (99.51)	8 (1.41)	50 (34.25)	0 (0)	0 (0)	0 (0)	0 (0)	0 (0)
Dementia, n (%)	2 (0.22)	2 (0.98)	0 (0)	0 (0)	1 (0.79)	0 (0)	1 (1.92)	0 (0)	0 (0)

Moderate/severe liver disease, n (%)	4 (0.44)	1 (0.49)	3 (0.53)	0 (0)	0 (0)	0 (0)	0 (0)	0 (0)	0 (0)
**Biochemical parameters**									
Hemoglobin, g/dL (SD)	14.65 (7.32)	14.11 (6.06)	14.71 (7.45)	14.80 (7.3)	14.72 (5.82)	14.84 (6.22)	14.49 (5.03)	14.7 (6.9)	12.29 (6.36)
HbA1c, % (SD)	5.75 (1.81)	5.65 (1.1)	5.76 (1.95)	5.77 (1.97)	6.75 (2.03)	7.10 (1.78)	6.48 (1.98)	6.71 (3.14)	7.06 (3.02)
Platelet count, ×103/μL (SD)	249.28 (129.55)	259.87 (118.02)	249.06 (130.31)	241.18 (126.04)	236.71 (97.2)	238 (105.11)	228.86 (80.89)	249 (118.88)	299 (165.96)
eGFR, mL/min/1.73 m^2 (SD)	122.85 (43.05)	103.42 (26.91)	127.03 (47.54)	117.54 (42.1)	128.44 (34.83)	125.96 (28.64)	151.28 (43.88)	44.5 (13.42)	87.24 (31.31)
Creatinine clearance, mL/min (SD)	90.98 (39)	95.47 (31.17)	90.5 (41.29)	89.93 (37.17)	90.08 (32.72)	93.15 (34.53)	87.29 (30.62)	84.5 (34.19)	96.08 (38.82)
**Concomitant treatments (within 120 days prior to index)**									
NSAIDs, n (%)	39 (4.25)	3 (1.46)	28 (4.95)	8 (5.48)	2 (1.57)	2 (3.13)	0 (0)	0 (0)	2 (3.23)
Prior AC (ended >60 days prior to index), n (%)	33 (3.6)	5 (2.44)	22 (3.89)	6 (4.11)	8 (6.3)	3 (4.69)	2 (3.85)	3 (27.27)	1 (1.61)
Antidiabetic drugs, n (%)	30 (3.27)	5 (2.44)	20 (3.53)	5 (3.42)	5 (3.94)	1 (1.56)	3 (5.77)	1 (9.09)	6 (9.68)
Gastroprotective agents, n (%)	34 (3.71)	7 (3.41)	16 (2.83)	11 (7.53)	6 (4.72)	1 (1.56)	4 (7.69)	1 (9.09)	2 (3.23)
Antiplatelet drugs, n (%)	26 (2.84)	9 (4.39)	15 (2.65)	2 (1.37)	4 (3.15)	0 (0)	3 (5.77)	1 (9.09)	2 (3.23)
Antihypertensive therapies, n (%)	23 (2.51)	3 (1.46)	14 (2.47)	6 (4.11)	6 (4.72)	3 (4.69)	2 (3.85)	1 (9.09)	2 (3.23)
Lipid lowering therapies, n (%)	24 (2.62)	1 (0.49)	17 (3)	6 (4.11)	2 (1.57)	0 (0)	1 (1.92)	1 (9.09)	1 (1.61)

Quantitative variables are presented as mean and (standard deviation); qualitative variables are presented as absolute and relative (%) frequencies. GIB, ICH, Genitourinary, Respiratory and Other. AC: anticoagulant; BMI: body mass index; eGFR: estimated glomerular filtration rate; GIB: gastrointestinal bleeding; ICH: Intracranial hemorrhage; ICeH: intracerebral hemorrhage; MB: major bleeding; NSAIDs: non-steroidal anti-inflammatory drugs; SAH: subarachnoid hemorrhage.

Supplementary Table S4. Cumulative clinical outcomes in the overall study population and according to the first major bleeding type.

Time window[1]	Overall (N= 4089)			GIB (N=917)			Lower GIB (N= 205)			Upper GIB (N= 566)			Other GIB (N= 146)		
	n	%	Rates (95% CI)	n	%	Rates (95% CI)	n	%	Rates (95% CI)	n	%	Rates (95% CI)	n	%	Rates (95% CI)
**Incidence Rates[2]**															
**3 months since index date**															
Death from any cause	75	1.83%	7.51 (6.7 - 8.32)	17	1.85%	7.58 (5.87 - 9.29)	4	1.95%	7.96 (4.25 - 11.67)	11	1.94%	7.96 (5.73 - 10.19)	2	1.37%	5.57 (1.85 - 9.29)
Cardiovascular death	18	0.44%	1.8 (1.39 - 2.21)	4	0.44%	1.78 (0.92 - 2.64)	0	0.00%	0 (0 - 0)	4	0.71%	2.9 (1.52 - 4.28)	0	0.00%	0 (0 - 0)
AMI	45	1.10%	4.53 (3.89 - 5.17)	9	0.98%	4.03 (2.76 - 5.3)	2	0.98%	4.01 (1.32 - 6.7)	6	1.06%	4.36 (2.68 - 6.04)	1	0.68%	2.79 (0.12 - 5.46)
Ischemic stroke	35	0.86%	3.52 (2.96 - 4.08)	10	1.09%	4.48 (3.14 - 5.82)	1	0.49%	2 (0.08 - 3.92)	5	0.88%	3.64 (2.1 - 5.18)	4	2.74%	11.26 (6.13 - 16.39)
Acute kidney failure	1	0.02%	0.1 (0 - 0.2)	0	0.00%	0 (0 - 0)	0	0.00%	0 (0 - 0)	0	0.00%	0 (0 - 0)	0	0.00%	0 (0 - 0)
Acute liver failure	5	0.12%	0.5 (0.28 - 0.72)	0	0.00%	0 (0 - 0)	0	0.00%	0 (0 - 0)	0	0.00%	0 (0 - 0)	0	0.00%	0 (0 - 0)
**6 months since index date**															
Death from any cause	128	3.13%	6.36 (5.61 - 7.11)	30	3.27%	6.65 (5.04 - 8.26)	5	2.44%	4.94 (1.97 - 7.91)	18	3.18%	6.47 (4.44 - 8.5)	7	4.79%	9.77 (4.95 - 14.59)
Cardiovascular death	29	0.71%	1.44 (1.07 - 1.81)	7	0.76%	1.55 (0.75 - 2.35)	0	0.00%	0 (0 - 0)	6	1.06%	2.16 (0.96 - 3.36)	1	0.68%	1.4 (0 - 3.31)
AMI	73	1.79%	3.65 (3.08 - 4.22)	17	1.85%	3.79 (2.55 - 5.03)	4	1.95%	3.98 (1.3 - 6.66)	9	1.59%	3.25 (1.79 - 4.71)	4	2.74%	5.62 (1.88 - 9.36)
Ischemic stroke	62	1.52%	3.10 (2.57 - 3.63)	16	1.74%	3.57 (2.37 - 4.77)	4	1.95%	3.99 (1.31 - 6.67)	8	1.41%	2.89 (1.51 - 4.27)	4	2.74%	5.64 (1.9 - 9.38)
Acute kidney failure	2	0.05%	0.1 (0 - 0.2)	0	0.00%	0 (0 - 0)	0	0.00%	0 (0 - 0)	0	0.00%	0 (0 - 0)	0	0.00%	0 (0 - 0)
Acute liver failure	8	0.20%	0.4 (0.21 - 0.59)	0	0.00%	0 (0 - 0)	0	0.00%	0 (0 - 0)	0	0.00%	0 (0 - 0)	0	0.00%	0 (0 - 0)
**12 months since index date**															
Death from any cause	172	4.21%	4.33 (3.71 - 4.95)	42	4.58%	4.71 (3.34 - 6.08)	10	4.88%	5.00 (2.02 - 7.98)	23	4.06%	4.18 (2.53 - 5.83)	9	6.16%	6.39 (2.42 - 10.36)
Cardiovascular death	44	1.08%	1.11 (0.79 - 1.43)	10	1.09%	1.12 (0.44 - 1.8)	1	0.49%	0.50 (0 - 1.47)	8	1.41%	1.45 (0.47 - 2.43)	1	0.68%	0.71 (0 - 2.07)
AMI	107	2.62%	2.72 (2.22 - 3.22)	26	2.84%	2.95 (1.85 - 4.05)	6	2.93%	3.03 (0.68 - 5.38)	15	2.65%	2.75 (1.4 - 4.1)	5	3.42%	3.59 (0.57 - 6.61)
Ischemic stroke	94	2.30%	2.39 (1.92 - 2.86)	21	2.29%	2.38 (1.39 - 3.37)	5	2.44%	2.53 (0.38 - 4.68)	11	1.94%	2.02 (0.86 - 3.18)	5	3.42%	3.58 (0.57 - 6.59)
Acute kidney failure	2	0.05%	0.05 (0 - 0.12)	0	0.00%	0 (0 - 0)	0	0.00%	0 (0 - 0)	0	0.00%	0 (0 - 0)	0	0.00%	0 (0 - 0)
Acute liver failure	13	0.32%	0.33 (0.15 - 0.51)	0	0.00%	0 (0 - 0)	0	0.00%	0 (0 - 0)	0	0.00%	0 (0 - 0)	0	0.00%	0 (0 - 0)
**2 years since index date**															
Death from any cause	239	5.84%	3.04 (2.51 - 3.57)	59	6.43%	3.36 (2.19 - 4.53)	11	5.37%	2.80 (0.54 - 5.06)	37	6.54%	3.41 (1.91 - 4.91)	11	7.53%	3.96 (0.8 - 7.12)
Cardiovascular death	67	1.64%	0.85 (0.57 - 1.13)	14	1.53%	0.80 (0.22 - 1.38)	2	0.98%	0.51 (0 - 1.49)	11	1.94%	1.01 (0.19 - 1.83)	1	0.68%	0.36 (0 - 1.33)
AMI	187	4.57%	2.42 (1.95 - 2.89)	47	5.13%	2.73 (1.68 - 3.78)	9	4.39%	2.32 (0.26 - 4.38)	31	5.48%	2.92 (1.53 - 4.31)	7	4.79%	2.56 (0 - 5.12)
Ischemic stroke	165	4.04%	2.13 (1.69 - 2.57)	34	3.71%	1.96 (1.06 - 2.86)	7	3.41%	1.80 (0 - 3.62)	19	3.36%	1.77 (0.68 - 2.86)	8	5.48%	2.91 (0.18 - 5.64)
Acute kidney failure	3	0.07%	0.04 (0 - 0.1)	1	0.11%	0.06 (0 - 0.22)	0	0.00%	0 (0 - 0)	1	0.18%	0.09 (0 - 0.34)	0	0.00%	0 (0 - 0)
Acute liver failure	16	0.39%	0.20 (0.06 - 0.34)	0	0.00%	0 (0 - 0)	0	0.00%	0 (0 - 0)	0	0.00%	0 (0 - 0)	0	0.00%	0 (0 - 0)
**3 years since index date**															
Death from any cause	291	7.12%	2.49 (2.01 - 2.97)	71	7.74%	2.72 (1.67 - 3.77)	15	7.32%	2.57 (0.4 - 4.74)	43	7.60%	2.67 (1.34 - 4)	13	8.90%	3.16 (0.32 - 6)
Cardiovascular death	82	2.01%	0.70 (0.44 - 0.96)	20	2.18%	0.77 (0.2 - 1.34)	5	2.44%	0.86 (0 - 2.12)	14	2.47%	0.87 (0.1 - 1.64)	1	0.68%	0.24 (0 - 1.03)
AMI	248	6.07%	2.18 (1.73 - 2.63)	59	6.43%	2.32 (1.35 - 3.29)	13	6.34%	2.28 (0.24 - 4.32)	38	6.71%	2.43 (1.16 - 3.7)	8	5.48%	1.97 (0 - 4.22)
Ischemic stroke	202	4.94%	1.77 (1.37 - 2.17)	40	4.36%	1.56 (0.76 - 2.36)	8	3.90%	1.39 (0 - 2.99)	22	3.89%	1.40 (0.43 - 2.37)	10	6.85%	2.47 (0 - 4.99)
Acute kidney failure	4	0.10%	0.03 (0 - 0.08)	1	0.11%	0.04 (0 - 0.17)	0	0.00%	0 (0 - 0)	1	0.18%	0.06 (0 - 0.26)	0	0.00%	0 (0 - 0)
Acute liver failure	27	0.66%	0.23 (0.08 - 0.38)	0	0.00%	0 (0 - 0)	0	0.00%	0 (0 - 0)	0	0.00%	0 (0 - 0)	0	0.00%	0 (0 - 0)
**Event Rates[3]**															
**3 months since index date**															
AMI	45	1.10%	5.11 (4.44 - 5.78)	9	0.98%	4.9 (3.5 - 6.3)	2	0.98%	5.97 (2.73 - 9.21)	6	1.06%	5.07 (3.26 - 6.88)	1	0.68%	2.79 (0.12 - 5.46)
Ischemic stroke	35	0.86%	3.81 (3.22 - 4.4)	10	1.09%	4.9 (3.5 - 6.3)	1	0.49%	1.99 (0.08 - 3.9)	5	0.88%	4.34 (2.66 - 6.02)	4	2.74%	11.15 (6.04 - 16.26)
Acute kidney failure	1	0.02%	0.20 (0.06 - 0.34)	0	0.00%	0 (0 - 0)	0	0.00%	0 (0 - 0)	0	0.00%	0 (0 - 0)	0	0.00%	0 (0 - 0)
Acute liver failure	5	0.12%	0.50 (0.28 - 0.72)	0	0.00%	0 (0 - 0)	0	0.00%	0 (0 - 0)	0	0.00%	0 (0 - 0)	0	0.00%	0 (0 - 0)
**6 months since index date**															
AMI	73	1.79%	4.67 (4.02 - 5.32)	17	1.85%	4.88 (3.49 - 6.27)	4	1.95%	5.93 (2.7 - 9.16)	9	1.59%	3.95 (2.35 - 5.55)	4	2.74%	6.98 (2.85 - 11.11)
Ischemic stroke	62	1.52%	3.92 (3.33 - 4.51)	16	1.74%	4.21 (2.91 - 5.51)	4	1.95%	3.95 (1.28 - 6.62)	8	1.41%	3.23 (1.77 - 4.69)	4	2.74%	8.37 (3.88 - 12.86)
Acute kidney failure	2	0.05%	0.15 (0.03 - 0.27)	0	0.00%	0 (0 - 0)	0	0.00%	0 (0 - 0)	0	0.00%	0 (0 - 0)	0	0.00%	0 (0 - 0)

Acute liver failure	8	0.20%	0.45 (0.24 - 0.66)	0	0.00%	0 (0 - 0)	0	0.00%	0 (0 - 0)	0	0.00%	0 (0 - 0)	0	0.00%	0 (0 - 0)
**12 months since index date**															
AMI	107	2.62%	3.60 (3.03 - 4.17)	26	2.84%	4.04 (2.77 - 5.31)	6	2.93%	5.00 (2.02 - 7.98)	15	2.65%	3.27 (1.8 - 4.74)	5	3.42%	5.68 (1.93 - 9.43)
Ischemic stroke	94	2.30%	3.12 (2.59 - 3.65)	21	2.29%	2.81 (1.74 - 3.88)	5	2.44%	2.50 (0.36 - 4.64)	11	1.94%	2.18 (0.98 - 3.38)	5	3.42%	5.68 (1.93 - 9.43)
Acute kidney failure	2	0.05%	0.08 (0 - 0.17)	0	0.00%	0 (0 - 0)	0	0.00%	0 (0 - 0)	0	0.00%	0 (0 - 0)	0	0.00%	0 (0 - 0)
Acute liver failure	13	0.32%	0.38 (0.19 - 0.57)	0	0.00%	0 (0 - 0)	0	0.00%	0 (0 - 0)	0	0.00%	0 (0 - 0)	0	0.00%	0 (0 - 0)
**2 years since index date**															
AMI	187	4.57%	3.2 (2.66 - 3.74)	47	5.13%	3.76 (2.53 - 4.99)	9	4.39%	3.3 (0.85 - 5.75)	31	5.48%	3.87 (2.28 - 5.46)	7	4.79%	3.96 (0.8 - 7.12)
Ischemic stroke	165	4.04%	2.74 (2.24 - 3.24)	34	3.71%	2.22 (1.27 - 3.17)	7	3.41%	1.78 (0 - 3.59)	19	3.36%	1.93 (0.8 - 3.06)	8	5.48%	3.96 (0.8 - 7.12)
Acute kidney failure	3	0.07%	0.05 (0 - 0.12)	1	0.11%	0.06 (0 - 0.22)	0	0.00%	0 (0 - 0)	1	0.18%	0.09 (0 - 0.34)	0	0.00%	0 (0 - 0)
Acute liver failure	16	0.39%	0.25 (0.1 - 0.4)	0	0.00%	0 (0 - 0)	0	0.00%	0 (0 - 0)	0	0.00%	0 (0 - 0)	0	0.00%	0 (0 - 0)
**3 years since index date**															
AMI	248	6.07%	2.81 (2.3 - 3.32)	59	6.43%	3.03 (1.92 - 4.14)	13	6.34%	3.08 (0.71 - 5.45)	38	6.71%	3.04 (1.63 - 4.45)	8	5.48%	2.92 (0.19 - 5.65)
Ischemic stroke	202	4.94%	2.26 (1.8 - 2.72)	40	4.36%	1.80 (0.94 - 2.66)	8	3.90%	1.37 (0 - 2.96)	22	3.89%	1.55 (0.53 - 2.57)	10	6.85%	3.4 (0.46 - 6.34)
Acute kidney failure	4	0.10%	0.04 (0 - 0.1)	1	0.11%	0.04 (0 - 0.17)	0	0.00%	0 (0 - 0)	1	0.18%	0.06 (0 - 0.26)	0	0.00%	0 (0 - 0)
Acute liver failure	27	0.66%	0.27 (0.11 - 0.43)	0	0.00%	0 (0 - 0)	0	0.00%	0 (0 - 0)	0	0.00%	0 (0 - 0)	0	0.00%	0 (0 - 0)

Time window[1]	ICH (N= 127)			SAH (N= 64)			ICeH (N=52)			Other ICH (N=11)		
	n	%	Rates (95% CI)	n	%	Rates (95% CI)	n	%	Rates (95% CI)	n	%	Rates (95% CI)
**Incidence Rates[2]**												
**3 months since index date**												
Death from any cause	12	9.45%	41.06 (32.5 - 49.62)	7	10.94%	48.26 (36.02 - 60.5)	5	9.62%	41.63 (28.23 - 55.03)	0	0.00%	0 (0 - 0)
Cardiovascular death	2	1.57%	6.84 (2.45 - 11.23)	2	3.13%	13.79 (5.34 - 22.24)	0	0.00%	0 (0 - 0)	0	0.00%	0 (0 - 0)
AMI	9	7.09%	31.42 (23.35 - 39.49)	4	6.25%	28.17 (17.15 - 39.19)	5	9.62%	42.62 (29.18 - 56.06)	0	0.00%	0 (0 - 0)
Ischemic stroke	3	2.36%	10.27 (4.99 - 15.55)	2	3.13%	13.8 (5.35 - 22.25)	1	1.92%	8.33 (0.82 - 15.84)	0	0.00%	0 (0 - 0)
Acute kidney failure	0	0.00%	0 (0 - 0)	0	0.00%	0 (0 - 0)	0	0.00%	0 (0 - 0)	0	0.00%	0 (0 - 0)
Acute liver failure	0	0.00%	0 (0 - 0)	0	0.00%	0 (0 - 0)	0	0.00%	0 (0 - 0)	0	0.00%	0 (0 - 0)
**6 months since index date**												
Death from any cause	12	9.45%	20.50 (13.48 - 27.52)	7	10.94%	24.12 (13.64 - 34.6)	5	9.62%	20.85 (9.81 - 31.89)	0	0.00%	0 (0 - 0)
Cardiovascular death	2	1.57%	3.42 (0.26 - 6.58)	2	3.13%	6.89 (0.68 - 13.1)	0	0.00%	0 (0 - 0)	0	0.00%	0 (0 - 0)
AMI	10	7.87%	17.48 (10.87 - 24.09)	5	7.81%	17.55 (8.23 - 26.87)	5	9.62%	21.55 (10.37 - 32.73)	0	0.00%	0 (0 - 0)
Ischemic stroke	4	3.15%	6.84 (2.45 - 11.23)	2	3.13%	6.89 (0.68 - 13.1)	2	3.85%	8.34 (0.83 - 15.85)	0	0.00%	0 (0 - 0)
Acute kidney failure	0	0.00%	0 (0 - 0)	0	0.00%	0 (0 - 0)	0	0.00%	0 (0 - 0)	0	0.00%	0 (0 - 0)
Acute liver failure	0	0.00%	0 (0 - 0)	0	0.00%	0 (0 - 0)	0	0.00%	0 (0 - 0)	0	0.00%	0 (0 - 0)
**12 months since index date**												
Death from any cause	15	11.81%	13.02 (7.17 - 18.87)	7	10.94%	12.18 (4.17 - 20.19)	7	13.46%	14.93 (5.24 - 24.62)	1	9.09%	9.22 (0 - 26.32)
Cardiovascular death	2	1.57%	1.74 (0 - 4.01)	2	3.13%	3.48 (0 - 7.97)	0	0.00%	0 (0 - 0)	0	0.00%	0 (0 - 0)
AMI	12	9.45%	10.73 (5.35 - 16.11)	5	7.81%	8.86 (1.9 - 15.82)	7	13.46%	15.7 (5.81 - 25.59)	0	0.00%	0 (0 - 0)
Ischemic stroke	5	3.94%	4.36 (0.81 - 7.91)	3	4.69%	5.23 (0 - 10.68)	2	3.85%	4.31 (0 - 9.83)	0	0.00%	0 (0 - 0)
Acute kidney failure	0	0.00%	0 (0 - 0)	0	0.00%	0 (0 - 0)	0	0.00%	0 (0 - 0)	0	0.00%	0 (0 - 0)
Acute liver failure	1	0.79%	0.87 (0 - 2.49)	0	0.00%	0 (0 - 0)	1	1.92%	2.15 (0 - 6.09)	0	0.00%	0 (0 - 0)
**2 years since index date**												
Death from any cause	17	13.39%	7.54 (2.95 - 12.13)	8	12.50%	7.05 (0.78 - 13.32)	8	15.38%	8.78 (1.09 - 16.47)	1	9.09%	4.8 (0 - 17.43)
Cardiovascular death	2	1.57%	0.89 (0 - 2.52)	2	3.13%	1.76 (0 - 4.98)	0	0.00%	0 (0 - 0)	0	0.00%	0 (0 - 0)
AMI	12	9.45%	5.51 (1.54 - 9.48)	5	7.81%	4.48 (0 - 9.55)	7	13.46%	8.18 (0.73 - 15.63)	0	0.00%	0 (0 - 0)
Ischemic stroke	7	5.51%	3.16 (0.12 - 6.2)	4	6.25%	3.57 (0 - 8.12)	3	5.77%	3.37 (0 - 8.27)	0	0.00%	0 (0 - 0)
Acute kidney failure	0	0.00%	0 (0 - 0)	0	0.00%	0 (0 - 0)	0	0.00%	0 (0 - 0)	0	0.00%	0 (0 - 0)
Acute liver failure	1	0.79%	0.44 (0 - 1.59)	0	0.00%	0 (0 - 0)	1	1.92%	1.1 (0 - 3.93)	0	0.00%	0 (0 - 0)
**3 years since index date**												
Death from any cause	20	15.75%	6.00 (1.87 - 10.13)	9	14.06%	5.34 (0 - 10.85)	10	19.23%	7.45 (0.31 - 14.59)	1	9.09%	3.24 (0 - 13.7)
Cardiovascular death	2	1.57%	0.60 (0 - 1.94)	2	3.13%	1.19 (0 - 3.85)	0	0.00%	0 (0 - 0)	0	0.00%	0 (0 - 0)
AMI	14	11.02%	4.36 (0.81 - 7.91)	6	9.38%	3.64 (0 - 8.23)	8	15.38%	6.36 (0 - 12.99)	0	0.00%	0 (0 - 0)
Ischemic stroke	8	6.30%	2.46 (0 - 5.15)	4	6.25%	2.42 (0 - 6.18)	3	5.77%	2.31 (0 - 6.39)	1	9.09%	3.3 (0 - 13.86)
Acute kidney failure	0	0.00%	0 (0 - 0)	0	0.00%	0 (0 - 0)	0	0.00%	0 (0 - 0)	0	0.00%	0 (0 - 0)
Acute liver failure	2	1.57%	0.60 (0 - 1.94)	1	1.56%	0.60 (0 - 2.49)	1	1.92%	0.75 (0 - 3.1)	0	0.00%	0 (0 - 0)
**Event Rates[3]**												
**3 months since index date**												
AMI	9	7.09%	41.06 (32.5 - 49.62)	4	6.25%	41.37 (29.3 - 53.44)	5	9.62%	49.95 (36.36 - 63.54)	0	0.00%	0 (0 - 0)
Ischemic stroke	3	2.36%	10.26 (4.98 - 15.54)	2	3.13%	13.79 (5.34 - 22.24)	1	1.92%	8.33 (0.82 - 15.84)	0	0.00%	0 (0 - 0)
Acute kidney failure	0	0.00%	0 (0 - 0)	0	0.00%	0 (0 - 0)	0	0.00%	0 (0 - 0)	0	0.00%	0 (0 - 0)
Acute liver failure	0	0.00%	0 (0 - 0)	0	0.00%	0 (0 - 0)	0	0.00%	0 (0 - 0)	0	0.00%	0 (0 - 0)
**6 months since index date**												
AMI	10	7.87%	23.92 (16.5 - 31.34)	5	7.81%	27.56 (16.61 - 38.51)	5	9.62%	25.01 (13.24 - 36.78)	0	0.00%	0 (0 - 0)
Ischemic stroke	4	3.15%	6.83 (2.44 - 11.22)	2	3.13%	6.89 (0.68 - 13.1)	2	3.85%	8.34 (0.83 - 15.85)	0	0.00%	0 (0 - 0)
Acute kidney failure	0	0.00%	0 (0 - 0)	0	0.00%	0 (0 - 0)	0	0.00%	0 (0 - 0)	0	0.00%	0 (0 - 0)
Acute liver failure	0	0.00%	0 (0 - 0)	0	0.00%	0 (0 - 0)	0	0.00%	0 (0 - 0)	0	0.00%	0 (0 - 0)
**12 months since index date**												
AMI	12	9.45%	15.63 (9.31 - 21.95)	5	7.81%	13.93 (5.45 - 22.41)	7	13.46%	21.34 (10.2 - 32.48)	0	0.00%	0 (0 - 0)

Ischemic stroke	5	3.94%	4.34 (0.8 - 7.88)	3	4.69%	5.22 (0 - 10.67)	2	3.85%	4.27 (0 - 9.77)	0	0.00%	0 (0 - 0)
Acute kidney failure	0	0.00%	0 (0 - 0)	0	0.00%	0 (0 - 0)	0	0.00%	0 (0 - 0)	0	0.00%	0 (0 - 0)
Acute liver failure	1	0.79%	0.87 (0 - 2.49)	0	0.00%	0 (0 - 0)	1	1.92%	2.13 (0 - 6.05)	0	0.00%	0 (0 - 0)
**2 years since index date**												
AMI	12	9.45%	7.98 (3.27 - 12.69)	5	7.81%	7.05 (0.78 - 13.32)	7	13.46%	10.98 (2.48 - 19.48)	0	0.00%	0 (0 - 0)
Ischemic stroke	7	5.51%	3.55 (0.33 - 6.77)	4	6.25%	4.40 (0 - 9.42)	3	5.77%	3.29 (0 - 8.14)	0	0.00%	0 (0 - 0)
Acute kidney failure	0	0.00%	0 (0 - 0)	0	0.00%	0 (0 - 0)	0	0.00%	0 (0 - 0)	0	0.00%	0 (0 - 0)
Acute liver failure	1	0.79%	0.44 (0 - 1.59)	0	0.00%	0 (0 - 0)	1	1.92%	1.10 (0 - 3.93)	0	0.00%	0 (0 - 0)
**3 years since index date**												
AMI	14	11.02%	6.00 (1.87 - 10.13)	6	9.38%	5.34 (0 - 10.85)	8	15.38%	8.20 (0.74 - 15.66)	0	0.00%	0 (0 - 0)
Ischemic stroke	8	6.30%	2.70 (0 - 5.52)	4	6.25%	2.97 (0 - 7.13)	3	5.77%	2.24 (0 - 6.26)	1	9.09%	3.24 (0 - 13.7)
Acute kidney failure	0	0.00%	0 (0 - 0)	0	0.00%	0 (0 - 0)	0	0.00%	0 (0 - 0)	0	0.00%	0 (0 - 0)
Acute liver failure	2	1.57%	0.60 (0 - 1.94)	1	1.56%	0.59 (0 - 2.47)	1	1.92%	0.75 (0 - 3.1)	0	0.00%	0 (0 - 0)

Time window[1]	Trauma bleeding (N=62)			Genitourinary (N=1130)			Respiratory (N=211)			Other MB (N=1704)		
	n	%	Rates (95% CI)	n	%	Rates (95% CI)	n	%	Rates (95% CI)	n	%	Rates (95% CI)
**Incidence Rates[2]**												
**3 months since index date**												
Death from any cause	4	6.45%	26.94 (15.9 - 37.98)	13	1.15%	4.69 (3.46 - 5.92)	6	2.84%	11.78 (7.43 - 16.13)	27	1.58%	6.48 (5.31 - 7.65)
Cardiovascular death	1	1.61%	6.74 (0.5 - 12.98)	4	0.35%	1.44 (0.75 - 2.13)	1	0.47%	1.96 (0.09 - 3.83)	7	0.41%	1.68 (1.07 - 2.29)
AMI	1	1.61%	6.78 (0.52 - 13.04)	9	0.80%	3.26 (2.22 - 4.3)	1	0.47%	1.97 (0.09 - 3.85)	17	1.00%	4.1 (3.16 - 5.04)
Ischemic stroke	1	1.61%	6.81 (0.54 - 13.08)	6	0.53%	2.17 (1.32 - 3.02)	0	0.00%	0 (0 - 0)	16	0.94%	3.86 (2.95 - 4.77)
Acute kidney failure	0	0.00%	0 (0 - 0)	0	0.00%	0 (0 - 0)	0	0.00%	0 (0 - 0)	1	0.06%	0.24 (0.01 - 0.47)
Acute liver failure	2	3.23%	13.64 (5.1 - 22.18)	2	0.18%	0.72 (0.23 - 1.21)	0	0.00%	0 (0 - 0)	3	0.18%	0.72 (0.32 - 1.12)
**6 months since index date**												
Death from any cause	5	8.06%	16.97 (7.63 - 26.31)	18	1.59%	3.21 (2.18 - 4.24)	7	3.32%	6.79 (3.4 - 10.18)	61	3.58%	7.26 (6.03 - 8.49)
Cardiovascular death	1	1.61%	3.39 (0 - 7.89)	5	0.44%	0.89 (0.34 - 1.44)	1	0.47%	0.97 (0 - 2.29)	14	0.82%	1.67 (1.06 - 2.28)
AMI	1	1.61%	3.41 (0 - 7.93)	14	1.24%	2.51 (1.6 - 3.42)	3	1.42%	2.93 (0.65 - 5.21)	29	1.70%	3.48 (2.61 - 4.35)
Ischemic stroke	1	1.61%	3.41 (0 - 7.93)	10	0.88%	1.79 (1.02 - 2.56)	3	1.42%	2.92 (0.65 - 5.19)	29	1.70%	3.48 (2.61 - 4.35)
Acute kidney failure	0	0.00%	0 (0 - 0)	0	0.00%	0 (0 - 0)	0	0.00%	0 (0 - 0)	2	0.12%	0.24 (0.01 - 0.47)
Acute liver failure	2	3.23%	6.89 (0.59 - 13.19)	3	0.27%	0.54 (0.11 - 0.97)	0	0.00%	0 (0 - 0)	5	0.29%	0.6 (0.23 - 0.97)
**12 months since index date**												
Death from any cause	6	9.68%	10.45 (2.84 - 18.06)	23	2.04%	2.07 (1.24 - 2.9)	11	5.21%	5.38 (2.34 - 8.42)	81	4.75%	4.9 (3.88 - 5.92)
Cardiovascular death	1	1.61%	1.74 (0 - 4.99)	8	0.71%	0.72 (0.23 - 1.21)	2	0.95%	0.98 (0 - 2.31)	22	1.29%	1.33 (0.79 - 1.87)
AMI	2	3.23%	3.50 (0 - 8.07)	19	1.68%	1.72 (0.96 - 2.48)	3	1.42%	1.48 (0 - 3.11)	47	2.76%	2.87 (2.08 - 3.66)
Ischemic stroke	2	3.23%	3.51 (0 - 8.09)	19	1.68%	1.72 (0.96 - 2.48)	6	2.84%	2.97 (0.68 - 5.26)	43	2.52%	2.63 (1.87 - 3.39)
Acute kidney failure	0	0.00%	0 (0 - 0)	0	0.00%	0 (0 - 0)	0	0.00%	0 (0 - 0)	2	0.12%	0.12 (0 - 0.28)
Acute liver failure	3	4.84%	5.30 (0 - 10.88)	6	0.53%	0.54 (0.11 - 0.97)	0	0.00%	0 (0 - 0)	6	0.35%	0.36 (0.08 - 0.64)
**2 years since index date**												
Death from any cause	7	11.29%	6.23 (0.21 - 12.25)	31	2.74%	1.4 (0.71 - 2.09)	14	6.64%	3.48 (1.01 - 5.95)	118	6.92%	3.62 (2.73 - 4.51)
Cardiovascular death	1	1.61%	0.89 (0 - 3.23)	11	0.97%	0.5 (0.09 - 0.91)	4	1.90%	0.99 (0 - 2.33)	36	2.11%	1.1 (0.6 - 1.6)
AMI	3	4.84%	2.70 (0 - 6.73)	39	3.45%	1.79 (1.02 - 2.56)	6	2.84%	1.51 (0 - 3.16)	83	4.87%	2.6 (1.84 - 3.36)
Ischemic stroke	4	6.45%	3.62 (0 - 8.27)	40	3.54%	1.83 (1.05 - 2.61)	13	6.16%	3.32 (0.9 - 5.74)	71	4.17%	2.22 (1.52 - 2.92)
Acute kidney failure	0	0.00%	0 (0 - 0)	0	0.00%	0 (0 - 0)	0	0.00%	0 (0 - 0)	2	0.12%	0.06 (0 - 0.18)
Acute liver failure	3	4.84%	2.69 (0 - 6.72)	7	0.62%	0.32 (0 - 0.65)	0	0.00%	0 (0 - 0)	8	0.47%	0.25 (0.01 - 0.49)
**3 years since index date**												
Death from any cause	7	11.29%	4.21 (0 - 9.21)	42	3.72%	1.27 (0.62 - 1.92)	16	7.58%	2.67 (0.49 - 4.85)	142	8.33%	2.94 (2.14 - 3.74)
Cardiovascular death	1	1.61%	0.60 (0 - 2.52)	12	1.06%	0.36 (0.01 - 0.71)	4	1.90%	0.67 (0 - 1.77)	44	2.58%	0.91 (0.46 - 1.36)
AMI	5	8.06%	3.06 (0 - 7.35)	59	5.22%	1.83 (1.05 - 2.61)	11	5.21%	1.87 (0.04 - 3.7)	105	6.16%	2.23 (1.53 - 2.93)
Ischemic stroke	5	8.06%	3.12 (0 - 7.45)	47	4.16%	1.45 (0.75 - 2.15)	15	7.11%	2.6 (0.45 - 4.75)	92	5.40%	1.95 (1.29 - 2.61)
Acute kidney failure	0	0.00%	0 (0 - 0)	0	0.00%	0 (0 - 0)	0	0.00%	0 (0 - 0)	3	0.18%	0.06 (0 - 0.18)
Acute liver failure	3	4.84%	1.81 (0 - 5.13)	11	0.97%	0.33 (0 - 0.66)	0	0.00%	0 (0 - 0)	14	0.82%	0.29 (0.03 - 0.55)
**Event Rates[3]**												
**3 months since index date**												
AMI	1	1.61%	6.74 (0.5 - 12.98)	9	0.80%	3.25 (2.22 - 4.28)	1	0.47%	1.96 (0.09 - 3.83)	17	1.00%	4.32 (3.35 - 5.29)
Ischemic stroke	1	1.61%	6.74 (0.5 - 12.98)	6	0.53%	2.53 (1.61 - 3.45)	0	0.00%	0 (0 - 0)	16	0.94%	4.08 (3.14 - 5.02)
Acute kidney failure	0	0.00%	0 (0 - 0)	0	0.00%	0 (0 - 0)	0	0.00%	0 (0 - 0)	1	0.06%	0.48 (0.15 - 0.81)
Acute liver failure	2	3.23%	13.47 (4.97 - 21.97)	2	0.18%	0.72 (0.23 - 1.21)	0	0.00%	0 (0 - 0)	3	0.18%	0.72 (0.32 - 1.12)
**6 months since index date**												
AMI	1	1.61%	6.79 (0.53 - 13.05)	14	1.24%	3.03 (2.03 - 4.03)	3	1.42%	2.91 (0.64 - 5.18)	29	1.70%	4.53 (3.54 - 5.52)
Ischemic stroke	1	1.61%	6.79 (0.53 - 13.05)	10	0.88%	2.14 (1.3 - 2.98)	3	1.42%	2.91 (0.64 - 5.18)	29	1.70%	4.88 (3.86 - 5.9)
Acute kidney failure	0	0.00%	0 (0 - 0)	0	0.00%	0 (0 - 0)	0	0.00%	0 (0 - 0)	2	0.12%	0.36 (0.08 - 0.64)
Acute liver failure	2	3.23%	6.79 (0.53 - 13.05)	3	0.27%	0.54 (0.11 - 0.97)	0	0.00%	0 (0 - 0)	5	0.29%	0.71 (0.31 - 1.11)
**12 months since index date**												
AMI	2	3.23%	6.97 (0.63 - 13.31)	19	1.68%	1.98 (1.17 - 2.79)	3	1.42%	1.96 (0.09 - 3.83)	47	2.76%	3.81 (2.9 - 4.72)

Ischemic stroke	2	3.23%	5.22 (0 - 10.76)	19	1.68%	2.07 (1.24 - 2.9)	6	2.84%	3.91 (1.29 - 6.53)	43	2.52%	3.81 (2.9 - 4.72)
Acute kidney failure	0	0.00%	0 (0 - 0)	0	0.00%	0 (0 - 0)	0	0.00%	0 (0 - 0)	2	0.12%	0.18 (0 - 0.38)
Acute liver failure	3	4.84%	5.22 (0 - 10.76)	6	0.53%	0.54 (0.11 - 0.97)	0	0.00%	0 (0 - 0)	6	0.35%	0.48 (0.15 - 0.81)
**2 years since index date**												
AMI	3	4.84%	4.45 (0 - 9.58)	39	3.45%	2.17 (1.32 - 3.02)	6	2.84%	1.99 (0.11 - 3.87)	83	4.87%	3.41 (2.55 - 4.27)
Ischemic stroke	4	6.45%	5.34 (0 - 10.94)	40	3.54%	2.22 (1.36 - 3.08)	13	6.16%	3.97 (1.34 - 6.6)	71	4.17%	3.16 (2.33 - 3.99)
Acute kidney failure	0	0.00%	0 (0 - 0)	0	0.00%	0 (0 - 0)	0	0.00%	0 (0 - 0)	2	0.12%	0.09 (0 - 0.23)
Acute liver failure	3	4.84%	2.67 (0 - 6.68)	7	0.62%	0.41 (0.04 - 0.78)	0	0.00%	0 (0 - 0)	8	0.47%	0.31 (0.05 - 0.57)
**3 years since index date**												
AMI	5	8.06%	4.21 (0 - 9.21)	59	5.22%	2.21 (1.35 - 3.07)	11	5.21%	2.67 (0.49 - 4.85)	105	6.16%	2.9 (2.1 - 3.7)
Ischemic stroke	5	8.06%	4.21 (0 - 9.21)	47	4.16%	1.73 (0.97 - 2.49)	15	7.11%	3.00 (0.7 - 5.3)	92	5.40%	2.75 (1.97 - 3.53)
Acute kidney failure	0	0.00%	0 (0 - 0)	0	0.00%	0 (0 - 0)	0	0.00%	0 (0 - 0)	3	0.18%	0.08 (0 - 0.21)
Acute liver failure	3	4.84%	1.80 (0 - 5.11)	11	0.97%	0.42 (0.04 - 0.8)	0	0.00%	0 (0 - 0)	14	0.82%	0.33 (0.06 - 0.6)

N: number of patients in the total group or subgroup or with the event; Incidence rates (95 confidence interval): per 100 person-years; qualitative variables are presented as absolute and relative (%) frequencies.

1. Time window: cumulative events from index date (day of the first major bleeding); 2. Incidence rates defined as the total number of incident events of interest divided by the total person time at risk; 3. Event rates defined as the total number of events, including recurrent events divided by the total person time of follow-up. AMI: acute myocardial infarction; GIB: gastrointestinal bleeding; ICH: intracranial hemorrhage; ICeH: Intracerebral hemorrhage; MB: major bleeding; SAH: subarachnoid hemorrhage.

Supplementary Table S5. Cumulative outpatient visits and hospitalization for 3 years from index date in the overall study population and according to the first major bleeding type.

Time window[1]	OVERALL GROUP (N=4089)				GIB (N=917)				ICH (N=127)				Trauma bleeding (N=62)			
	Patients with visits (n)	Visits (n)	% of patients	Rate, Number of Visits per 100 Patient-Years (95% CI)	Patients with visits (n)	Visits (n)	% of patients	Rate, Number of Visits per 100 Patient-Years (95% CI)	Patients with visits (n)	Visits (n)	% of patients	Rate, Number of Visits per 100 Patient-Years (95% CI)	Patients with visits (n)	Visits (n)	% of patients	Rate, Number of Visits per 100 Patient-Years (95% CI)
**Cumulative HCRU**																
**6 months since index date**																
**All-cause HCRU**																
Outpatient Visits[2]	4000	15775	97.82%	783.60 (770.98 - 796.22)	900	3524	98.15%	781.05 (754.28 - 807.82)	122	470	96.06%	803.02 (733.85 - 872.19)	60	235	96.77%	797.54 (697.52 - 897.56)
GPs visits	3857	11987	94.33%	595.44 (580.4 - 610.48)	873	2670	95.20%	591.77 (559.96 - 623.58)	116	361	91.34%	616.79 (532.23 - 701.35)	58	172	93.55%	583.73 (461.03 - 706.43)
Specialist visits	3190	3788	78.01%	188.16 (176.18 - 200.14)	724	854	78.95%	189.28 (163.93 - 214.63)	95	109	74.80%	186.23 (118.52 - 253.94)	52	63	83.87%	213.81 (111.75 - 315.87)
Investigations[3]	4089	5152	100.00%	255.92 (242.54 - 269.3)	917	1152	100.00%	255.33 (227.11 - 283.55)	127	166	100.00%	283.62 (205.22 - 362.02)	62	80	100.00%	271.50 (160.8 - 382.2)
Hospitalization	4089	4920	100.00%	244.40 (231.23 - 257.57)	917	1097	100.00%	243.14 (215.37 - 270.91)	127	171	100.00%	292.16 (213.07 - 371.25)	62	77	100.00%	261.32 (151.96 - 370.68)
**Bleeding-related HCRU**																
Outpatient Visits[2]	4000	14985	97.82%	744.36 (730.99 - 757.73)	900	3345	98.15%	741.38 (713.04 - 769.72)	122	450	96.06%	768.85 (695.53 - 842.17)	60	223	96.77%	756.81 (650.02 - 863.6)
GPs visits	3857	11197	94.33%	556.2 (540.97 - 571.43)	873	2491	95.20%	552.1 (519.91 - 584.29)	116	341	91.34%	582.62 (496.85 - 668.39)	58	160	93.55%	543 (419 - 667)
Specialist visits	3190	3788	78.01%	188.16 (176.18 - 200.14)	724	854	78.95%	189.28 (163.93 - 214.63)	95	109	74.80%	186.23 (118.52 - 253.94)	52	63	83.87%	213.81 (111.75 - 315.87)

Investigations[3]	4089	4393	100.00%	218.22 (205.56 - 230.88)	917	995	100.00%	220.53 (193.69 - 247.37)	127	134	100.00%	228.95 (155.88 - 302.02)	62	67	100.00%	227.38 (123.05 - 331.71)
Hospitalization	4089	4360	100.00%	216.58 (203.95 - 229.21)	917	980	100.00%	217.21 (190.52 - 243.9)	127	151	100.00%	257.99 (181.89 - 334.09)	62	71	100.00%	240.96 (134.51 - 347.41)
**1 year since index date**																
**All-cause HCRU**																
Outpatient Visits[2]	4080	26607	99.78%	669.23 (654.81 - 683.65)	914	5960	99.67%	668.96 (638.5 - 699.42)	126	793	99.21%	688.55 (608.01 - 769.09)	62	397	100.00%	691.31 (576.32 - 806.3)
GPs visits	4043	19917	98.88%	500.96 (485.63 - 516.29)	910	4441	99.24%	498.46 (466.1 - 530.82)	126	596	99.21%	517.5 (430.59 - 604.41)	62	291	100.00%	506.73 (382.28 - 631.18)
Specialist visits	3658	6690	89.46%	168.27 (156.8 - 179.74)	830	1519	90.51%	170.49 (146.15 - 194.83)	111	197	87.40%	171.05 (105.56 - 236.54)	56	106	90.32%	184.58 (88.01 - 281.15)
Investigations[3]	4089	6536	100.00%	164.4 (153.04 - 175.76)	917	1473	100.00%	165.33 (141.29 - 189.37)	127	210	100.00%	182.34 (115.18 - 249.5)	62	106	100.00%	184.58 (88.01 - 281.15)
Hospitalization	4089	5187	100.00%	130.47 (120.15 - 140.79)	917	1162	100.00%	130.42 (108.62 - 152.22)	127	182	100.00%	158.03 (94.59 - 221.47)	62	85	100.00%	148.01 (59.62 - 236.4)
**Bleeding-related HCRU**																
Outpatient Visits[2]	4080	18368	99.78%	462 (446.72 - 477.28)	914	4107	99.67%	460.97 (428.71 - 493.23)	126	549	99.21%	476.69 (389.82 - 563.56)	62	276	100.00%	480.61 (356.24 - 604.98)
GPs visits	4043	13974	98.88%	351.48 (336.85 - 366.11)	910	3112	99.24%	349.29 (318.43 - 380.15)	126	416	99.21%	361.21 (277.67 - 444.75)	62	207	100.00%	360.46 (240.94 - 479.98)
Specialist visits	3658	4394	89.46%	110.52 (100.91 - 120.13)	830	995	90.51%	111.68 (91.29 - 132.07)	111	133	87.40%	115.48 (59.89 - 171.07)	56	69	90.32%	120.15 (39.22 - 201.08)
Investigations[3]	4089	5428	100.00%	136.53 (126.01 - 147.05)	917	1235	100.00%	138.62 (116.25 - 160.99)	127	177	100.00%	153.69 (90.96 - 216.42)	62	86	100.00%	149.75 (60.93 - 238.57)
Hospitalization	4089	4475	100.00%	112.56 (102.87 - 122.25)	917	1006	100.00%	112.91 (92.43 - 133.39)	127	158	100.00%	137.19 (77.35 - 197.03)	62	75	100.00%	130.6 (46.72 - 214.48)
**2 years since index date**																
**All-cause HCRU**																

Outpatient Visits[2]	4089	48421	100.00%	616.47 (601.57 - 631.37)	917	10872	100.00%	618.8 (587.36 - 650.24)	127	1443	100.00%	639.88 (556.39 - 723.37)	62	732	100.00%	651.42 (532.8 - 770.04)
GPs visits	4085	36736	99.90%	467.7 (452.41 - 482.99)	916	8230	99.89%	468.42 (436.12 - 500.72)	126	1108	99.21%	491.33 (404.38 - 578.28)	62	547	100.00%	486.79 (362.37 - 611.21)
Specialist visits	3942	11685	96.40%	148.77 (137.86 - 159.68)	892	2642	97.27%	150.37 (127.24 - 173.5)	118	335	92.91%	148.55 (86.7 - 210.4)	58	185	93.55%	164.63 (72.32 - 256.94)
Investigations[3]	4089	9767	100.00%	124.35 (114.24 - 134.46)	917	2189	100.00%	124.59 (103.21 - 145.97)	127	315	100.00%	139.68 (79.39 - 199.97)	62	159	100.00%	141.5 (54.74 - 228.26)
Hospitalization	4089	5569	100.00%	70.9 (69.51 - 72.29)	917	1245	100.00%	70.86 (67.92 - 73.8)	127	186	100.00%	82.48 (75.87 - 89.09)	62	96	100.00%	85.43 (76.65 - 94.21)
**Bleeding-related HCRU**																
Outpatient Visits[2]	4089	22899	100.00%	291.54 (277.61 - 305.47)	917	5156	100.00%	293.46 (263.99 - 322.93)	127	695	100.00%	308.19 (227.88 - 388.5)	62	345	100.00%	307.02 (192.2 - 421.84)
GPs visits	4079	17460	99.76%	222.29 (209.55 - 235.03)	916	3918	99.89%	223 (196.06 - 249.94)	127	518	100.00%	229.7 (156.54 - 302.86)	62	259	100.00%	230.49 (125.66 - 335.32)
Specialist visits	4089	5439	100.00%	69.25 (67.84 - 70.66)	917	1238	100.00%	70.46 (67.51 - 73.41)	127	177	100.00%	78.49 (71.34 - 85.64)	62	86	100.00%	76.53 (65.98 - 87.08)
Investigations[3]	4089	6391	100.00%	81.37 (80.18 - 82.56)	917	1440	100.00%	81.96 (79.47 - 84.45)	127	202	100.00%	89.57 (84.25 - 94.89)	62	100	100.00%	88.99 (81.2 - 96.78)
Hospitalization	4089	4697	100.00%	59.8 (58.3 - 61.3)	917	1058	100.00%	60.22 (57.05 - 63.39)	127	162	100.00%	71.84 (64.02 - 79.66)	62	79	100.00%	70.3 (58.93 - 81.67)
**3 years since index date**																
**All-cause HCRU**																
Outpatient Visits[2]	4089	66113	100.00%	566.82 (551.63 - 582.01)	917	14856	100.00%	570.09 (538.05 - 602.13)	127	1991	100.00%	596.85 (511.54 - 682.16)	62	985	100.00%	592.05 (469.72 - 714.38)
GPs visits	4088	49704	99.98%	426.14 (410.98 - 441.3)	916	11164	99.89%	428.41 (396.38 - 460.44)	127	1514	100.00%	453.86 (367.27 - 540.45)	62	730	100.00%	438.78 (315.26 - 562.3)
Specialist visits	4044	16409	98.90%	140.68 (130.02 - 151.34)	909	3692	99.13%	141.68 (119.11 - 164.25)	122	477	96.06%	142.99 (82.11 - 203.87)	60	255	96.77%	153.27 (63.6 - 242.94)

Investigations[3]	4089	12646	100.00%	108.42 (98.89 - 117.95)	917	2843	100.00%	109.1 (88.92 - 129.28)	127	407	100.00%	122.01 (65.09 - 178.93)	62	197	100.00%	118.41 (37.99 - 198.83)
Hospitalization	4089	5873	100.00%	50.35 (48.82 - 51.88)	917	1311	100.00%	50.31 (47.07 - 53.55)	127	191	100.00%	57.26 (48.66 - 65.86)	62	103	100.00%	61.91 (49.82 - 74)
**Bleeding-related HCRU**																
Outpatient Visits[2]	4089	26857	100.00%	230.26 (217.36 - 243.16)	917	6070	100.00%	232.93 (205.57 - 260.29)	127	819	100.00%	245.52 (170.66 - 320.38)	62	405	100.00%	243.43 (136.61 - 350.25)
GPs visits	4079	20368	99.76%	174.62 (162.98 - 186.26)	916	4602	99.89%	176.6 (151.92 - 201.28)	127	603	100.00%	180.76 (113.83 - 247.69)	62	301	100.00%	180.92 (85.1 - 276.74)
Specialist visits	4089	6489	100.00%	55.63 (54.11 - 57.15)	917	1468	100.00%	56.33 (53.12 - 59.54)	127	216	100.00%	64.75 (56.44 - 73.06)	62	104	100.00%	62.51 (50.46 - 74.56)
Investigations[3]	4089	7505	100.00%	64.34 (62.87 - 65.81)	917	1672	100.00%	64.16 (61.06 - 67.26)	127	238	100.00%	71.35 (63.49 - 79.21)	62	112	100.00%	67.32 (55.64 - 79)
Hospitalization	4089	4859	100.00%	41.66 (40.15 - 43.17)	917	1088	100.00%	41.75 (38.56 - 44.94)	127	169	100.00%	50.66 (41.96 - 59.36)	62	82	100.00%	49.29 (36.85 - 61.73)

Time window[1]	Genitourinary (N=1130)				Respiratory (N=211)				Other MB (N=1704)			
	Patients with visits (n)	Visits (n)	% of patients	Number of Visits per 100 Patient-Years (95% CI)	Patients with visits (n)	Visits (n)	% of patients	Rate, Number of Visits per 100 Patient-Years (95% CI)	Patients with visits (n)	Visits (n)	% of patients	Rate, Number of Visits per 100 Patient-Years (95% CI)
	**Cumulative HCRU**											
	**6 months since index date**											
	**All-cause HCRU**											
Outpatient Visits[2]	1098	3964	97.17%	707.12 (680.59 - 733.65)	211	953	100.00%	924.55 (888.91 - 960.19)	1669	6864	97.95%	817.38 (799.04 - 835.72)
GPs visits	1047	3011	92.65%	537.12 (508.05 - 566.19)	209	728	99.05%	706.27 (644.81 - 767.73)	1612	5217	94.60%	621.25 (598.22 - 644.28)
Specialist visits	842	953	74.51%	170 (148.1 - 191.9)	174	225	82.46%	218.28 (162.54 - 274.02)	1355	1647	79.52%	196.13 (177.28 - 214.98)
Investigations[3]	1130	1425	100.00%	254.2 (228.81 - 279.59)	211	277	100.00%	268.73 (208.91 - 328.55)	1704	2132	100.00%	253.88 (233.21 - 274.55)
Hospitalization	1130	1335	100.00%	238.14 (213.3 - 262.98)	211	255	100.00%	247.39 (189.17 - 305.61)	1704	2062	100.00%	245.55 (225.11 - 265.99)
	**Bleeding-related HCRU**											
Outpatient Visits[2]	1098	3774	97.17%	673.23 (645.88 - 700.58)	211	896	100.00%	869.26 (823.77 - 914.75)	1669	6520	97.95%	776.41 (756.63 - 796.19)
GPs visits	1047	2821	92.65%	503.23 (474.08 - 532.38)	209	671	99.05%	650.97 (586.65 - 715.29)	1612	4873	94.60%	580.29 (556.86 - 603.72)
Specialist visits	842	953	74.51%	170 (148.1 - 191.9)	174	225	82.46%	218.28 (162.54 - 274.02)	1355	1647	79.52%	196.13 (177.28 - 214.98)

Investigations[3]	1130	1205	100.00%	214.95 (191 - 238.9)	211	231	100.00%	224.1 (167.83 - 280.37)	1704	1828	100.00%	217.68 (198.09 - 237.27)
Hospitalization	1130	1175	100.00%	209.6 (185.87 - 233.33)	211	224	100.00%	217.31 (161.66 - 272.96)	1704	1830	100.00%	217.92 (198.32 - 237.52)
	**1 year since index date**											
	**All-cause HCRU**											
Outpatient Visits[2]	1128	6775	99.82%	609.28 (580.83 - 637.73)	211	1556	100.00%	761.41 (703.9 - 818.92)	1701	11523	99.82%	696.96 (675.14 - 718.78)
GPs visits	1110	5081	98.23%	456.93 (427.89 - 485.97)	211	1157	100.00%	566.16 (499.29 - 633.03)	1686	8642	98.94%	522.7 (498.98 - 546.42)
Specialist visits	991	1694	87.70%	152.34 (131.39 - 173.29)	199	399	94.31%	195.24 (141.75 - 248.73)	1527	2881	89.61%	174.26 (156.25 - 192.27)
Investigations[3]	1130	1786	100.00%	160.62 (139.21 - 182.03)	211	356	100.00%	174.2 (123.02 - 225.38)	1704	2711	100.00%	163.97 (146.39 - 181.55)
Hospitalization	1130	1391	100.00%	125.09 (105.8 - 144.38)	211	272	100.00%	133.1 (87.27 - 178.93)	1704	2180	100.00%	131.86 (115.8 - 147.92)
	**Bleeding-related HCRU**											
Outpatient Visits[2]	1128	4672	99.82%	420.15 (391.37 - 448.93)	211	1074	100.00%	525.55 (458.17 - 592.93)	1701	7966	99.82%	481.82 (458.1 - 505.54)
GPs visits	1110	3559	98.23%	320.06 (292.86 - 347.26)	211	813	100.00%	397.83 (331.79 - 463.87)	1686	6074	98.94%	367.38 (344.49 - 390.27)
Specialist visits	991	1113	87.70%	100.09 (82.59 - 117.59)	199	261	94.31%	127.72 (82.68 - 172.76)	1527	1892	89.61%	114.44 (99.32 - 129.56)
Investigations[3]	1130	1452	100.00%	130.58 (110.93 - 150.23)	211	292	100.00%	142.89 (95.67 - 190.11)	1704	2272	100.00%	137.42 (121.07 - 153.77)
Hospitalization	1130	1197	100.00%	107.65 (89.58 - 125.72)	211	231	100.00%	113.04 (70.31 - 155.77)	1704	1883	100.00%	113.89 (98.81 - 128.97)
	**2 years since index date**											

	All-cause HCRU											
Outpatient Visits[2]	1130	12380	100.00%	560.1 (531.16 - 589.04)	211	2821	100.00%	700.3 (638.48 - 762.12)	1704	20905	100.00%	641.46 (618.69 - 664.23)
GPs visits	1128	9400	99.82%	425.28 (396.45 - 454.11)	211	2126	100.00%	527.77 (460.41 - 595.13)	1704	15872	100.00%	487.03 (463.3 - 510.76)
Specialist visits	1080	2980	95.58%	134.82 (114.91 - 154.73)	207	695	98.10%	172.53 (121.55 - 223.51)	1645	5033	96.54%	154.44 (137.28 - 171.6)
Investigations[3]	1130	2627	100.00%	118.85 (99.98 - 137.72)	211	523	100.00%	129.83 (84.48 - 175.18)	1704	4113	100.00%	126.21 (110.44 - 141.98)
Hospitalization	1130	1479	100.00%	66.91 (64.17 - 69.65)	211	292	100.00%	72.49 (66.46 - 78.52)	1704	2367	100.00%	72.63 (70.51 - 74.75)
	Bleeding-related HCRU											
Outpatient Visits[2]	1130	5925	100.00%	268.06 (242.23 - 293.89)	211	1298	100.00%	322.22 (259.16 - 385.28)	1704	9825	100.00%	301.48 (279.69 - 323.27)
GPs visits	1126	4470	99.65%	202.23 (178.81 - 225.65)	211	1006	100.00%	249.73 (191.32 - 308.14)	1699	7548	99.71%	231.61 (211.58 - 251.64)
Specialist visits	1130	1455	100.00%	65.83 (63.06 - 68.6)	211	292	100.00%	72.49 (66.46 - 78.52)	1704	2277	100.00%	69.87 (67.69 - 72.05)
Investigations[3]	1130	1722	100.00%	77.91 (75.49 - 80.33)	211	348	100.00%	86.39 (81.76 - 91.02)	1704	2679	100.00%	82.2 (80.38 - 84.02)
Hospitalization	1130	1247	100.00%	56.42 (53.53 - 59.31)	211	244	100.00%	60.57 (53.98 - 67.16)	1704	1986	100.00%	60.94 (58.62 - 63.26)
	3 years since index date											
	All-cause HCRU											
Outpatient Visits[2]	1130	17017	100.00%	516.21 (487.07 - 545.35)	211	3815	100.00%	636.33 (571.42 - 701.24)	1704	28434	100.00%	588.89 (565.53 - 612.25)
GPs visits	1130	12813	100.00%	388.68 (360.26 - 417.1)	211	2835	100.00%	472.87 (405.5 - 540.24)	1704	21378	100.00%	442.76 (419.18 - 466.34)

Specialist visits	1114	4204	98.58%	127.53 (108.08 - 146.98)	211	980	100.00%	163.46 (113.56 - 213.36)	1688	7056	99.06%	146.14 (129.37 - 162.91)
Investigations[3]	1130	3374	100.00%	102.35 (84.68 - 120.02)	211	696	100.00%	116.09 (72.87 - 159.31)	1704	5326	100.00%	110.31 (95.44 - 125.18)
Hospitalization	1130	1563	100.00%	47.41 (44.5 - 50.32)	211	303	100.00%	50.54 (43.79 - 57.29)	1704	2505	100.00%	51.88 (49.51 - 54.25)
	**Bleeding-related HCRU**											
Outpatient Visits[2]	1130	6971	100.00%	211.47 (187.66 - 235.28)	211	1526	100.00%	254.53 (195.75 - 313.31)	1704	11471	100.00%	237.57 (217.36 - 257.78)
GPs visits	1126	5237	99.65%	158.87 (137.56 - 180.18)	211	1164	100.00%	194.15 (140.78 - 247.52)	1699	8762	99.71%	181.47 (163.17 - 199.77)
Specialist visits	1130	1734	100.00%	52.6 (49.69 - 55.51)	211	362	100.00%	60.38 (53.78 - 66.98)	1704	2709	100.00%	56.11 (53.75 - 58.47)
Investigations[3]	1130	2036	100.00%	61.76 (58.93 - 64.59)	211	416	100.00%	69.39 (63.17 - 75.61)	1704	3143	100.00%	65.09 (62.83 - 67.35)
Hospitalization	1130	1289	100.00%	39.1 (36.25 - 41.95)	211	253	100.00%	42.2 (35.54 - 48.86)	1704	2060	100.00%	42.66 (40.31 - 45.01)

1. Index date: day of the first major bleed; 2. Outpatient visits: include GP visits and specialized visits; 3. Laboratory/radiology investigations. Qualitative variables are presented as absolute and relative (%) frequencies. Rate (95% CI): per 100 person-years. GIB: gastrointestinal bleeding; GP: general practitioners; HCRU: healthcare resource utilization; ICH: intracranial hemorrhage; MB: major bleeding.

Supplementary Table S6. Cumulative length of hospital stays, number of prescriptions and work absences for 3 years from index date in the overall study population and according to the first major bleeding type.

Time window[1]	Overall group (N= 4089)				GIB (N=917)				ICH (N=127)				Trauma bleeding (N=62)			
Cumulative HCRU per patient	Patients with use of resources (n, %)	Number of units	ALL patients within cohort: Average	ALL patients within cohort: Standard deviation	Patients with use of resources (n, %)	Number of units	ALL patients within cohort: Average	ALL patients within cohort: Standard deviation	Patients with use of resources (n, %)	Number of units	ALL patients within cohort: Average	ALL patients within cohort: Standard deviation	Patients with use of resources (n, %)	Number of units	ALL patients within cohort: Average	ALL patients within cohort: Standard deviation
**6 months since index date**																
**All-cause HCRU**																
Length of hospital stays (days)	4089 (100)	40962	10.02	3.80	917 (100)	9130	9.96	3.74	127 (100)	1474	11.61	6.30	62 (100)	622	10.03	3.86
Number of Prescriptions	4089 (100)	21808	5.33	1.99	917 (100)	4878	5.32	1.98	127 (100)	728	5.73	2.23	62 (100)	319	5.15	2.09
Work absences (days)	3114 (76)	76913	18.81	13.09	722 (79)	17579	19.17	12.70	102 (80)	2999	23.61	18.31	44 (71)	1091	17.60	13.15
**Bleeding-related HCRU**																
Length of hospital stays (days)	4089 (100)	36446	8.91	2.55	917 (100)	8164	8.90	2.49	127 (100)	1302	10.25	5.27	62 (100)	573	9.24	3.00
Number of Prescriptions	3606 (88)	6720	1.64	0.98	808 (88)	1480	1.61	0.96	111 (87)	226	1.78	1.10	53 (85)	95	1.53	0.95
Work absences (days)	3114 (76)	69968	17.11	10.81	722 (79)	15987	17.43	10.33	102 (80)	2707	21.31	16.21	44 (71)	1014	16.35	11.37
**1 year since index date**																
**All-cause HCRU**																
Length of hospital stays (days)	4089 (100)	42432	10.38	4.18	917 (100)	9349	10.20	4.02	127 (100)	1511	11.90	6.44	62 (100)	673	10.85	4.74
Number of Prescriptions	4089 (100)	43948	10.75	3.04	917 (100)	10094	11.01	3.41	127 (100)	1403	11.05	3.96	62 (100)	652	10.52	3.14
Work absences (days)	3114 (76)	79194	19.37	13.74	722 (79)	17846	19.46	13.10	102 (80)	3044	23.97	18.53	44 (71)	1163	18.76	14.57
**Bleeding-related HCRU**																
Length of hospital stays (days)	4089 (100)	37088	9.07	2.87	917 (100)	8282	9.03	2.80	127 (100)	1332	10.49	5.45	62 (100)	593	9.56	3.56
Number of Prescriptions	3666 (90)	6922	1.69	0.96	818 (89)	1524	1.66	0.95	117 (92)	240	1.89	1.03	55 (89)	99	1.60	0.91
Work absences (days)	3114 (76)	70719	17.29	11.10	722 (79)	16078	17.53	10.55	102 (80)	2754	21.69	16.54	44 (71)	1014	16.35	11.37
**2 years since index date**																
**All-cause HCRU**																

Length of hospital stays (days)	4089 (100)	44615	10.91	4.87	917 (100)	9628	10.50	4.53	127 (100)	1530	12.05	6.47	62 (100)	762	12.29	6.79
Number of Prescriptions	4089 (100)	67452	16.50	3.82	917 (100)	15468	16.87	4.49	127 (100)	2141	16.86	4.00	62 (100)	1029	16.60	3.83
Work absences (days)	3114 (76)	83692	20.47	15.61	722 (79)	18253	19.91	13.93	102 (80)	3107	24.46	18.84	44 (71)	1356	21.87	19.98
**Bleeding-related HCRU**																
Length of hospital stays (days)	4089 (100)	38147	9.33	3.30	917 (100)	8397	9.16	2.93	127 (100)	1332	10.49	5.45	62 (100)	624	10.06	3.97
Number of Prescriptions	3720 (91)	7013	1.72	0.94	833 (91)	1551	1.69	0.93	118 (93)	242	1.91	1.02	56 (90)	101	1.63	0.89
Work absences (days)	3114 (76)	72049	17.62	11.61	722 (79)	16174	17.64	10.68	102 (80)	2754	21.69	16.54	44 (71)	1062	17.13	12.50
**3 years since index date**																
**All-cause HCRU**																
Length of hospital stays (days)	4089 (100)	46308	11.33	5.28	917 (100)	9850	10.74	4.75	127 (100)	1541	12.13	6.48	62 (100)	824	13.29	7.71
Number of Prescriptions	4089 (100)	86978	21.27	4.02	917 (100)	18795	20.50	4.42	127 (100)	2773	21.83	3.72	62 (100)	1322	21.32	4.24
Work absences (days)	3114 (76)	87390	21.37	16.92	722 (79)	18665	20.35	14.61	102 (80)	3107	24.46	18.84	44 (71)	1557	25.11	24.39
**Bleeding-related HCRU**																
Length of hospital stays (days)	4089 (100)	39044	9.55	3.64	917 (100)	8508	9.28	3.16	127 (100)	1352	10.65	5.57	62 (100)	643	10.37	4.24
Number of Prescriptions	3768 (92)	7161	1.75	0.92	843 (92)	1584	1.73	0.91	119 (94)	246	1.94	1.00	56 (90)	101	1.63	0.89
Work absences (days)	3114 (76)	73415	17.95	12.10	722 (79)	16314	17.79	10.89	102 (80)	2754	21.69	16.54	44 (71)	1108	17.87	13.51

Time window[1]	Overall group (N= 4089)				Genitourinary (N=1130)				Respiratory (N=211)				Other MB (N=1704)			
	Patients with use of resources (n, %)	Number of units	ALL patients within cohort		Patients with use of resources (n, %)	Number of units	ALL patients within cohort		Patients with use of resources (n, %)	Number of units	ALL patients within cohort		Patients with use of resources (n, %)	Number of units	ALL patients within cohort	
Cumulative HCRU per patient			Average	Standard deviation			Average	Standard deviation			Average	Standard deviation			Average	Standard deviation
**6 months since index date**																
**All-cause HCRU**																
Length of hospital stays (days)	4089 (100)	40962	10.02	3.80	1130 (100)	11525	10.20	3.69	211 (100)	2007	9.51	3.44	1704 (100)	16826	9.87	3.67
Number of Prescriptions	4089 (100)	21808	5.33	1.99	1130 (100)	6060	5.36	1.97	211 (100)	1147	5.44	1.98	1704 (100)	8995	5.28	2.00
Work absences (days)	3114 (76)	76913	18.81	13.09	948 (84)	23588	20.87	12.17	137 (65)	3246	15.38	13.16	1205 (71)	29501	17.31	13.11
**Bleeding-related HCRU**																
Length of hospital stays (days)	4089 (100)	36446	8.91	2.55	1130 (100)	10173	9.00	2.23	211 (100)	1782	8.45	2.23	1704 (100)	15025	8.82	2.48
Number of Prescriptions	3606 (88)	6720	1.64	0.98	1003 (89)	1886	1.67	0.97	192 (91)	367	1.74	0.94	1492 (88)	2761	1.62	0.98
Work absences (days)	3114 (76)	69968	17.11	10.81	948 (84)	21185	18.75	9.49	137 (65)	2995	14.19	11.39	1205 (71)	27094	15.90	11.05
**1 year since index date**																
**All-cause HCRU**																
Length of hospital stays (days)	4089 (100)	42432	10.38	4.18	1130 (100)	11924	10.55	4.08	211 (100)	2089	9.90	3.73	1704 (100)	17559	10.30	4.14
Number of Prescriptions	4089 (100)	43948	10.75	3.04	1130 (100)	12106	10.71	2.93	211 (100)	2201	10.43	2.68	1704 (100)	18144	10.65	2.86
Work absences (days)	3114 (76)	79194	19.37	13.74	948 (84)	24339	21.54	12.84	137 (65)	3314	15.71	13.55	1205 (71)	30651	17.99	13.96
**Bleeding-related HCRU**																
Length of hospital stays (days)	4089 (100)	37088	9.07	2.87	1130 (100)	10344	9.15	2.56	211 (100)	1823	8.64	2.56	1704 (100)	15307	8.98	2.82
Number of Prescriptions	3666 (90)	6922	1.69	0.96	1014 (90)	1925	1.70	0.95	195 (92)	373	1.77	0.91	1522 (89)	2860	1.68	0.96
Work absences (days)	3114 (76)	70719	17.29	11.10	948 (84)	21487	19.02	9.90	137 (65)	3022	14.32	11.62	1205 (71)	27378	16.07	11.32
**2 years since index date**																
**All-cause HCRU**																
Length of hospital stays (days)	4089 (100)	44615	10.91	4.87	1130 (100)	12499	11.06	4.72	211 (100)	2165	10.26	4.44	1704 (100)	18793	11.03	5.03
Number of Prescriptions	4089 (100)	67452	16.50	3.82	1130 (100)	18567	16.43	3.63	211 (100)	3374	15.99	3.54	1704 (100)	27902	16.37	3.54
Work absences (days)	3114 (76)	83692	20.47	15.61	948 (84)	25843	22.87	15.00	137 (65)	3430	16.26	14.39	1205 (71)	33059	19.40	16.48
**Bleeding-related HCRU**																
Length of hospital stays (days)	4089 (100)	38147	9.33	3.30	1130 (100)	10649	9.42	3.07	211 (100)	1884	8.93	3.39	1704 (100)	15885	9.32	3.39

Number of Prescriptions	3720 (91)	7013	1.72	0.94	1021 (90)	1941	1.72	0.94	197 (93)	376	1.78	0.90	1551 (91)	2903	1.70	0.94
Work absences (days)	3114 (76)	72049	17.62	11.61	948 (84)	22074	19.53	10.74	137 (65)	3068	14.54	11.94	1205 (71)	27979	16.42	11.90
**3 years since index date**																
**All-cause HCRU**																
Length of hospital stays (days)	4089 (100)	46308	11.33	5.28	1130 (100)	13033	11.53	5.11	211 (100)	2224	10.54	4.70	1704 (100)	19660	11.54	5.59
Number of Prescriptions	4089 (100)	86978	21.27	4.02	1130 (100)	24165	21.38	3.80	211 (100)	4585	21.73	3.75	1704 (100)	36660	21.51	3.95
Work absences (days)	3114 (76)	87390	21.37	16.92	948 (84)	27091	23.97	16.29	137 (65)	3560	16.87	15.19	1205 (71)	34967	20.52	18.25
**Bleeding-related HCRU**																
Length of hospital stays (days)	4089 (100)	39044	9.55	3.64	1130 (100)	10917	9.66	3.47	211 (100)	1946	9.22	3.67	1704 (100)	16321	9.58	3.80
Number of Prescriptions	3768 (92)	7161	1.75	0.92	1036 (92)	1981	1.75	0.92	201 (95)	387	1.83	0.86	1569 (92)	2963	1.74	0.92
Work absences (days)	3114 (76)	73415	17.95	12.10	948 (84)	22591	19.99	11.44	137 (65)	3171	15.03	12.64	1205 (71)	28585	16.78	12.43

1. Index date: day of the first major bleed. Quantitative variables are presented as mean and standard deviation; qualitative variables are presented as absolute and relative (%) frequencies.

GIB: gastrointestinal bleeding; HCRU: healthcare resource utilization; ICH: intracranial hemorrhage; MB: major bleeding.

Supplementary Table S7. Cumulative costs for 3 years from index date in the overall study population and according to the first major bleeding type.

	Overall group				GIB				ICH				Trauma bleeding			
**Cumulative costs**	**No. Patients**	**Total cost (Eur)**	**Mean costs per patient (Eur)**	**SD costs per patient (Eur)**	**No. Patients**	**Total cost (Eur)**	**Mean costs per patient (Eur)**	**SD costs per patient (Eur)**	**No. Patients**	**Total cost (Eur)**	**Mean costs per patient (Eur)**	**SD costs per patient (Eur)**	**No. Patients**	**Total cost (Eur)**	**Mean costs per patient (Eur)**	**SD costs per patient (Eur)**
**6 months since index date**																
**All-cause costs**																
**Outpatient**	4089	1.883.101.2	460.5	204.09	917	422.448.2	460.69	194.40	127	57.027.3	449.03	191.83	62	29.184.8	470.72	210.55
GPs visit	4089	768.606.4	188.0	102.61	917	171.200.4	186.70	96.57	127	23.147.3	182.26	99.08	62	11.028.6	177.88	106.04
Specialist visit	4089	896.204.2	219.2	142.49	917	202.048.1	220.34	139.98	127	25.788.3	203.06	138.93	62	14.905.2	240.41	138.76
Investigations[2] (outpatient)	4089	45.406.5	11.1	23.78	917	9.592.1	10.46	23.11	127	1.702.7	13.41	26.23	62	851.4	13.73	26.57
Prescriptions (outpatient)	4089	172.884.1	42.3	25.93	917	39.607.6	43.19	26.70	127	6.388.9	50.31	33.45	62	2.399.6	38.70	27.63
**Inpatient (hospital+all other costs in hospitalization)**	4089	32.839.020.2	8.031.1	2772.13	917	7.426.428.0	8098.61	2723.41	127	1.179.959.6	9291.02	4551.83	62	498.130.5	8034.36	2707.16
Hospitalsations (>24 hours)	4089	29.397.590.7	7.189.4	2726.77	917	6.552.414.5	7145.49	2681.57	127	1.057.859.7	8329.60	4519.29	62	446.396.7	7199.95	2768.81
Investigations[2] (within hospital)	4089	360.746.9	88.2	67.86	917	93.979.0	102.49	79.53	127	12.861.7	101.27	80.60	62	6.120.5	98.72	62.58
Prescriptions (within hospital)	4089	3.080.682.6	753.4	508.31	917	780.034.4	850.64	536.80	127	109.238.3	860.14	576.54	62	45.613.4	735.70	440.17
**Pharmacy & Investigation (global)**	4089	3.659.720.0	895.0	518.92	917	923.213.2	1006.78	545.83	127	130.191.6	1025.13	579.77	62	54.984.8	886.85	466.51
Prescriptions (global)	4089	3.253.566.7	795.7	512.51	917	819.642.0	893.83	540.44	127	115.627.2	910.45	577.01	62	48.012.9	774.40	445.18
Investigations[2] (global)	4089	406.153.3	99.3	71.70	917	103.571.2	112.95	82.17	127	14.564.4	114.68	84.74	62	6.971.8	112.45	66.74
**Indirect cost**																
Cost of absence from work	4089	21.077.922.2	5.154.8	3587.49	917	4.817.505.4	5253.55	3480.61	127	821.872.6	6471.44	5018.06	62	298.987.3	4822.38	3603.51
**Total Overall cost**	4089	55.800.043.6	13.646.4	5699.97	917	12.666.381.6	13812.85	5562.20	127	2.058.859.6	16211.49	9159.48	62	826.302.6	13327.46	5495.54
**Bleeding-related costs**																
**Outpatient**	4089	1.655.696.6	404.9	194.35	917	371.813.3	405.47	185.15	127	49.070.8	386.38	185.26	62	25.752.0	415.35	199.91
GPs visits	4089	717.951.6	175.6	92.75	917	159.722.9	174.18	87.46	127	21.864.9	172.16	91.29	62	10.259.2	165.47	95.42
Specialist visits	4089	896.204.2	219.2	142.49	917	202.048.1	220.34	139.98	127	25.788.3	203.06	138.93	62	14.905.2	240.41	138.76
Investigations[2] (outpatient)	4089	16.119.3	3.9	14.43	917	3.689.3	4.02	14.57	127	397.3	3.13	13.00	62	283.8	4.58	15.58
Prescriptions (outpatient)	4089	25.421.5	6.2	9.82	917	6.353.0	6.93	10.57	127	1.020.2	8.03	12.00	62	303.8	4.90	9.06
**Inpatient - Bleeding episode (hospital+all other costs in bleeding event hospitalization)**	4089	29.182.574.8	7.136.8	1908.12	917	6.630.774.7	7230.94	1851.53	127	1.037.872.6	8172.23	3813.24	62	456.231.0	7358.56	2148.87
Hospitalizations (>24 hours)	4089	26.156.549.8	6.396.8	1832.35	917	5.859.136.0	6389.46	1784.99	127	934.418.8	7357.63	3785.71	62	411.230.4	6632.75	2153.77
Investigations[2] (within hospital)	4089	341.484.8	83.5	60.73	917	89.043.3	97.10	71.09	127	11.891.4	93.63	69.66	62	5.897.7	95.12	60.05
Prescriptions (within hospital)	4089	2.684.540.2	656.5	529.04	917	682.595.3	744.38	569.87	127	91.562.4	720.96	585.43	62	39.102.9	630.69	484.87
**Pharmacy & Investigation (global)**	4089	3.067.565.8	750.2	536.27	917	781.680.9	852.43	576.19	127	104.871.3	825.76	589.72	62	45.588.1	735.29	501.63
Prescriptions	4089	2.709.961.7	662.7	531.13	917	688.948.3	751.31	572.22	127	92.582.6	729.00	585.88	62	39.406.7	635.59	487.79
Investigations[2] (global)	4089	357.604.1	87.5	62.42	917	92.732.6	101.13	72.81	127	12.288.7	96.76	72.00	62	6.181.4	99.70	62.51
**Indirect cost**																
Cost of absence from work	4089	19.174.652.7	4.689.3	2961.47	917	4.381.219.6	4777.77	2830.49	127	741.850.3	5841.34	4442.81	62	277.885.6	4482.03	3115.60

**Total Overall bleeding cost**	4089	50.012.924.1	12.231.1	4304.38	917	11.383.807.6	12414.18	4064.52	127	1.828.793.7	14399.95	7943.04	62	759.868.5	12255.94	4440.38
**1 year since index date**																
**All-cause costs**																
**Outpatient**	4089	3.303.129.4	807.8	319.53	917	745.472.5	812.95	299.66	127	100.517.5	791.48	307.82	62	50.908.7	821.11	360.73
GPs visit	4089	1.277.078.0	312.3	148.44	917	284.756.9	310.53	137.68	127	38.215.5	300.91	136.50	62	18.658.9	300.95	150.75
Specialist visit	4089	1.582.789.3	387.1	221.73	917	359.380.7	391.91	215.90	127	46.608.3	366.99	224.47	62	25.078.6	404.49	232.23
Investigations[2] (outpatient)	4089	105.910.6	25.9	38.49	917	23.668.1	25.81	38.40	127	3.462.2	27.26	40.36	62	1.873.0	30.21	42.02
Prescriptions (outpatient)	4089	337.351.5	82.5	37.70	917	77.666.7	84.70	40.38	127	12.231.4	96.31	45.86	62	5.298.2	85.45	43.17
**Inpatient**	4089	34.039.190.2	8.324.6	3106.92	917	7.608.152.4	8296.79	2967.19	127	1.210.882.1	9534.51	4694.57	62	539.145.2	8695.89	3452.89
Hospitalizations (>24 hours)	4089	30.452.579.7	7.447.4	2998.00	917	6.709.586.3	7316.89	2887.13	127	1.084.413.8	8538.69	4622.74	62	482.998.4	7790.30	3400.77
Investigations[2] (within hospital)	4089	381.223.6	93.2	74.45	917	99.323.3	108.31	86.83	127	13.895.0	109.41	90.75	62	6.779.0	109.34	80.82
Prescriptions (within hospital)	4089	3.205.386.9	783.9	569.73	917	799.242.7	871.58	584.81	127	112.573.3	886.40	623.40	62	49.367.9	796.26	499.13
**Pharmacy & Investigation (global)**	4089	4.029.872.6	985.5	585.87	917	999.900.9	1090.40	602.57	127	142.162.0	1119.39	642.99	62	63.318.1	1021.26	538.98
Prescriptions (global)	4089	3.542.738.4	866.4	578.39	917	876.909.4	956.28	595.65	127	124.804.7	982.71	636.77	62	54.666.1	881.71	511.96
Investigations[2] (global)	4089	487.134.2	119.1	83.04	917	122.991.4	134.12	93.85	127	17.357.2	136.67	98.53	62	8.652.0	139.55	87.08
**Indirect cost**																
Cost of absence from work	4089	21.703.027.7	5.307.7	3764.51	917	4.890.676.5	5333.34	3590.88	127	834.204.8	6568.54	5078.85	62	318.718.9	5140.63	3992.58
**Total Overall cost**	4089	59.045.347.4	14.440.0	6063.03	917	13.244.301.3	14443.08	5760.71	127	2.145.604.4	16894.52	9243.25	62	908.772.8	14657.63	6314.71
**Bleeding-related costs**																
**Outpatient**	4089	2.028.556.7	496.1	205.05	917	455.957.0	497.23	189.21	127	61.942.2	487.73	200.52	62	31.075.9	501.22	232.10
GPs visits	4089	896.012.9	219.1	106.74	917	199.541.4	217.60	99.66	127	26.673.9	210.03	96.16	62	13.272.8	214.08	105.10
Specialist visits	4089	1.039.577.9	254.2	134.15	917	235.407.4	256.71	128.30	127	31.466.5	247.77	139.36	62	16.324.7	263.30	142.76
Investigations[2] (outpatient)	4088	61.866.3	15.1	28.73	916	13.621.9	14.85	28.76	127	2.213.6	17.43	29.92	62	1.078.4	17.39	33.44
Prescriptions (outpatient)	4089	31.099.6	7.6	14.59	917	7.386.3	8.05	13.91	127	1.588.2	12.51	23.21	62	399.9	6.45	11.84
**Inpatient - Bleeding episode (hospital+all other costs in bleeding event hospitalization)**	4089	29.738.351.2	7.272.8	2126.76	917	6.743.239.2	7353.59	2068.12	127	1.063.823.7	8376.56	3951.19	62	471.305.0	7601.69	2514.56
Hospitalizations (>24 hours)	4089	26.617.300.1	6.509.5	2056.88	917	5.943.822.2	6481.81	2009.31	127	955.949.2	7527.16	3914.08	62	425.584.0	6864.26	2555.19
Investigations[2] (within hospital)	4089	361.007.9	88.3	68.47	917	94.975.6	103.57	79.73	127	13.221.0	104.10	88.21	62	6.148.7	99.17	60.40
Prescriptions (within hospital)	4089	2.760.043.2	675.0	551.81	917	704.441.3	768.20	603.91	127	94.653.6	745.30	588.04	62	39.572.3	638.26	482.40
**Pharmacy & Investigation (global)**	4089	3.214.017.1	786.0	559.78	917	820.425.1	894.68	613.83	127	111.676.3	879.34	585.47	62	47.199.4	761.28	499.47
Prescriptions	4089	2.791.142.8	682.6	552.96	917	711.827.6	776.26	606.18	127	96.241.8	757.81	585.02	62	39.972.2	644.71	483.39
Investigations[2] (global)	4089	422.874.3	103.4	73.72	917	108.597.5	118.43	83.34	127	15.434.5	121.53	96.48	62	7.227.1	116.57	69.02
**Indirect cost**																
Cost of absence from work	4089	19.380.463.4	4.739.7	3042.33	917	4.406.158.0	4804.97	2891.57	127	754.730.6	5942.76	4533.25	62	277.885.6	4482.03	3115.60
**Total Overall bleeding cost**	4089	51.147.371.3	12.508.5	4499.23	917	11.605.354.2	12655.78	4210.93	127	1.880.496.6	14807.06	8127.98	62	780.266.5	12584.94	4366.06
**2 years since index date**																
**All-cause costs**																
**Outpatient**	4089	5.946.296.9	1.454.2	530.12	917	1.344.130.7	1465.79	492.27	127	180.418.0	1420.61	514.17	62	92.372.2	1489.87	650.85
GPs visit	4089	2.355.512.3	576.1	253.26	917	527.707.6	575.47	234.05	127	71.045.0	559.41	256.06	62	35.073.6	565.70	278.77
Specialist visit	4089	2.764.558.0	676.1	334.57	917	625.071.6	681.65	316.77	127	79.257.8	624.08	342.03	62	43.769.2	705.96	393.78
Investigations[2] (outpatient)	4089	255.241.2	62.4	59.95	917	55.736.4	60.78	58.12	127	8.400.2	66.14	59.73	62	4.540.6	73.24	66.91
Prescriptions (outpatient)	4089	570.985.5	139.6	97.97	917	135.615.0	147.89	121.99	127	21.715.0	170.98	133.91	62	8.988.7	144.98	95.41
**Inpatient**	4089	35.860.816.4	8.770.1	3743.49	917	7.858.568.6	8569.87	3466.17	127	1.228.498.4	9673.22	4737.91	62	611.472.2	9862.45	5153.79
Hospitalizations (>24 hours)	4089	32.019.274.2	7.830.6	3494.90	917	6.909.818.9	7535.24	3250.58	127	1.098.049.7	8646.06	4643.93	62	546.871.8	8820.51	4871.68
Investigations[2] (within hospital)	4089	431.046.0	105.4	90.82	917	113.025.2	123.26	105.17	127	15.578.7	122.67	109.68	62	7.569.7	122.09	96.33

Prescriptions (within hospital)	4089	3.410.496.2	834.1	712.29	917	835.724.5	911.37	707.73	127	114.870.0	904.49	662.10	62	57.030.7	919.85	710.55
**Pharmacy & Investigation (global)**	4089	4.667.768.8	1.141.5	741.63	917	1.140.101.2	1243.29	746.28	127	160.563.9	1264.28	698.99	62	78.129.7	1260.16	768.62
Prescriptions (global)	4089	3.981.481.7	973.7	730.37	917	971.339.6	1059.26	731.83	127	136.585.0	1075.47	698.52	62	66.019.3	1064.83	738.76
Investigations[2] (global)	4089	686.287.1	167.8	107.73	917	168.761.6	184.04	119.98	127	23.978.9	188.81	124.73	62	12.110.3	195.33	112.57
**Indirect cost**																
Cost of absence from work	4089	22.935.699.6	5.609.1	4278.92	917	5.002.214.4	5454.98	3816.29	127	851.469.9	6704.49	5163.57	62	371.610.3	5993.71	5475.02
**Total Overall cost**	4089	64.742.812.9	15.833.4	7002.63	917	14.204.913.7	15490.64	6214.20	127	2.260.386.3	17798.32	9286.10	62	1.075.454.6	17346.04	9198.83
**Bleeding-related costs**																
**Outpatient**	4089	2.544.507.1	622.3	213.96	917	574.412.0	626.40	205.87	127	80.127.3	630.92	232.17	62	39.215.9	632.51	261.23
GPs visits	4089	1.119.535.2	273.8	115.45	917	251.222.2	273.96	106.01	127	33.214.2	261.53	106.13	62	16.607.1	267.86	124.25
Specialist visits	4089	1.286.814.8	314.7	129.37	917	292.898.8	319.41	130.95	127	41.876.5	329.74	137.11	62	20.346.8	328.17	162.31
Investigations[2] (outpatient)	4089	104.775.4	25.6	35.85	917	22.192.4	24.20	34.70	127	3.405.5	26.81	35.63	62	1.816.3	29.29	39.46
Prescriptions (outpatient)	4089	33.381.6	8.2	15.23	917	8.098.6	8.83	14.91	127	1.631.2	12.84	23.34	62	445.8	7.19	12.82
**Inpatient - Bleeding episode (hospital+all other costs in bleeding event hospitalization)**	4089	30.560.479.3	7.473.8	2432.70	917	6.843.144.9	7462.54	2165.10	127	1.064.482.5	8381.75	3948.39	62	494.634.0	7977.97	2888.52
Hospitalizations (>24 hours)	4089	27.377.322.7	6.695.4	2366.87	917	6.026.355.4	6571.82	2105.16	127	955.949.2	7527.16	3914.08	62	447.832.1	7223.10	2849.67
Investigations[2] (within hospital)	4089	378.682.8	92.6	75.26	917	99.881.0	108.92	87.60	127	13.639.1	107.39	91.30	62	6.202.1	100.03	60.12
Prescriptions (within hospital)	4089	2.804.473.8	685.9	551.94	917	716.908.5	781.80	597.92	127	94.894.3	747.20	586.00	62	40.599.8	654.84	477.74
**Pharmacy & Investigation (global)**	4089	3.321.313.6	812.3	561.05	917	847.080.5	923.75	608.25	127	113.570.0	894.25	581.71	62	49.064.0	791.35	491.38
Prescriptions	4089	2.837.855.4	694.0	553.08	917	725.007.1	790.63	600.10	127	96.525.5	760.04	582.64	62	41.045.6	662.03	479.12
Investigations[2] (global)	4089	483.458.2	118.2	81.79	917	122.073.4	133.12	92.89	127	17.044.6	134.21	98.58	62	8.018.4	129.33	66.59
**Indirect cost**																
Cost of absence from work	4089	19.744.948.4	4.828.8	3181.60	917	4.432.466.7	4833.66	2928.03	127	754.730.6	5942.76	4533.25	62	291.039.9	4694.19	3426.28
**Total Overall bleeding cost**	4089	52.849.934.8	12.924.9	4846.83	917	11.850.023.6	12922.60	4290.57	127	1.899.340.5	14955.44	8110.34	62	824.889.8	13304.67	5090.40
**3 years since index date**																
**All-cause costs**																
**Outpatient**	4089	8.196.787.4	2.004.6	689.38	917	1.842.483.9	2009.25	634.31	127	251.008.5	1976.44	658.16	62	124.983.8	2015.87	856.35
GPs visit	4089	3.187.020.5	779.4	321.70	917	715.835.7	780.63	298.62	127	97.077.7	764.39	315.84	62	46.807.6	754.96	356.42
Specialist visit	4089	3.882.210.7	949.4	424.11	917	873.491.5	952.55	396.55	127	112.853.6	888.61	425.44	62	60.330.5	973.07	519.59
Investigations[2] (outpatient)	4089	389.417.3	95.2	74.35	917	87.237.2	95.13	73.76	127	12.486.8	98.32	68.38	62	5.959.6	96.12	81.31
Prescriptions (outpatient)	4089	738.138.9	180.5	121.80	917	165.919.5	180.94	146.72	127	28.590.5	225.12	165.66	62	11.886.1	191.71	139.47
**Inpatient**	4089	37.410.614.6	9.149.1	4108.57	917	8.101.540.5	8834.83	3722.84	127	1.241.676.4	9776.98	4737.71	62	663.189.4	10696.60	5984.09
Hospitalizations (>24 hours)	4089	33.234.305.7	8.127.7	3790.91	917	7.069.143.8	7708.99	3410.50	127	1.105.944.2	8708.22	4647.90	62	591.368.0	9538.19	5533.83
Investigations[2] (within hospital)	4089	471.979.6	115.4	102.15	917	121.354.2	132.34	115.84	127	17.363.5	136.72	123.52	62	8.592.1	138.58	123.68
Prescriptions (within hospital)	4089	3.704.329.3	905.9	812.49	917	911.042.5	993.50	832.69	127	118.368.7	932.04	673.38	62	63.229.3	1019.83	843.62
**Pharmacy & Investigation (global)**	4089	5.303.865.1	1.297.1	848.15	917	1.285.553.4	1401.91	881.77	127	176.809.4	1392.20	726.19	62	89.667.1	1446.24	915.12
Prescriptions (global)	4089	4.442.468.2	1.086.4	833.64	917	1.076.962.0	1174.44	863.41	127	146.959.1	1157.16	717.57	62	75.115.4	1211.54	882.89
Investigations[2] (global)	4089	861.396.9	210.7	125.53	917	208.591.4	227.47	136.65	127	29.850.2	235.04	141.38	62	14.551.7	234.71	139.04
**Indirect cost**																
Cost of absence from work	4089	23.949.132.4	5.857.0	4637.72	917	5.115.122.5	5578.11	4004.14	127	851.469.9	6704.49	5163.57	62	426.694.1	6882.16	6683.96
**Total Overall cost**	4089	69.556.534.4	17.010.6	7616.47	917	15.059.146.9	16422.19	6547.90	127	2.344.154.8	18457.91	9204.38	62	1.214.867.4	19594.63	11279.27
**Bleeding-related costs**																
**Outpatient**	4089	3.031.754.7	741.4	265.10	917	683.622.6	745.50	255.97	127	96.527.1	760.06	304.12	62	46.735.1	753.79	305.27
GPs visits	4089	1.305.996.2	319.4	128.67	917	295.080.2	321.79	118.16	127	38.664.4	304.44	119.27	62	19.300.1	311.29	139.73
Specialist visits	4089	1.535.234.6	375.5	170.59	917	347.314.6	378.75	170.89	127	51.103.5	402.39	196.18	62	24.605.4	396.86	199.90
Investigations[2] (outpatient)	4089	156.311.8	38.2	51.42	917	32.976.5	35.96	49.29	127	5.108.2	40.22	51.62	62	2.383.8	38.45	45.70

Prescriptions (outpatient)	4089	34.212.1	8.4	16.18	917	8.251.3	9.00	15.15	127	1.651.0	13.00	23.46	62	445.8	7.19	12.82
**Inpatient - Bleeding episode (hospital+all other costs in bleeding event hospitalization)**	4089	31.273.409.0	7.648.2	2674.46	917	6.942.052.4	7570.40	2330.05	127	1.081.687.8	8517.23	4056.45	62	508.324.0	8198.77	3091.26
Hospitalizations (>24 hours)	4089	28.021.081.3	6.852.8	2614.91	917	6.106.017.8	6658.69	2265.05	127	970.302.8	7640.18	3995.39	62	461.468.0	7443.03	3039.95
Investigations[2] (within hospital)	4089	394.035.5	96.4	80.56	917	103.851.3	113.25	93.77	127	13.952.9	109.86	91.37	62	6.256.3	100.91	59.38
Prescriptions (within hospital)	4089	2.858.292.1	699.0	568.01	917	732.183.3	798.46	613.66	127	97.432.1	767.18	634.56	62	40.599.8	654.84	477.74
**Pharmacy & Investigation (global)**	4089	3.442.851.6	842.0	578.68	917	877.262.4	956.67	625.27	127	118.144.2	930.27	631.96	62	49.685.7	801.38	488.22
Prescriptions	4089	2.892.504.3	707.4	569.18	917	740.434.6	807.45	615.94	127	99.083.1	780.18	631.13	62	41.045.6	662.03	479.12
Investigations[2] (global)	4089	550.347.3	134.6	95.52	917	136.827.7	149.21	106.04	127	19.061.1	150.09	104.41	62	8.640.1	139.36	73.10
**Indirect cost**																
Cost of absence from work	4089	20.119.299.2	4.920.3	3315.62	917	4.470.833.6	4875.50	2985.46	127	754.730.6	5942.76	4533.25	62	303.646.2	4897.52	3703.60
**Total Overall bleeding cost**	4089	54.424.462.9	13.310.0	5153.05	917	12.096.508.6	13191.39	4425.83	127	1.932.945.5	15220.04	8094.63	62	858.705.3	13850.09	5678.08

	Overall group				Genitourinary				Respiratory				Other MB			
**Cumulative costs**	**No. Patients**	**Total cost (Eur)**	**Mean costs per patient (Eur)**	**SD costs per patient (Eur)**	**No. Patients**	**Total cost (Eur)**	**Mean costs per patient (Eur)**	**SD costs per patient (Eur)**	**No. Patients**	**Total cost (Eur)**	**Mean costs per patient (Eur)**	**SD costs per patient (Eur)**	**No. Patients**	**Total cost (Eur)**	**Mean costs per patient (Eur)**	**SD costs per patient (Eur)**
**6 months since index date**																
**All-cause costs**																
**Outpatient**	4089	1.883.101.2	460.5	204.09	1130	479.878.7	424.67	188.52	211	110.568.0	524.02	208.98	1704	813.178.9	477.22	215.29
GPs visit	4089	768.606.4	188.0	102.61	1130	193.065.3	170.85	97.04	211	46.679.4	221.23	98.57	1704	334.514.0	196.31	108.10
Specialist visit	4089	896.204.2	219.2	142.49	1130	225.470.6	199.53	135.67	211	53.232.8	252.29	154.08	1704	389.664.3	228.68	145.39
Investigations[2] (outpatient)	4089	45.406.5	11.1	23.78	1130	12.543.5	11.10	24.23	211	3.008.2	14.26	25.89	1704	18.559.9	10.89	23.35
Prescriptions (outpatient)	4089	172.884.1	42.3	25.93	1130	48.799.2	43.19	24.89	211	7.647.7	36.24	22.83	1704	70.440.7	41.34	25.71
**Inpatient (hospital+all other costs in hospitalization)**	4089	32.839.020.2	8.031.1	2772.13	1130	9.214.733.0	8154.63	2693.04	211	1.584.062.4	7507.40	2529.99	1704	13.433.837.2	7883.71	2672.20
Hospitalsations (>24 hours)	4089	29.397.590.7	7.189.4	2726.77	1130	8.271.257.1	7319.70	2649.58	211	1.440.382.9	6826.46	2466.66	1704	12.075.676.5	7086.66	2632.25
Investigations[2] (within hospital)	4089	360.746.9	88.2	67.86	1130	94.391.8	83.53	62.49	211	18.113.2	85.84	63.82	1704	141.401.2	82.98	62.59
Prescriptions (within hospital)	4089	3.080.682.6	753.4	508.31	1130	849.084.1	751.40	510.52	211	125.566.4	595.10	458.88	1704	1.216.759.5	714.06	480.94
**Pharmacy & Investigation (global)**	4089	3.659.720.0	895.0	518.92	1130	1.004.818.6	889.22	519.72	211	154.335.4	731.45	472.86	1704	1.447.161.3	849.27	491.14
Prescriptions (global)	4089	3.253.566.7	795.7	512.51	1130	897.883.3	794.59	514.32	211	133.214.0	631.35	462.85	1704	1.287.200.2	755.40	485.67
Investigations[2] (global)	4089	406.153.3	99.3	71.70	1130	106.935.3	94.63	66.67	211	21.121.3	100.10	67.89	1704	159.961.1	93.87	67.08
**Indirect cost**																
Cost of absence from work	4089	21.077.922.2	5.154.8	3587.49	1130	6.464.265.2	5720.59	3335.09	211	889.562.7	4215.94	3606.83	1704	8.084.716.3	4744.55	3593.40
**Total Overall cost**	4089	55.800.043.6	13.646.4	5699.97	1130	16.158.876.8	14299.89	5540.07	211	2.584.193.2	12247.36	5381.16	1704	22.331.732.4	13105.48	5468.09
**Bleeding-related costs**																
**Outpatient**	4089	1.655.696.6	404.9	194.35	1130	417.891.4	369.82	178.85	211	98.606.2	467.33	194.10	1704	718.314.9	421.55	205.51
GPs visits	4089	717.951.6	175.6	92.75	1130	180.882.5	160.07	87.87	211	43.024.5	203.91	87.51	1704	312.456.8	183.37	97.65
Specialist visits	4089	896.204.2	219.2	142.49	1130	225.470.6	199.53	135.67	211	53.232.8	252.29	154.08	1704	389.664.3	228.68	145.39
Investigations[2] (outpatient)	4089	16.119.3	3.9	14.43	1130	4.029.8	3.57	13.78	211	1.135.2	5.38	16.67	1704	6.867.7	4.03	14.58
Prescriptions (outpatient)	4089	25.421.5	6.2	9.82	1130	7.508.4	6.64	9.99	211	1.213.7	5.75	8.40	1704	9.326.1	5.47	9.21
**Inpatient - Bleeding episode (hospital+all other costs in bleeding event hospitalization)**	4089	29.182.574.8	7.136.8	1908.12	1130	8.136.897.1	7200.79	1693.58	211	1.408.755.6	6676.57	1689.00	1704	11.968.274.9	7023.64	1854.84
Hospitalizations (>24 hours)	4089	26.156.549.8	6.396.8	1832.35	1130	7.300.954.3	6461.02	1603.36	211	1.278.905.0	6061.16	1598.75	1704	10.783.135.6	6328.13	1782.06
Investigations[2] (within hospital)	4089	341.484.8	83.5	60.73	1130	89.011.5	78.77	55.35	211	17.165.5	81.35	57.50	1704	134.373.1	78.86	56.52
Prescriptions (within hospital)	4089	2.684.540.2	656.5	529.04	1130	746.931.3	661.00	531.41	211	112.685.1	534.05	467.81	1704	1.050.766.1	616.65	499.70
**Pharmacy & Investigation (global)**	4089	3.067.565.8	750.2	536.27	1130	847.481.1	749.98	538.92	211	132.199.5	626.54	475.47	1704	1.201.333.1	705.01	505.59
Prescriptions	4089	2.709.961.7	662.7	531.13	1130	754.439.7	667.65	533.50	211	113.898.8	539.80	470.29	1704	1.060.092.2	622.12	501.59
Investigations[2] (global)	4089	357.604.1	87.5	62.42	1130	93.041.3	82.34	56.57	211	18.300.6	86.73	58.79	1704	141.240.8	82.89	58.53
**Indirect cost**																
Cost of absence from work	4089	19.174.652.7	4.689.3	2961.47	1130	5.805.725.7	5137.81	2601.07	211	820.776.4	3889.94	3121.61	1704	7.425.080.6	4357.44	3028.26
**Total Overall bleeding cost**	4089	50.012.924.1	12.231.1	4304.38	1130	14.360.514.2	12708.42	3841.67	211	2.328.138.2	11033.83	4367.53	1704	20.111.670.3	11802.62	4231.27
**1 year since index date**																
**All-cause costs**																
**Outpatient**	4089	3.303.129.4	807.8	319.53	1130	849.391.2	751.67	288.56	211	190.415.3	902.44	316.90	1704	1.417.332.9	831.77	344.06

GPs visit	4089	1.277.078.0	312.3	148.44	1130	325.793.7	288.31	139.56	211	74.186.8	351.60	144.38	1704	554.125.0	325.19	158.39
Specialist visit	4089	1.582.789.3	387.1	221.73	1130	400.784.0	354.68	205.10	211	94.399.5	447.39	227.69	1704	681.616.7	400.01	231.46
Investigations[2] (outpatient)	4089	105.910.6	25.9	38.49	1130	28.435.8	25.16	38.25	211	6.583.9	31.20	44.00	1704	43.760.5	25.68	37.82
Prescriptions (outpatient)	4089	337.351.5	82.5	37.70	1130	94.377.7	83.52	36.09	211	15.245.0	72.25	34.04	1704	137.830.7	80.89	36.60
**Inpatient**	4089	34.039.190.2	8.324.6	3106.92	1130	9.535.726.2	8438.70	3019.55	211	1.651.683.5	7827.88	2829.49	1704	14.032.746.0	8235.18	3101.12
Hospitalizations (>24 hours)	4089	30.452.579.7	7.447.4	2998.00	1130	8.557.611.2	7573.11	2924.83	211	1.499.232.6	7105.37	2675.66	1704	12.601.735.6	7395.38	2971.71
Investigations[2] (within hospital)	4089	381.223.6	93.2	74.45	1130	99.176.2	87.77	68.00	211	19.284.3	91.39	71.77	1704	149.544.8	87.76	68.92
Prescriptions (within hospital)	4089	3.205.386.9	783.9	569.73	1130	878.938.7	777.82	554.57	211	133.166.5	631.12	520.21	1704	1.281.465.6	752.03	565.99
**Pharmacy & Investigation (global)**	4089	4.029.872.6	985.5	585.87	1130	1.100.928.4	974.27	568.67	211	174.279.8	825.97	537.88	1704	1.612.601.5	946.36	579.62
Prescriptions (global)	4089	3.542.738.4	866.4	578.39	1130	973.316.4	861.34	561.36	211	148.411.6	703.37	530.28	1704	1.419.296.3	832.92	573.42
Investigations[2] (global)	4089	487.134.2	119.1	83.04	1130	127.612.0	112.93	77.09	211	25.868.3	122.60	79.45	1704	193.305.3	113.44	78.60
**Indirect cost**																
Cost of absence from work	4089	21.703.027.7	5.307.7	3764.51	1130	6.670.075.9	5902.72	3519.43	211	908.198.0	4304.26	3712.46	1704	8.399.872.5	4929.50	3826.43
**Total Overall cost**	4089	59.045.347.4	14.440.0	6063.03	1130	17.055.193.3	15093.09	5953.79	211	2.750.296.9	13034.58	5557.35	1704	23.849.951.5	13996.45	5965.63
**Bleeding-related costs**																
**Outpatient**	4089	2.028.556.7	496.1	205.05	1130	515.243.8	455.97	178.28	211	119.536.9	566.53	204.06	1704	875.876.8	514.01	224.68
GPs visits	4089	896.012.9	219.1	106.74	1130	228.203.1	201.95	101.40	211	52.129.6	247.06	105.12	1704	389.464.9	228.56	112.93
Specialist visits	4089	1.039.577.9	254.2	134.15	1130	263.325.0	233.03	117.98	211	61.750.1	292.65	138.68	1704	447.628.9	262.69	144.04
Investigations[2] (outpatient)	4088	61.866.3	15.1	28.73	1130	15.211.2	13.46	27.57	211	4.029.8	19.10	32.09	1704	26.789.8	15.72	28.89
Prescriptions (outpatient)	4089	31.099.6	7.6	14.59	1130	8.504.5	7.53	12.99	211	1.627.4	7.71	18.18	1704	11.993.2	7.04	14.56
**Inpatient - Bleeding episode (hospital+all other costs in bleeding event hospitalization)**	4089	29.738.351.2	7.272.8	2126.76	1130	8.279.966.1	7327.40	1910.36	211	1.440.492.1	6826.98	1951.41	1704	12.210.830.1	7165.98	2092.99
Hospitalizations (>24 hours)	4089	26.617.300.1	6.509.5	2056.88	1130	7.423.677.5	6569.63	1839.52	211	1.308.329.9	6200.62	1840.17	1704	10.985.521.2	6446.90	2023.38
Investigations[2] (within hospital)	4089	361.007.9	88.3	68.47	1130	93.039.4	82.34	61.68	211	18.059.6	85.59	64.10	1704	141.712.4	83.16	63.52
Prescriptions (within hospital)	4089	2.760.043.2	675.0	551.81	1130	763.249.2	675.44	550.33	211	114.102.6	540.77	470.94	1704	1.083.596.4	635.91	521.77
**Pharmacy & Investigation (global)**	4089	3.214.017.1	786.0	559.78	1130	880.004.3	778.76	557.82	211	137.819.4	653.17	481.93	1704	1.264.091.8	741.84	527.31
Prescriptions	4089	2.791.142.8	682.6	552.96	1130	771.753.8	682.97	551.59	211	115.730.0	548.48	471.68	1704	1.095.589.6	642.95	522.44
Investigations[2] (global)	4089	422.874.3	103.4	73.72	1130	108.250.5	95.80	67.92	211	22.089.4	104.69	70.08	1704	168.502.3	98.89	68.93
**Indirect cost**																
Cost of absence from work	4089	19.380.463.4	4.739.7	3042.33	1130	5.888.488.5	5211.05	2712.98	211	828.175.7	3925.00	3183.60	1704	7.502.910.5	4403.12	3101.29
**Total Overall bleeding cost**	4089	51.147.371.3	12.508.5	4499.23	1130	14.683.698.4	12994.42	4128.98	211	2.388.204.7	11318.51	4511.35	1704	20.589.617.3	12083.11	4408.69
**2 years since index date**																
**All-cause costs**																
**Outpatient**	4089	5.946.296.9	1.454.2	530.12	1130	1.531.863.5	1355.63	471.12	211	340.513.7	1613.81	521.01	1704	2.549.371.0	1496.11	576.67
GPs visit	4089	2.355.512.3	576.1	253.26	1130	602.728.0	533.39	231.14	211	136.319.1	646.06	249.78	1704	1.017.712.6	597.25	272.51
Specialist visit	4089	2.764.558.0	676.1	334.57	1130	705.039.2	623.93	310.39	211	164.430.3	779.29	342.38	1704	1.190.759.1	698.80	351.98
Investigations[2] (outpatient)	4089	255.241.2	62.4	59.95	1130	67.088.1	59.37	58.70	211	14.019.2	66.44	63.38	1704	109.997.2	64.55	61.26
Prescriptions (outpatient)	4089	570.985.5	139.6	97.97	1130	157.008.3	138.95	84.99	211	25.745.1	122.01	86.82	1704	230.902.0	135.51	88.47
**Inpatient**	4089	35.860.816.4	8.770.1	3743.49	1130	10.003.643.4	8852.78	3549.59	211	1.712.954.4	8118.27	3386.70	1704	15.057.151.5	8836.36	3950.35
Hospitalizations (>24 hours)	4089	32.019.274.2	7.830.6	3494.90	1130	8.970.277.0	7938.30	3384.52	211	1.553.776.3	7363.87	3189.12	1704	13.487.352.2	7915.11	3613.12
Investigations[2] (within hospital)	4089	431.046.0	105.4	90.82	1130	112.185.6	99.28	82.26	211	21.924.3	103.91	90.24	1704	168.332.2	98.79	84.90
Prescriptions (within hospital)	4089	3.410.496.2	834.1	712.29	1130	921.180.8	815.20	638.04	211	137.253.9	650.49	538.99	1704	1.401.467.1	822.46	776.02
**Pharmacy & Investigation (global)**	4089	4.667.768.8	1.141.5	741.63	1130	1.257.462.8	1112.80	659.28	211	198.942.5	942.86	582.75	1704	1.910.698.5	1121.30	801.25
Prescriptions (global)	4089	3.981.481.7	973.7	730.37	1130	1.078.189.1	954.15	650.38	211	162.999.0	772.51	564.05	1704	1.632.369.1	957.96	791.49
Investigations[2] (global)	4089	686.287.1	167.8	107.73	1130	179.273.7	158.65	99.91	211	35.943.5	170.35	104.12	1704	278.329.4	163.34	103.72

**Indirect cost**																
Cost of absence from work	4089	22.935.699.6	5.609.1	4278.92	1130	7.082.245.4	6267.47	4110.21	211	939.987.7	4454.92	3943.61	1704	9.059.782.2	5316.77	4515.12
**Total Overall cost**	4089	64.742.812.9	15.833.4	7002.63	1130	18.617.752.4	16475.89	6976.19	211	2.993.455.9	14186.99	6008.58	1704	26.666.304.7	15649.24	7269.80
**Bleeding-related costs**																
**Outpatient**	4089	2.544.507.1	622.3	213.96	1130	667.003.8	590.27	194.86	211	141.916.3	672.59	218.38	1704	1.081.047.7	634.42	225.61
GPs visits	4089	1.119.535.2	273.8	115.45	1130	286.616.4	253.64	106.90	211	64.504.7	305.71	114.84	1704	483.977.8	284.02	124.12
Specialist visits	4089	1.286.814.8	314.7	129.37	1130	344.238.9	304.64	122.27	211	69.084.4	327.41	138.44	1704	538.716.2	316.15	131.03
Investigations[2] (outpatient)	4089	104.775.4	25.6	35.85	1130	27.300.6	24.16	35.04	211	6.640.7	31.47	38.40	1704	45.236.2	26.55	36.60
Prescriptions (outpatient)	4089	33.381.6	8.2	15.23	1130	8.847.8	7.83	13.46	211	1.686.5	7.99	18.29	1704	13.117.5	7.70	15.28
**Inpatient - Bleeding episode (hospital+all other costs in bleeding event hospitalization)**	4089	30.560.479.3	7.473.8	2432.70	1130	8.510.102.9	7531.06	2267.33	211	1.487.003.2	7047.41	2516.51	1704	12.655.745.8	7427.08	2500.75
Hospitalizations (>24 hours)	4089	27.377.322.7	6.695.4	2366.87	1130	7.642.569.8	6763.34	2205.27	211	1.352.108.3	6408.10	2433.23	1704	11.400.340.0	6690.34	2429.64
Investigations[2] (within hospital)	4089	378.682.8	92.6	75.26	1130	98.223.4	86.92	69.53	211	18.486.9	87.62	65.57	1704	148.452.4	87.12	70.01
Prescriptions (within hospital)	4089	2.804.473.8	685.9	551.94	1130	769.309.7	680.81	550.97	211	116.407.9	551.70	473.22	1704	1.106.953.4	649.62	525.28
**Pharmacy & Investigation (global)**	4089	3.321.313.6	812.3	561.05	1130	903.681.6	799.72	560.86	211	143.222.0	678.78	486.72	1704	1.313.759.5	770.99	531.62
Prescriptions	4089	2.837.855.4	694.0	553.08	1130	778.157.5	688.63	552.09	211	118.094.4	559.69	474.15	1704	1.120.070.9	657.32	526.06
Investigations[2] (global)	4089	483.458.2	118.2	81.79	1130	125.524.1	111.08	76.99	211	25.127.6	119.09	73.80	1704	193.688.6	113.67	76.83
**Indirect cost**																
Cost of absence from work	4089	19.744.948.4	4.828.8	3181.60	1130	6.049.355.2	5353.41	2943.85	211	840.782.0	3984.75	3273.13	1704	7.667.613.9	4499.77	3260.22
**Total Overall bleeding cost**	4089	52.849.934.8	12.924.9	4846.83	1130	15.226.461.9	13474.75	4705.75	211	2.469.701.4	11704.75	4842.69	1704	21.404.407.3	12561.27	4819.31
**3 years since index date**																
**All-cause costs**																
**Outpatient**	4089	8.196.787.4	2.004.6	689.38	1130	2.121.544.3	1877.47	604.55	211	470.813.2	2231.34	687.55	1704	3.510.937.5	2060.41	755.96
GPs visit	4089	3.187.020.5	779.4	321.70	1130	821.569.6	727.05	293.17	211	181.780.2	861.52	317.71	1704	1.370.757.4	804.44	346.93
Specialist visit	4089	3.882.210.7	949.4	424.11	1130	994.625.7	880.20	384.74	211	231.858.5	1098.86	433.90	1704	1.669.381.3	979.68	453.10
Investigations[2] (outpatient)	4089	389.417.3	95.2	74.35	1130	101.937.5	90.21	71.34	211	22.135.7	104.91	79.61	1704	165.620.1	97.19	76.22
Prescriptions (outpatient)	4089	738.138.9	180.5	121.80	1130	203.411.5	180.01	103.54	211	35.038.8	166.06	119.82	1704	305.178.7	179.10	113.79
**Inpatient**	4089	37.410.614.6	9.149.1	4108.57	1130	10.477.239.0	9271.89	3903.37	211	1.764.753.6	8363.76	3617.40	1704	15.825.405.1	9287.21	4416.21
Hospitalizations (>24 hours)	4089	33.234.305.7	8.127.7	3790.91	1130	9.353.517.9	8277.45	3670.74	211	1.596.119.4	7564.55	3375.82	1704	14.109.580.4	8280.27	4013.41
Investigations[2] (within hospital)	4089	471.979.6	115.4	102.15	1130	122.485.4	108.39	92.70	211	24.081.9	114.13	97.40	1704	186.694.7	109.56	97.89
Prescriptions (within hospital)	4089	3.704.329.3	905.9	812.49	1130	1.001.235.7	886.05	742.77	211	144.552.4	685.08	571.19	1704	1.529.130.0	897.38	872.70
**Pharmacy & Investigation (global)**	4089	5.303.865.1	1.297.1	848.15	1130	1.429.070.1	1264.66	765.34	211	225.808.8	1070.18	628.76	1704	2.186.623.5	1283.23	904.81
Prescriptions (global)	4089	4.442.468.2	1.086.4	833.64	1130	1.204.647.2	1066.06	756.54	211	179.591.2	851.14	607.61	1704	1.834.308.7	1076.47	890.17
Investigations[2] (global)	4089	861.396.9	210.7	125.53	1130	224.422.9	198.60	116.60	211	46.217.5	219.04	124.46	1704	352.314.8	206.76	122.74
**Indirect cost**																
Cost of absence from work	4089	23.949.132.4	5.857.0	4637.72	1130	7.424.258.4	6570.14	4464.57	211	975.614.0	4623.76	4162.76	1704	9.582.667.5	5623.63	5000.70
**Total Overall cost**	4089	69.556.534.4	17.010.6	7616.47	1130	20.023.041.7	17719.51	7573.27	211	3.211.180.9	15218.87	6390.16	1704	28.919.010.1	16971.25	8111.07
**Bleeding-related costs**																
**Outpatient**	4089	3.031.754.7	741.4	265.10	1130	796.589.1	704.95	245.94	211	171.930.7	814.84	268.62	1704	1.283.085.2	752.98	275.51
GPs visits	4089	1.305.996.2	319.4	128.67	1130	335.796.4	297.16	119.57	211	74.635.7	353.72	127.53	1704	561.819.4	329.71	138.19
Specialist visits	4089	1.535.234.6	375.5	170.59	1130	410.247.6	363.05	165.90	211	85.645.7	405.90	178.05	1704	640.923.2	376.13	169.90
Investigations[2] (outpatient)	4089	156.311.8	38.2	51.42	1130	41.376.7	36.62	49.67	211	9.932.7	47.07	62.65	1704	66.917.8	39.27	52.03
Prescriptions (outpatient)	4089	34.212.1	8.4	16.18	1130	9.168.3	8.11	15.80	211	1.716.7	8.14	18.58	1704	13.424.8	7.88	15.95

**Inpatient - Bleeding episode (hospital+all other costs in bleeding event hospitalization)**	4089	31.273.409.0	7.648.2	2674.46	1130	8.722.704.0	7719.21	2541.42	211	1.533.859.8	7269.48	2705.36	1704	12.993.105.0	7625.06	2786.88
Hospitalizations (>24 hours)	4089	28.021.081.3	6.852.8	2614.91	1130	7.834.907.9	6933.55	2492.82	211	1.396.604.5	6618.98	2632.05	1704	11.713.248.3	6873.97	2724.20
Investigations[2] (within hospital)	4089	394.035.5	96.4	80.56	1130	102.996.4	91.15	75.65	211	19.008.1	90.09	67.69	1704	154.226.9	90.51	75.16
Prescriptions (within hospital)	4089	2.858.292.1	699.0	568.01	1130	784.799.7	694.51	569.48	211	118.247.3	560.41	474.37	1704	1.125.629.8	660.58	538.37
**Pharmacy & Investigation (global)**	4089	3.442.851.6	842.0	578.68	1130	938.341.0	830.39	580.95	211	148.904.7	705.71	485.73	1704	1.360.199.3	798.24	547.00
Prescriptions	4089	2.892.504.3	707.4	569.18	1130	793.968.0	702.63	570.78	211	119.963.9	568.55	475.70	1704	1.139.054.6	668.46	539.03
Investigations[2] (global)	4089	550.347.3	134.6	95.52	1130	144.373.0	127.76	92.39	211	28.940.8	137.16	90.69	1704	221.144.7	129.78	90.48
**Indirect cost**																
Cost of absence from work	4089	20.119.299.2	4.920.3	3315.62	1130	6.191.038.4	5478.80	3135.78	211	869.009.0	4118.53	3463.68	1704	7.833.687.5	4597.23	3406.81
**Total Overall bleeding cost**	4089	54.424.462.9	13.310.0	5153.05	1130	15.710.331.5	13902.95	5144.96	211	2.574.799.6	12202.84	5227.62	1704	22.109.877.8	12975.28	5163.60

1. Index date: day of the first major bleeding; 2. Laboratory/radiology investigations. Quantitative variables are presented as mean and (standard deviation); qualitative variables are presented by their absolute frequencies.

GIB: gastrointestinal bleeding; GP: general practitioners; HCRU: healthcare resource utilization; ICH: intracranial hemorrhage; MB: major bleeding.

Supplementary Figure S1. Cumulative incidence (per 100 patient-years) curves for events for all bleedings.

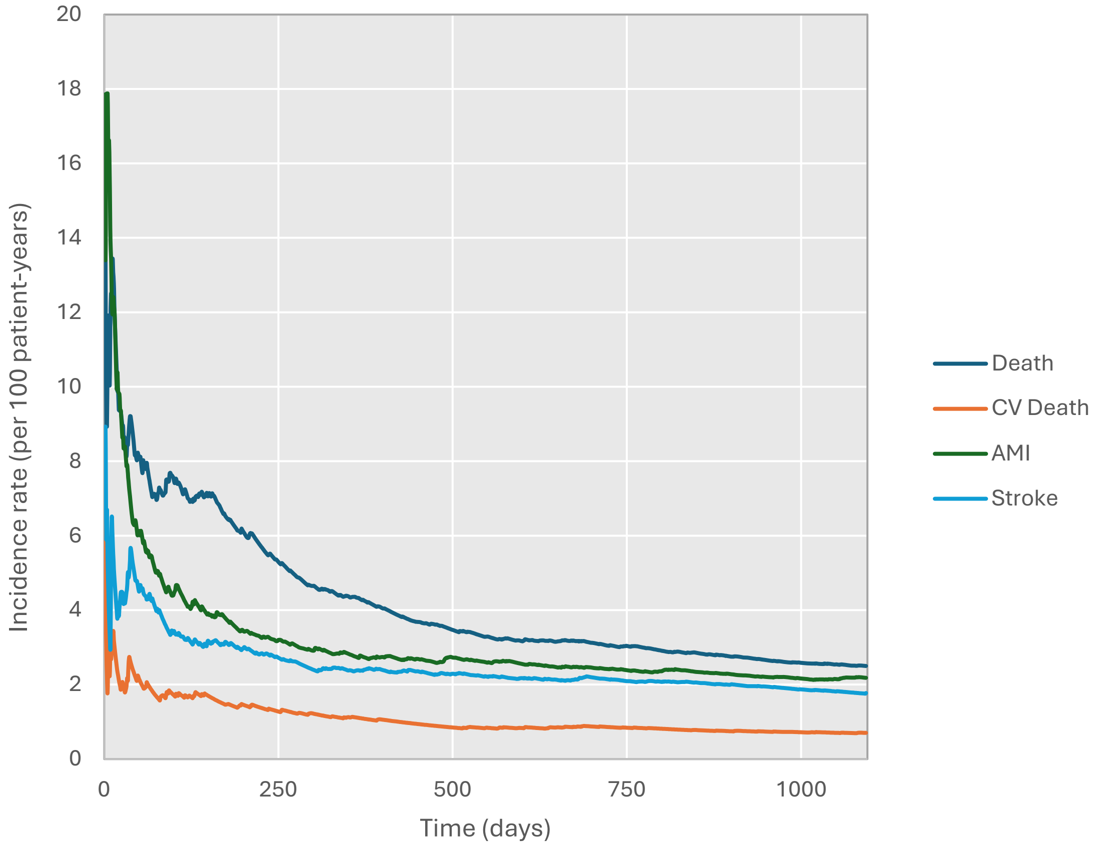

AMI: acute myocardial infarction; CV: cardiovascular.

Supplementary Figure S2. Cumulative incidence (per 100 patient-years) curves for events for gastrointestinal bleedings.

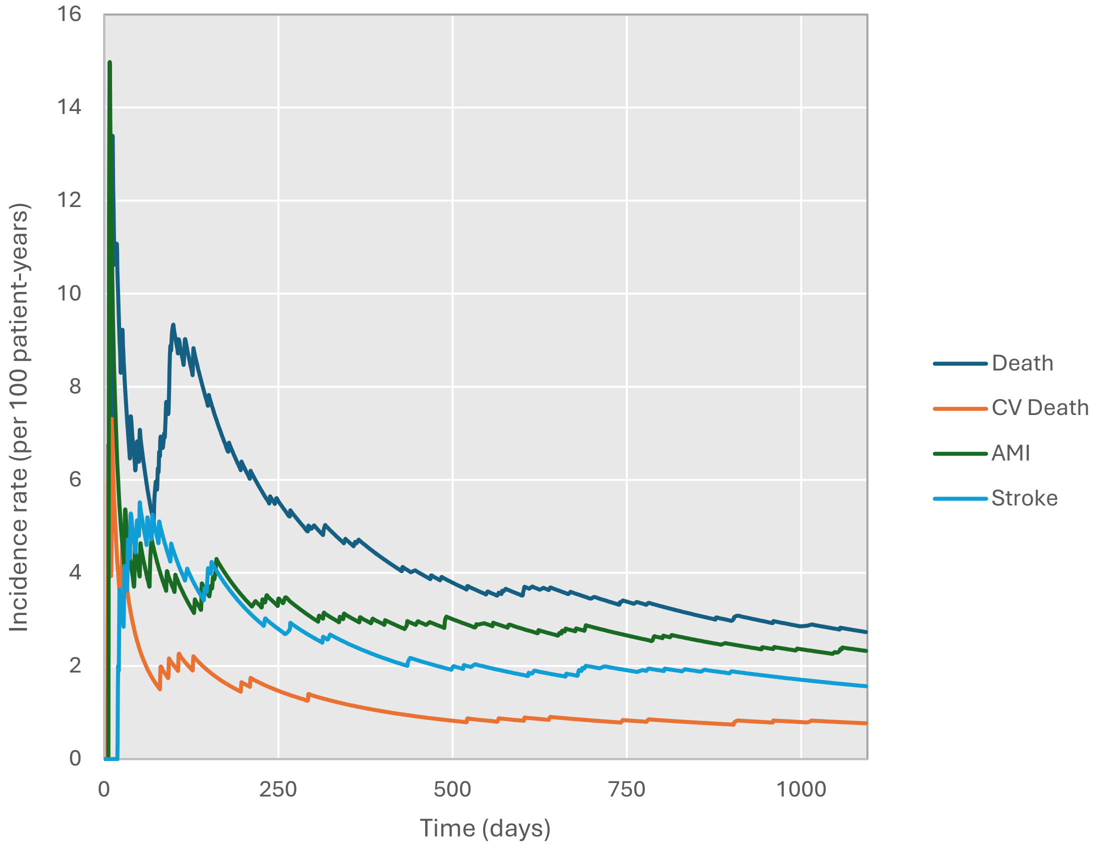

AMI: acute myocardial infarction; CV: cardiovascular.

Supplementary Figure S3. Cumulative incidence (per 100 patient-years) curves for events for intracranial bleedings.

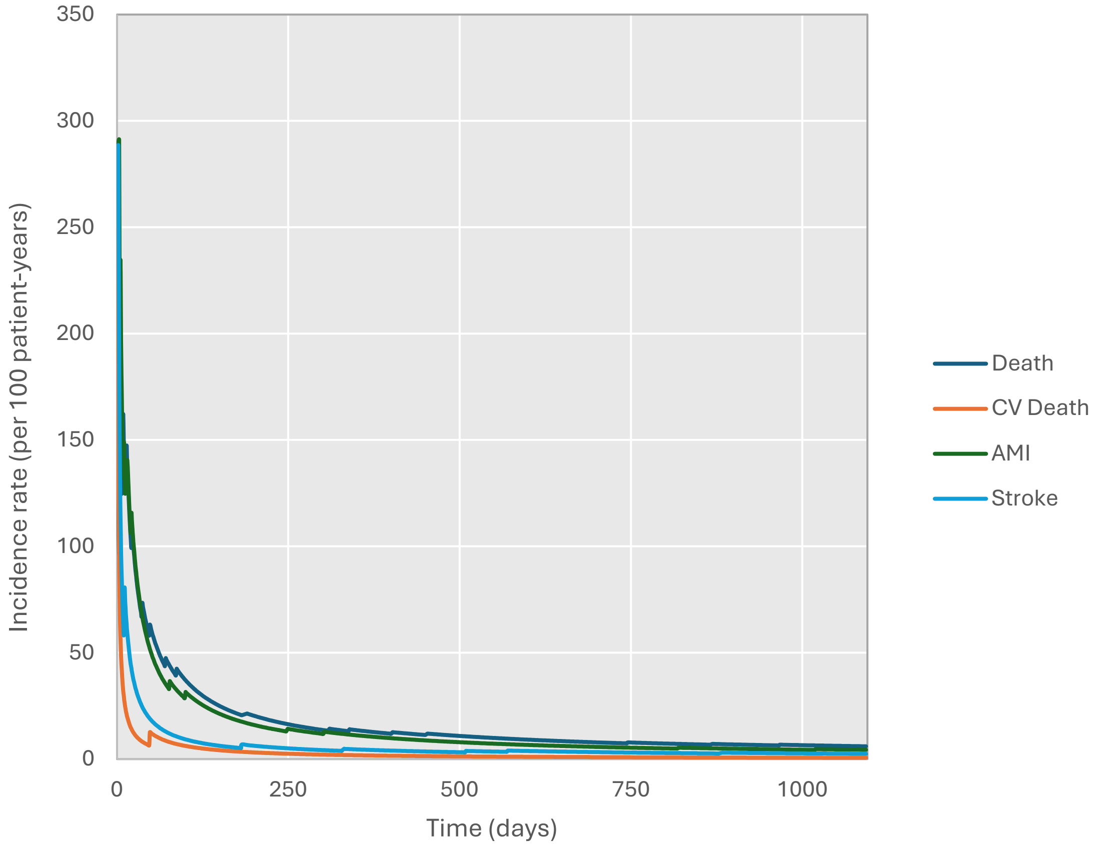

AMI: acute myocardial infarction; CV: cardiovascular.

Supplementary Figure S4. Cumulative incidence (per 100 patient-years) curves for events for genitourinary bleedings.

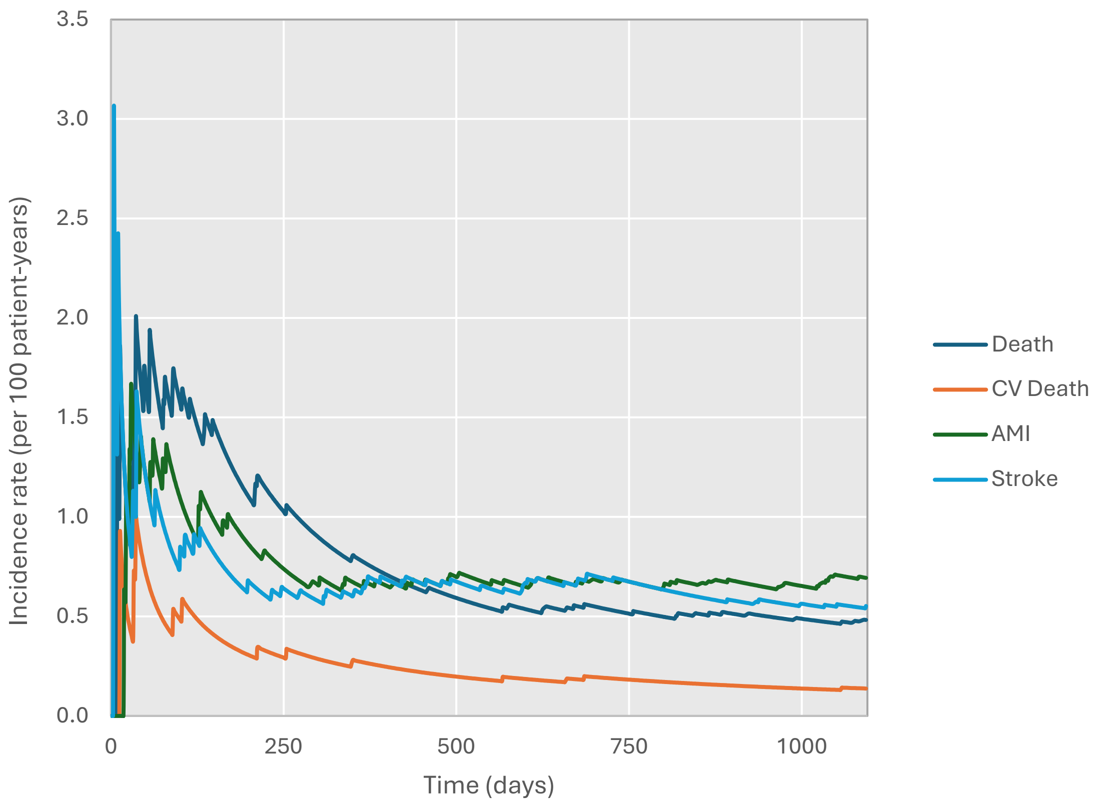

AMI: acute myocardial infarction; CV: cardiovascular.

Supplementary Figure S5. Cumulative incidence (per 100 patient-years) curves for events for respiratory bleedings.

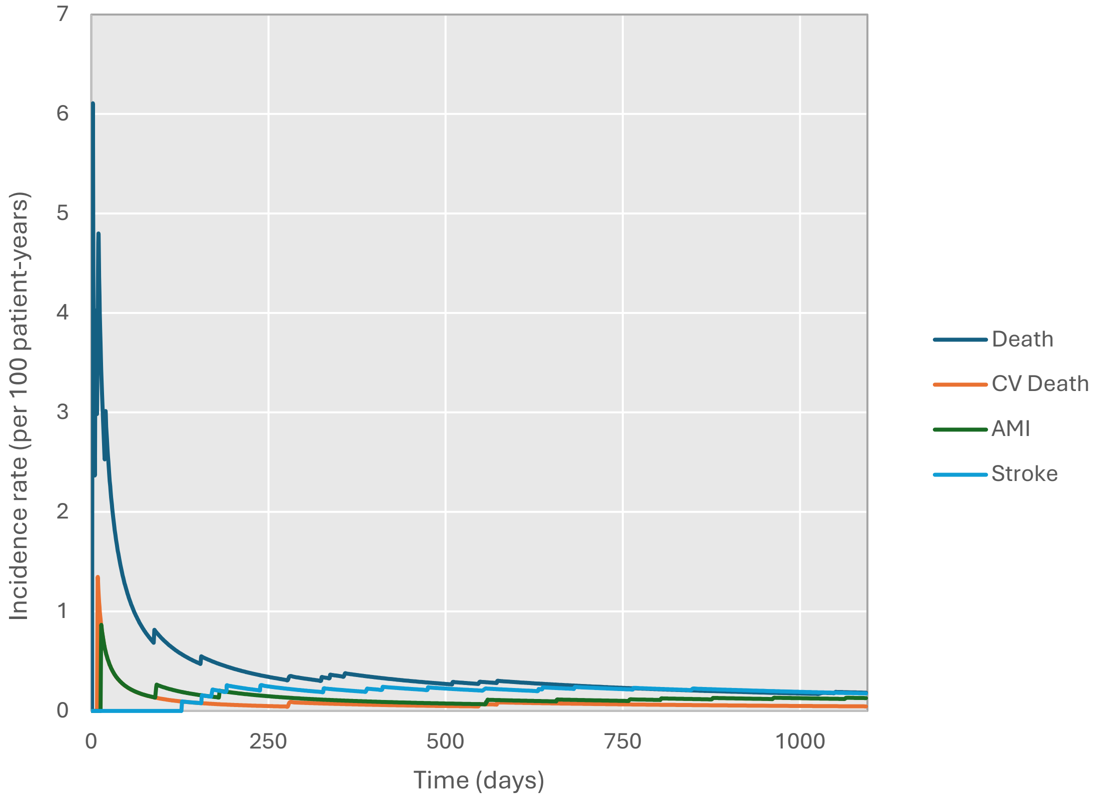

AMI: acute myocardial infarction; CV: cardiovascular.

Supplementary Figure S6. Cumulative incidence (per 100 patient-years) curves for events for other bleedings.

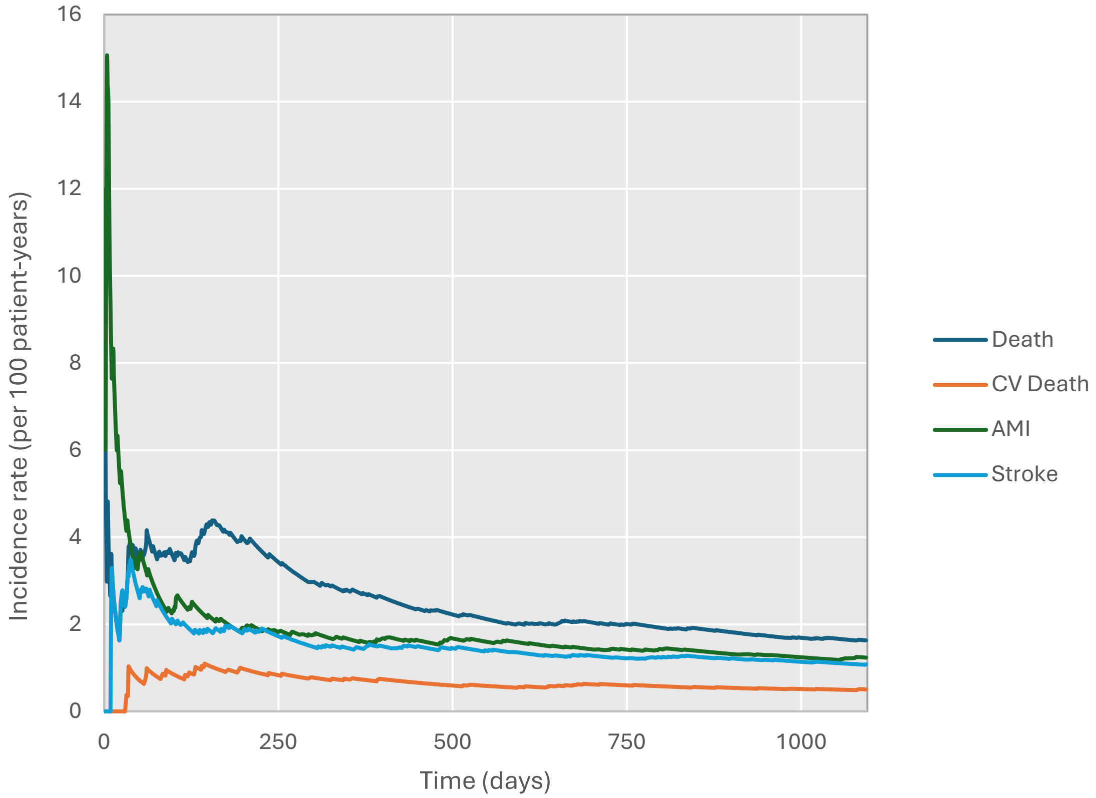

AMI: acute myocardial infarction; CV: cardiovascular.

Supplementary Figure S7. Cumulative incidence (per 100 patient-years) curves for events for trauma bleedings.

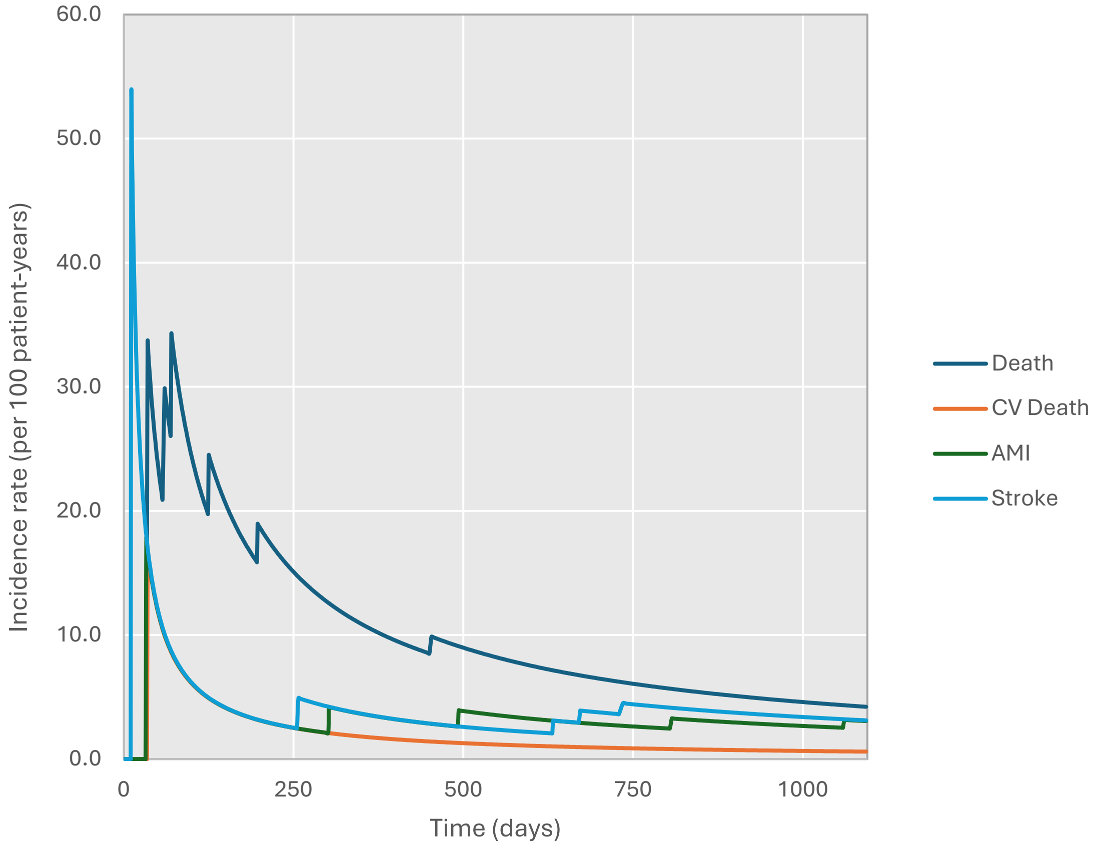

AMI: acute myocardial infarction; CV: cardiovascular.
